# Fragment-based active-site exploration of urethane hydrolases reveals a diversity of urethane binding modes

**DOI:** 10.1107/S2059798326010624

**Published:** 2026-10-26

**Authors:** Deniz Bicer, Diana Kochubei, Rosie Graham, Samuel Peña-Díaz, Laura Rotilio, Nikolaj L. Villadsen, Andreas Sommerfeldt, Martin B. Johansen, Alexander Sandahl, Søren S. Thirup, J. Preben Morth, Daniel E. Otzen

**Affiliations:** a EnZync Center for Enzymatic Deconstruction of Thermoset Plastics, Denmark; b Department of Molecular Biology and Genetics Aarhus University Universitetsbyen 81 8000Aarhus Denmark; c School of Science Constructor University Campus Ring 1 28759Bremen Germany; d Interdisciplinary Nanoscience Center (iNANO) Aarhus University Gustav Wieds Vej 14 8000Aarhus Denmark; e Biotechnology and Biomedicine Technical University of Denmark Søltofts Plads 2800Kongens Lyngby Denmark; f Danish Technological Institute Kongsvang Alle 29 8000Aarhus Denmark; McGill University, Canada

**Keywords:** fragments, urethanases, crystallography, polyurethane, amidases, esterases

## Abstract

Recent advances in the discovery, characterization and engineering of urethanases provide new opportunities for the sustainable biocatalytic degradation of polyurethane waste. A mechanistic understanding of enzyme–plastic inter­actions is essential for structure-based engineering to enhance urethanase activity. However, the extremely complex and hydrophobic nature of polyurethane makes it challenging to elucidate the structural basis of enzyme–plastic interactions. Here, we used a fragment-based approach to characterize the active sites of two novel urethanases called amidase U11 and esterase AX^PUR5^ with different catalytic scaffolds, employing a crystallographic fragment-screening campaign to map subpockets in the active site and validate these subpockets as plastic binding sites by using soluble plastic fragment analogues that mimic the substrate, transition state or product. We resolved 42 high-resolution structures for both esterase AX^PUR5^ and amidase U11. The fragment-based approach identified new substrate-binding subpockets, while interactions of plastic mimetics in the active site provided a mechanistic understanding of the recognition and binding of polyurethane fragments by these subpockets. These results highlight a diversity of binding modes among urethanases towards different polyurethane fragments.

## Abstract 2

Fragment-based active-site exploration of urethanases was performed to elucidate polyurethane binding and cleavage.

## 1. Introduction

**Figure 1:**
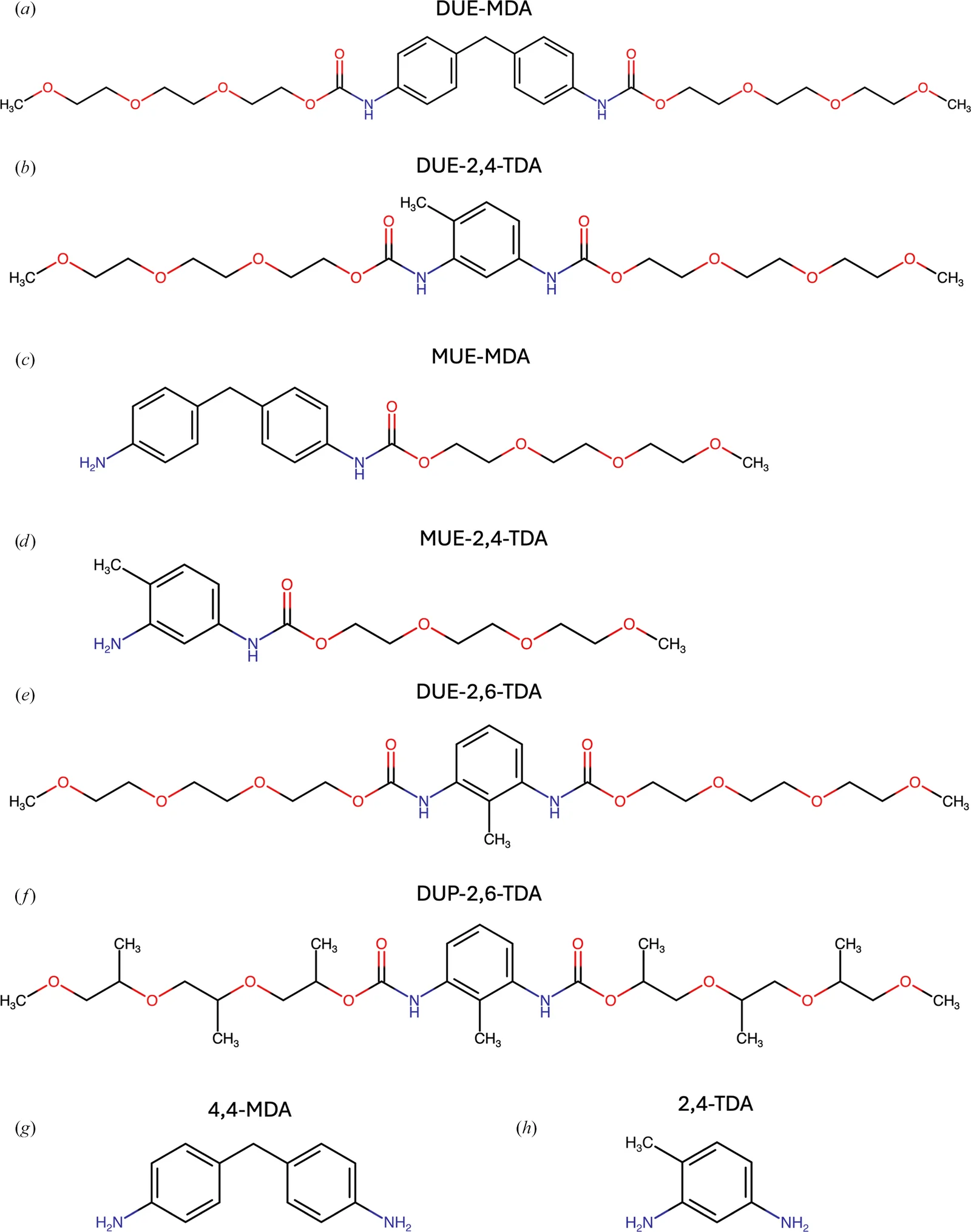
Structures of PU fragments. Substrates: (*a*) DUE-MDA and (*b*) DUE-2,4-TDA. Single urethane bond-degraded intermediate products: (*c*) MUE-MDA, (*d*) MUE-2,4-TDA, (*e*) DUE-2,6-TDA, (*f*) DUP-2,6-TDA. Double urethane bond-cleaved products: (*g*) 4,4-MDA, (*h*) 2,4-TDA. DUE, diurethane ethylene glycol; MUE, monourethane ethylene glycol; DUP, diurethane polypropylene glycol; MDA; methylene dianiline; TDA, toluenediamine. This figure was made with *ChemAxon*.

Polyurethane (PU) plastics are an essential part of modern society, forming numerous everyday materials due to their ease of manufacture, robustness and versatility (Rossignolo *et al.*, 2024 ▸). Despite these assets, the alarming accumulation of PU waste in the ecosystem, exceeding millions of tons, makes a pressing case for recycling strategies in the transition to a sustainable and circular economy (Banik *et al.*, 2023 ▸). Current methods for recycling PU are mostly based on expensive, open-loop and unsustainable mechano-chemical processing steps that generate byproducts, many of which are harmful to the environment, such as carbon dioxide (Polecki *et al.*, 2026 ▸). Biocatalytic, *i.e.* enzymatic, degradation provides an attractive alternative due to mild conditions and highly specific reactions. There is currently a rapid expansion in the discovery, characterization and engineering of polyurethane-degrading enzymes, or PURases, of typically microbial origin (Branson *et al.*, 2023 ▸; Chen *et al.*, 2025 ▸; Rotilio *et al.*, 2026 ▸; Bendtsen *et al.*, 2026 ▸), yet most of these novel PURases only present low-level activity on PU plastics, highlighting the urgent need for engineering campaigns to improve catalytic capacity and thermal stability. In this approach, atomic-level mechanistic understanding of enzyme–ligand interactions in the active site is a critical bottleneck. Commercial PU consists of repeating units of an aromatic isocyanate, commonly made from methylene dianiline (MDA) or toluenediamine (TDA), connected through a polyol chain (*e.g.* ethylene glycol) via a urethane bond (Fig. 1 ▸). Collectively, the aromatic group and the urethane bond constitute a rigid and hydrophobic segment. In contrast, the polyol chain forms a flexible and hydrophilic segment (Akindoyo *et al.*, 2016 ▸). The polymeric and highly insoluble nature of PU precludes atomic-level structural analysis of enzymes bound to *bona fide* substrates. A trick is to reduce the challenge by turning to either (i) fragments that mimics short segments of the aromatic moiety of PU or (ii) short and soluble PU analogues. This provides an alternative approach to explore both the catalytically active site as well as surrounding regions that take part in the binding and release of substrates and products, respectively.

Typical fragments for such studies generally follow the so-called ‘rule of three’, *i.e.* molecular weight <300 Da, <3 rotatable bonds and <3 hydrogen-bond donors/acceptors (Jhoti *et al.*, 2013 ▸). Due to their small size and simplicity, fragments effectively explore chemical space in the binding cavity of an enzyme. For example, in fragment-based drug-discovery campaigns, fragments usually serve as the starting point for structure-based drug design (Scott *et al.*, 2012 ▸). Moreover, fragments can be used to map the binding landscape of an enzyme’s extended active site by binding to all available pockets as potential ligand-binding sites. The identification of these binding sites can reveal the molecular basis of enzyme–substrate specificity and hence establish a rational basis for protein engineering, which is critical for most industrially relevant enzymes.

Most currently known urethanases fall into two different serine hydrolase families: (i) amidase signature (AS) family amidases (EC 3.5.1.4) and (ii) carboxylic acid esterases (EC 3.1.1.1). These two classes represent different catalytic scaffolds for PU degradation, potentially presenting distinct substrate binding and recognition modes. For this study, we selected two of our recently discovered novel PU-degrading enzymes, namely amidase U11, a member of the amidase signature family amidases discovered by database mining (Rotilio *et al.*, 2026 ▸), and esterase AX^PUR5^, a carboxylic esterase identified from plastic waste landfill (Bendtsen *et al.*, 2026 ▸). The two enzymes differ in their ability to degrade soluble PU model substrates such as DUE-MDA and DUE-TDA; both enzymes degrade the former at comparable levels, whereas the latter is degraded better by amidase U11.

Here, we employ a fragment-based approach to characterize the active-site cavity of PU-degrading enzymes, combining crystallographic fragment screening with binding analysis of PU fragment analogues. Our approach, called fragment-based active-site exploration (FASE), explores the extended active site, *i.e* both the catalytic site and neighbouring binding sites. We first performed a crystallographic fragment-screening campaign by using the FragMAX library (Lima *et al.*, 2020 ▸) to probe the active site of enzymes to map subpockets. Guided by these newly mapped pockets, we then analysed the binding of short, soluble plastic fragments that are analogues of the PU substrate, the transition state (TS) or the products of degradation (Fig. 1 ▸). The combination of various fragments maps the entire binding landscape for the active site of novel PU-degrading enzymes and uncovers specific binding poses for PU plastics. Additionally, through FASE, we perform a comparative characterization of the active sites of amidase U11 and esterase AX^PUR5^ to elucidate PU-binding modes and to explain the observed differences in activity across different PU substrates. These insights can guide discovery and engineering efforts of homologous enzymes within the same families and provide the molecular basis for the chemical promiscuity inherent to active sites in the AS and esterase family.

## 2. Materials and methods

### 2.1. Expression, purification and crystallization

#### 2.1.1. Protein expression

Genes encoding amidase U11/U11-S173A (UniProt/GenBank, *Sphingomonas spermidinifaciens*, NCBI reference code WP_096341946.1) and esterase AX^PUR5^ were cloned into pETM-11 and pET-28 plasmids, respectively, ensuring that each enzyme had a 6×His tag at the C-terminus preceded by a TEV cleavage site. The plasmids were transformed into *Escherichia coli* BL21(DE3) super-heat competent cells by heat shock. Briefly, plasmids were mixed with KCM buffer (0.2 *M* KCl, 0.2 *M* CaCl_2_, 0.2 *M* MgCl_2_) on ice for 5 min; 50 µl of competent cells were then added. After further incubation on ice for 20 min, cells were transferred to room temperature and incubated for a further 5 min. 400 µl LB medium at 37°C was added and the cells were incubated at 37°C in a shaking incubator at 150 rev min^−1^ for 1 h. 100 µl cell suspension was then dispersed onto a LB-agar plate containing 1 m*M* kanamycin and incubated at 37°C overnight, after which a single colony was transferred into 10 ml LB containing 1 m*M* kanamycin (50 µg ml^−1^). This starter culture was grown at 37°C overnight with shaking at 150 rev min^−1^ and then transferred to 1 l LB medium supplemented with 1 m*M* kanamycin and grown at 37°C at 150 rev min^−1^ in a shaker until the OD_600_ reached 0.6–0.8, cooled at 4°C for 30 min and induced with 0.5 m*M* isopropyl β-d-1-thiogalactopyranoside, after which the cells were grown at 18°C at 150 rev min^−1^ overnight. The cell pellets were harvested at 10 000*g* for 20 min and stored at −20°C until purification.

#### 2.1.2. Protein purification

The pellet was suspended in 50 ml lysis buffer (per litre of cell culture) consisting of 50 m*M* Tris–HCl pH 8.0, 500 m*M* NaCl, 25 m*M* imidazole pH 8.0, 1 m*M* DTT, 1 m*M* PMSF (PMSF was omitted for active protein purification), incubated on a rotating table at 60 rev min^−1^ at 4°C for 30 min and lysed by sonication for three cycles of 2 min at 30% power with 1 s pulses. The lysate was cleared by centrifugation for 45 min at 30 000*g*, after which the supernatant was filtered with a 0.45 µm membrane filter and loaded onto a HiTrap 5 ml Ni–NTA column previously washed with five column volumes of Milli-Q water and then equilibrated with five column volumes of binding buffer (50 m*M* Tris–HCl pH 8.0, 500 m*M* NaCl, 25 m*M* imidazole pH 8.0) before loading. Protein binding to the column took place for up to two hours to maximize binding. His-tagged proteins were eluted with elution buffer (50 m*M* Tris–HCl pH 8.0, 500 m*M* NaCl, 500 m*M* imidazole pH 8.0) in stepwise concentration gradients of 10%, 25%, 50% and 100% elution buffer. Fractions were run on SDS–PAGE to check purity. To remove the His-tag, fractions with enzymes were dialysed overnight against SEC buffer (50 m*M* Tris–HCl pH 8.0, 150 m*M* NaCl) containing TEV protease in 10 kDa cutoff dialysis-bag membrane at 4°C with 1 mg TEV protease per 10 mg of enzyme, concentrated in a Vivaspin concentrator with 10 kDa cutoff and loaded onto a Superdex 200 10/300 GL column. Peak fractions were analysed by SDS–PAGE and concentrated with a 10 kDa cutoff to 20 and 40 mg ml^−1^ for amidase U11 and esterase AX^PUR5^, respectively (concentrations were determined by OD_280_ using extinction coefficients of 51 700 *M*^−1^ cm^−1^ for U11 and 84 130 *M*^−1^ cm^−1^ for AX^PUR5^.

#### 2.1.3. Protein crystallization

Initial crystallization screening plates were set up in SWISS 3-well plates with different protein:reservoir ratios (1:1, 2:1 and 1:2) at a final drop size of 600 nl and kept at 19°C. Initial hits for amidase U11 were found in 20%(*w*/*v*) PEG 3350, 0.2 *M* magnesium acetate tetrahydrate and for esterase AX^PUR5^ in 20%(*w*/*v*) PEG 3350, 0.2 *M* calcium acetate. Gradient optimization of the initial concentrations of salt, precipitants and protein was conducted in 24-well plates alongside seeding with different dilutions of the initial seed stock. Final optimized crystallization conditions were found as follow: for amidase U11, 20%(*w*/*v*) PEG 3350, 0.2 *M* magnesium acetate mixed with 18 mg ml^−1^ amidase U11 in a 2:1(*v*:*v*) drop ratio; for esterase AX^PUR5^, 20%(*w*/*v*) PEG 3350, 0.15 *M* calcium acetate mixed with 28 mg ml^−1^ esterase AX^PUR5^ in a 1:1(*v*:*v*) drop ratio.

### 2.2. Fragment soaking and crystal mounting

Plastic fragments including DUE-MDA, MUE-MDA, DUE-2,4-TDA and MUE-2,4-TDA were synthesized as a powder or oil as described elsewhere (Bendtsen *et al.*, 2026 ▸), and the synthesis of the transition-state analogues DUE-2,6-TDA and DUP-2,6-TDA is described in detail in the supporting information. These fragments were solubilized in 100% DMSO by vortexing several times to a final concentration of 1 *M*. For soaking, 1.0–1.5 µl stock fragment solution was directly added into a 6 µl crystal drop with a final concentration of compounds of around 100 m*M*. Fragment soaking was performed from 10 min to 24 h. Crystals were then briefly soaked in 20–25% glycerol mixed with mother liquor, fished out using a nylon loop (Hampton Research) or LithoLoop (Molecular Dimensions) and flash-cooled in liquid nitrogen. Compounds from the FragMAX fragment library (Lima *et al.*, 2020 ▸) were solubilized in mother liquor to a final DMSO concentration of 10% and briefly spun to remove air bubbles, after which 1–1.2 µl fragment buffer was directly added to the crystal drops using a multichannel pipette. For fishing and mounting fragments, the Easy Access Frame (Barthel *et al.*, 2021 ▸) was used to prevent evaporation.

### 2.3. X-ray diffraction data collection, processing and structure solution

Initial crystal data collections were performed on the P13 and P14 beamlines at DESY, Hamburg and the I24 beamline at Diamond Light Source. Final X-ray diffraction data were collected on the BioMAX beamline at MAX IV, Lund, Sweden. All data sets were integrated and scaled with *XDS* (Kabsch, 2010 ▸). *POINTLESS* (Evans, 2011 ▸) was used to assign correct space groups. If auto-processed data from the *autoPROC* pipeline (Vonrhein *et al.*, 2011 ▸) had reasonable statistics for the high-resolution shell, they were used in the subsequent stages of structure determination, typically with a CC_1/2_ score above 0.3 used for the resolution cutoff. Initial templates for molecular replacement were generated by *AlphaFold*3 (Abramson *et al.*, 2024 ▸). Before molecular replacement, data sets were analysed using *phenix.xtriage* (Adams *et al.*, 2010 ▸) and *CTRUNCATE* (Evans, 2011 ▸) to check for potential twinning or pseudo-translational issues. Molecular replacement was performed by *Phaser* (McCoy *et al.*, 2007 ▸). Refinement and final manual model building were performed primarily using *phenix.refine* (Afonine *et al.*, 2012 ▸) and *Coot* (Emsley *et al.*, 2010 ▸), respectively. Additionally, *REFMAC*5 (Murshudov *et al.*, 2011 ▸) was used in some cases to compare the final *R* factor obtained with *phenix.refine* in problematic cases (for example fragment-bound structures or high-*R*_free_ cases). Ligand restraints were obtained using *AceDRG* (Long *et al.*, 2017 ▸) and *Elbow* (Moriarty *et al.*, 2009 ▸). Structure visualization and figure preparation were performed in *PyMOL* (Schrödinger).

### 2.4. HPLC analysis of PU fragment hydrolysis

Endpoint samples were collected after the incubation of 500 n*M* enzyme with 0.2 mg ml^−1^ DUE-TDA or DUE-MDA for 3 h at 40°C in 100 m*M* bis-tris propane pH 9.0, 100 m*M* NaCl. The reaction was stopped by adding 100% acetonitrile at a 1:1 ratio, after which the aliquots were filtered through a 0.22 µm PTFE filter. All reactions were performed in duplicate. HPLC was used to quantify the production of monosubstituted monourethane ethylene (MUE)-MDA and MUE-TDA, and their breakdown products 4,4-MDA and 2,4-TDA, respectively, using a Shimadzu system and UV absorbance at 240 nm. 10 µl of sample was injected onto a Zorbax Eclipse Plus C18 column with 5 µm particle size at 40°C and a flow rate of 1 ml min^−1^. The mobile phase consisted of (*A*) Milli-Q water and (*B*) 100% acetonitrile. For DUE-TDA, a gradient separation from 30–80% *B* for 6 min was used, while DUE-MDA separation involved a 5 min isocratic elution (50% *B*). Standard curves for DUE-MDA, MUE-MDA, 4,4-MDA, DUE-TDA, MUE-TDA and 2,4-TDA were used to assign peaks and convert areas to concentrations. All standard curves could be described by linear regression with *R*^2^ ≥ 0.95.

## 3. Results

### 3.1. Structural characterization of the amidase and esterase

The determination of key positions and cavities for the catalytic properties of the enzymes requires comparison of the apo state with the complexed state. Therefore, we first determined the structure of the two enzymes amidase U11 (PDB entry 30sd) and esterase AX^PUR5^ (PDB entry 29jv) in the ligand-free state and then in the presence of a general suicide inhibitor, the latter helping to define the catalytic scaffold and the active site.

#### 3.1.1. Structural characterization of the amidase with and without a small suicide inhibitor reveals the overall fold and catalytic pocket

**Figure 2:**
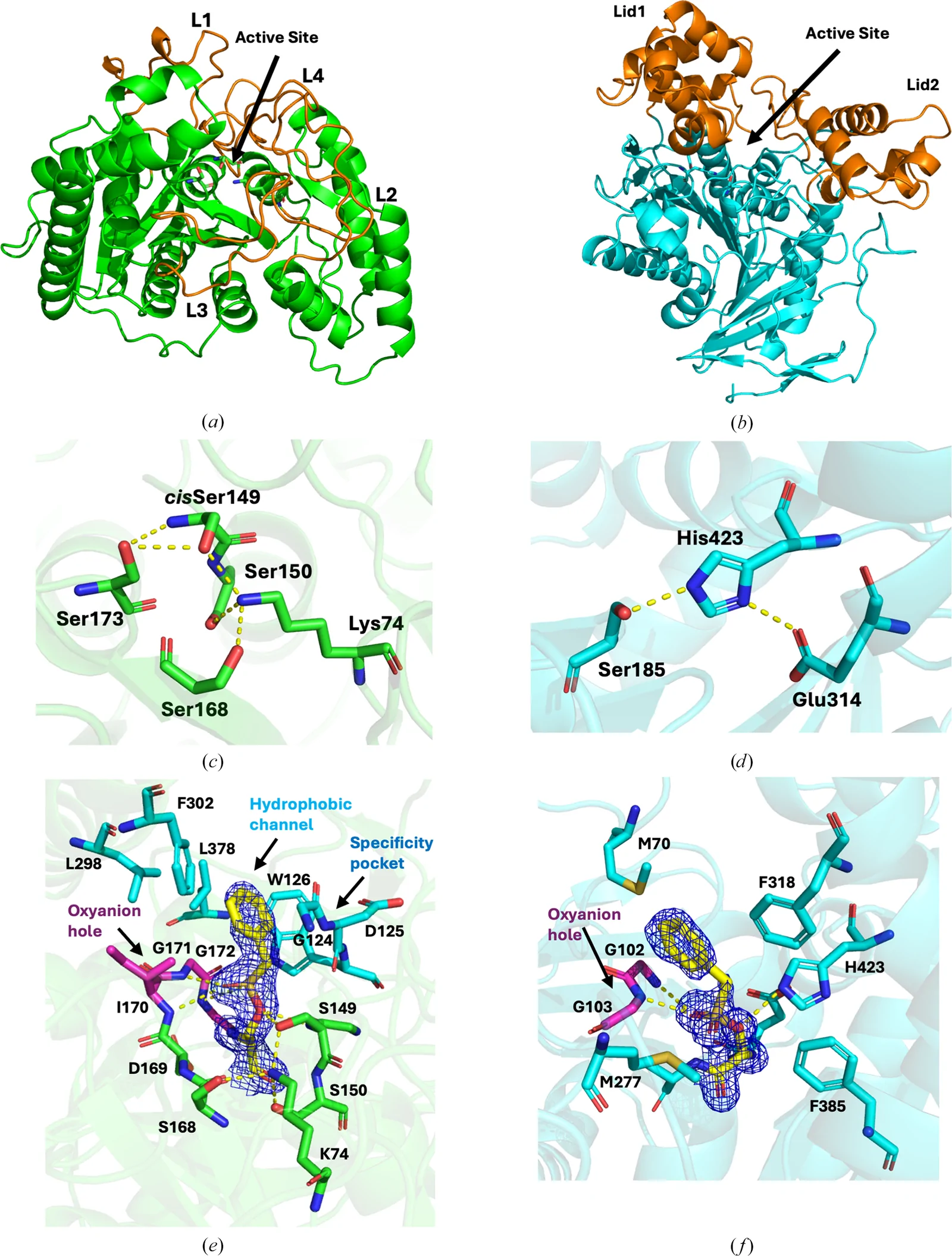
Structural overview of two different catalytic scaffolds for polyurethane degradation. (*a*) Overall structure of amidase U11 (PDB entry 30sd) with the catalytic triad depicted as sticks and L1–L4 shown in orange. (*b*) Overall structure of esterase AX^PUR5^ (PDB entry 29jv) shown with the catalytic triad residues as sticks and the Lid1 and Lid2 domains shown in orange. (*c*, *d*) The catalytic triad (Ser173, *cis*Ser149 and Lys79) along with two conserved serine residues that maintain the hydrogen-bonding network of amidase U11 (*c*) and the catalytic triad of AX^PUR5^ (*d*). (*e*, *f*) Structures of the PMS-bound region in amidase U11 (PDB entry 30fo) (*e*) and esterase AX^PUR5^ (PDB entry 29jy) (*f*) with surrounding residues shown as sticks. The 2*mF*_o_ − *DF*_c_ standard map at a 1.5σ contour level for PMS is depicted as a blue mesh.

The asymmetric unit in space group *P*6_1_22 consists of a dimer of amidase U11. A twofold noncrystallographic symmetry axis relates the two monomers to the *b* axis. In addition, we performed size-exclusion chromatography to confirm a dimeric assembly in solution (Supplementary Fig. S1*b*). The overall structure of the monomer consists of 12 α-helices (αA–αL) and ten β-strands (β1–β10) forming a twisted mixed β-sheet surrounded by α-helices on both sides, similar to an α–β sandwich topology (Fig. 2 ▸*a*). The active-site cavity is formed by four long loops (L1, 73–100; L2, 118–150; L3, 188–211; L4, 348–375) and two α-helices (αI and αK) on one edge of the cavity. A noncrystallographic twofold symmetry axis is established by direct contact between the αI and αJ helices, and the same two helices also form a channel connected to the catalytic pocket. The catalytic triad, composed of the nucleophilic Ser173, *cis*Ser149 and Lys74, is located deep in the pocket and closer to αI and αK. The correct alignment of 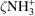 of Lys74 in the catalytic triad is maintained by hydrogen bonding from Ser168, Ser150 and *cis*Ser149 (Fig. 2 ▸*c*). Moreover, *cis*Ser149 also establishes two direct hydrogen bonds to the nucleophilic serine via the backbone amino group and O^γ^ due to the unusual *cis* configuration (Fig. 2 ▸*c*). A *DALI* search (Holm *et al.*, 2023 ▸) found UMG-SP1-3, one of the first reported PU-degrading AS amidases (Branson *et al.*, 2023 ▸), to be the closest structural homologue to U11, as was expected since U11 was found through a homology search to UMG-SP1 (Rotilio *et al.*, 2026 ▸). UMG-SPs share a very similar overall fold (*i.e.* C^α^ r.m.s.d. < 1.0 Å) and active site with U11, which indicates a highly conserved evolutionary catalytic scaffold. In summary, U11 represents a typical AS amidase catalytic scaffold that is highly conserved across bacteria, archaea and eukaryotes (Chebrou *et al.*, 1996 ▸).

To understand transition-state stabilization and possible substrate-recognition mechanisms, amidase U11 was purified and crystallized in the presence of phenylmethylsulfonyl fluoride (PMSF), a catalytic mechanism-based suicide inhibitor commonly used against serine proteases. In the crystal structure, phenylmethylsulfonyl (also known as the PMSF adduct) forms a covalent bond to the nucleophilic Ser173 (Fig. 2 ▸*e*), and a complete electron-density map for PMS is available at 1.80 Å resolution. The sulfonyl group of PMS mimics an *sp*^3^-hybridized tetrahedral transition state. It is stabilized by the oxyanion hole formed by Gly171 and Gly172, the backbone amine groups of which contact the O atoms of the sulfonyl group. The benzyl group of PMS fits into the pocket formed by Trp126, Asp125 and Gly124, in which Trp126 stabilizes a T-shaped π–π stacking interaction with the benzylic ring of PMS. Overall, this pocket resembles the specificity pockets also observed in several serine proteases that drive substrate selectivity (Hedstrom, 2002 ▸) and have previously been described for signature amidases (Rotilio *et al.*, 2025 ▸). Moreover, a hydrophobic channel formed by Leu298, Phe302 and Leu378 directly connects the bulk solvent to the catalytic pocket, constituting a possible route for substrate diffusion into the active site.

#### 3.1.2. Structural characterization of the esterase identified a TEV peptide ligand bound to the active site

The second enzyme, esterase AX^PUR5^, is structurally and evolutionarily different from U11. AX^PUR5^ crystallized in space group *P*2_1_2_1_2_1_, with observed diffraction spots up to 1.0 Å resolution, revealing an asymmetric unit comprising a single polypeptide chain. The structure was solved at 1.29 Å resolution. Yet, gel-filtration analysis suggested a dimeric state in solution (Supplementary Fig. S1*a*). The monomeric structure of esterase AX^PUR5^ is a centrally located α/β-hydrolase fold, with two α-helical lid domains atop the catalytic core (Fig. 2 ▸*b*). The active site is a deep cavity located between the two lid domains. It exhibits a highly hydrophobic character, surrounded by several aromatic residues, with the catalytic triad located at the centre (Fig. 2 ▸*d*). The nucleophilic Ser185, His423 and Glu314 comprise the catalytic triad, which is very similar to that of serine proteases (Di Cera, 2009 ▸). The catalytic serine is located on the nucleophilic elbow, as seen in numerous other carboxylic esterases and α/β-hydrolases in general (Heikinheimo *et al.*, 1999 ▸). In the ligand-free structure, the catalytic triad residues are connected by a hydrogen-bonding network that stabilizes the catalytically active conformation. Strikingly, the ligand-free structure of AX^PUR5^ contains an unexpected peptide ligand captured between the two lid domains (Supplementary Fig. S2*a*), the sequence of which we can assign thanks to the near-atomic resolution structure of the esterase, revealing leucine, tyrosine and phenylalanine residues, as well as backbone density for other side chains. The LYF sequence is found in the TEV protease recognition sequence (–ENLYFQ–) used for His-tag cleavage in this construct, while residual density consistent with Asn and Gln side chains further supports our hypothesis. Further refinement of the NLYFQ ligand yielded full density that clearly followed the peptide at a low contour level (Supplementary Fig. S2*a*), with a very strong omit map (Supplementary Table S2). The Tyr and Phe side-chain residues are inserted into a hydrophobic pocket lined by several aromatic residues. At the same time, the remaining part of the peptide is more solvent-exposed due to polar side chains. To our knowledge, this is the only structure in the Protein Data Bank (PDB) in which the TEV recognition peptide is bound to an enzyme other than TEV protease. Even though the peptide does not directly mimic plastic fragments, strong similarity to PU fragments in terms of size, bulkiness and hydrophobicity implies the probable binding of bulky plastic fragments at the same site.

To identify catalytically active residues, we performed the purification in the presence of PMSF, a broad-spectrum serine hydrolase inhibitor. Indeed, in the resulting complex, the PMSF adduct is covalently linked to the catalytic Ser185. At the same time, the sulfonyl oxygen interacts with backbone amine groups of the oxyanion hole (Gly102 and Gly103) via hydrogen bonding (Fig. 2 ▸*f*). Upon PMSF binding, the TEV peptide that we observed in the ligand-free structure cannot bind to a similar site because of a steric clash with the benzylic rings of the PMSF adduct and phenylalanine from the TEV peptide. There is no specific recognition pocket near the benzylic ring in esterase AX^PUR5^, in contrast to amidase U11. Instead, the benzylic ring of the PMSF adduct is fitted into the large hydrophobic pocket surrounded by aliphatic and aromatic side-chain residues. The active site of the esterase is quite large and hydrophobic, allowing considerable substrate promiscuity.

### 3.2. Fragment-based active-site probing maps potential subpockets in both enzymes

**Figure 3:**
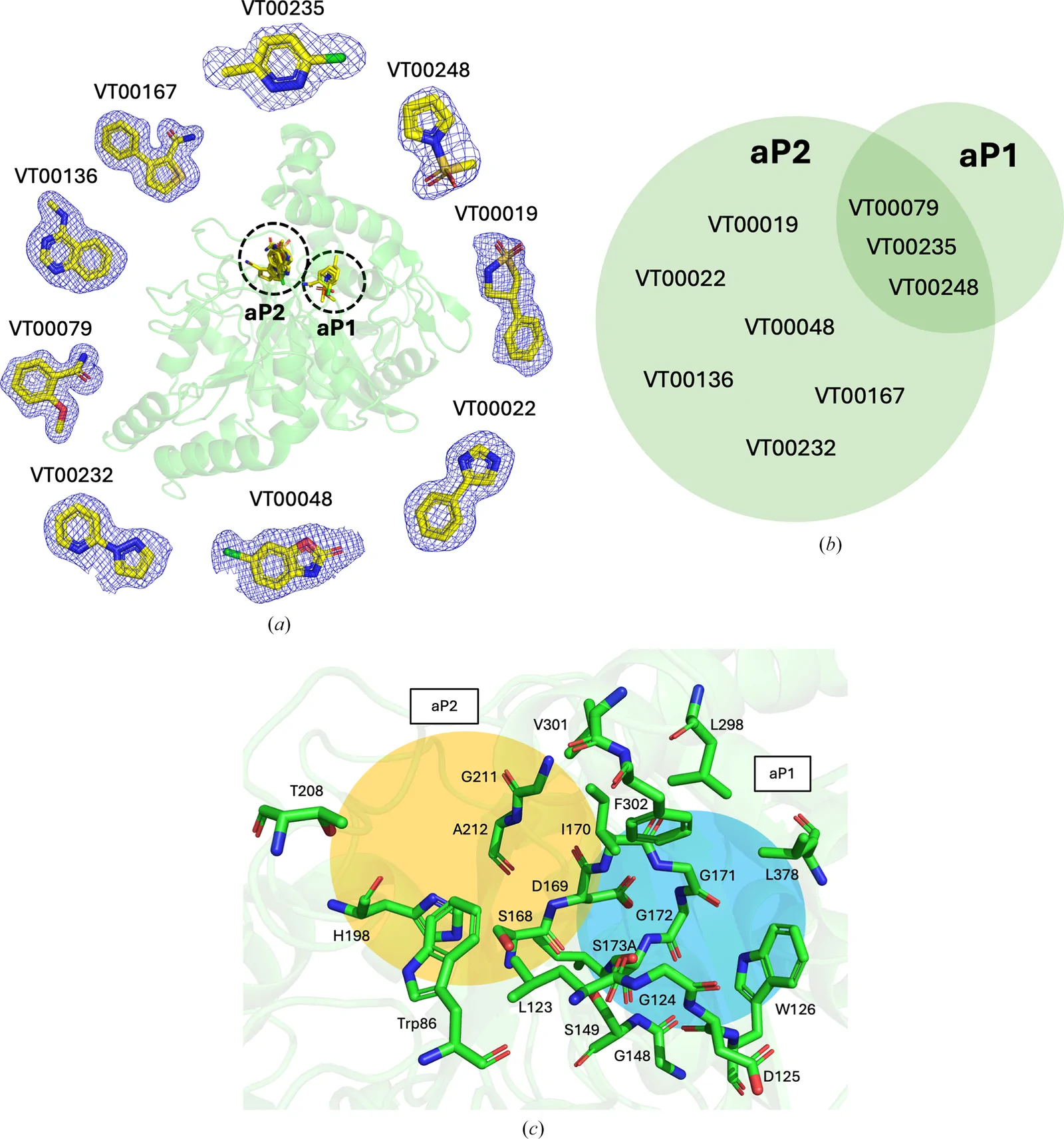
Fragment-based active-site mapping/probing identifies potential pockets for plastic binding to amidase U11-S173A. (*a*) Superimposed structures of fragments for amidase with each ligand demonstrated separately. The 2*mF*_o_ − *DF*_c_ standard map at a 1.5σ contour level for amidase fragments is shown as a blue mesh. (*b*) Overlap of fragments that bound to aP1 (three hits) and aP2 (nine hits) shown in circles with relative sizes corresponding to the number of hits. (*c*) Close-up view of fragment-binding pockets with surrounding residues highlighted; the pockets are shown in shaded colours.

PMSF adduct-bound structures of amidase U11 and esterase AX^PUR5^ enabled characterization of the catalytic pocket, highlighting the oxyanion hole and suggesting potential mechanisms for substrate recognition. Nevertheless, the existence of additional hydrophobic pockets distant from the catalytic pocket and potentially related to the binding of bulkier plastic fragments remains unclear. Therefore, we implemented a crystallographic fragment-screening campaign to probe the active-site cavity and map additional binding sites beyond the catalytic pocket. We used the FragMAX library (Lima *et al.*, 2020 ▸), which consists of 160 high-concentration, high-solubility, nonredundant small molecules, on the amidase and esterase crystals. As a result of the fragment-screening campaign, we identified nine hits clustering in two pockets for the amidase (aP1 and aP2) and 12 hits clustering in three pockets for the esterase (eP1, eP2 and eP3), with complete electron density for fragments in standard maps (Figs. 3 ▸*a* and 4 ▸*a*) and very strong omit maps for most of the fragments (Supplementary Figs. S3 and S4). Unfortunately, there is very little overlap in fragment binding between the two enzymes. Only one fragment (VT00167) is bound to both the esterase and amidase (Supplementary Fig. S5). An issue is that during soaking and data collection, many fragments induce crystal damage, which results in low or no diffraction. This prevents a systematic comparison of binding preferences for individual fragments to esterase and amidase.

#### 3.2.1. Amidase pocket mapping identifies two subpockets for potential PU binding

We identify two pockets in the amidase active site where aromatic fragment groups are clustered (Fig. 3 ▸*c*). A summary of the interactions of individual fragments with aP1 and aP2 is shown in Table 1 ▸. The first pocket (aP1) correlates with the catalytic pocket since it is the same place where PMS is covalently bounded to the catalytic Ser173 and establishes interactions with the oxyanion hole (Ile170, Gly171 and Gly172), catalytic pentad (Lys74, *cis*Ser149, Ser150, Ser168 and Ser173) and substrate-recognition pocket (Gly124, Asp125 and Trp126) (Fig. 3 ▸*c*). Compounds VT00079, VT00235 and VT00248 bind to this site with a similar orientation of aromatic groups towards the specificity pocket (Fig. 2 ▸*e*). Fragment VT00079 bound to this pocket makes aromatic π–π interactions with Trp126 via its aromatic group, potentially constituting the molecular basis for substrate selectivity and recognition. Additionally, oxyanion-hole backbone groups make hydrogen bonds with fragments if there is any donor/acceptor group available (Table 1 ▸), similar to the sulfonyl group interaction of PMS, which encapsulates TS stabilization. aP1 is connected to the bulk solvent via a large hydrophobic channel at the top of the pocket that is surrounded by Leu298, Phe302 and Leu378 (Fig. 3 ▸*c*). Thus, it is highly probable that there are weak interactions of fragments in aP1 due to the solvent-exposed channel and weak hydrophobic interactions. Indeed, the hydrophobic channel that connects aP1 to the bulk solvent (Fig. 2 ▸*e*) might serve as a substrate/product binding/release route.

The second pocket (aP2) is located close to aP1 and is buried in the active site by Loop2 and Loop3. Notably, this pocket is deep, large, highly hydrophobic and partially protected from the bulk solvent, in clear contrast to aP1 (Fig. 3 ▸*c*). All fragments bind to aP2 (Fig. 3 ▸*b*), indicating ligand promiscuity of this site compared with aP1. aP2 is surrounded by Trp86, Leu123, Ser168, Ile170, His198, Gly211, Ala212 and Phe302. The major binding environment for ligands consists of hydrophobic interactions in addition to a few hydrogen-bonding and π–π interactions via Thr208 and Trp86, respectively. Aligned structures of the ligand-free structure and fragment-bound complexes indicate no significant conformational change in the surrounding residues, implying a lock-and-key-based enzyme–ligand interaction due to the large space available for bulky fragment binding (Supplementary Fig. S6). Five- and six-membered aromatic ring groups of fragments occupy a similar position in the relatively large hydrophobic cavity of aP2 (Fig. 3 ▸*a*). In summary, aP2 generally binds more bulky and hydrophobic ligands compared with the fragment found in aP1, which is a more polar and exposed pocket with access to the solvent channel (Fig. 2 ▸*e*). Indeed, fragments with a single aromatic ring structure (*i.e.* VT00079, VT00235 and VT00248) can bind to aP1, whereas fragments with more than one ring structure cannot probe aP1 due to bulkiness and enhanced hydrophobicity (Fig. 3 ▸*a* and Table 1 ▸). This observation again supports the potentially important role of the aP2 site in binding more hydrophobic and bulky fragments that mimic PU aromatic groups.

#### 3.2.2. Esterase pocket mapping identifies three potential subpockets for PU binding

**Figure 4:**
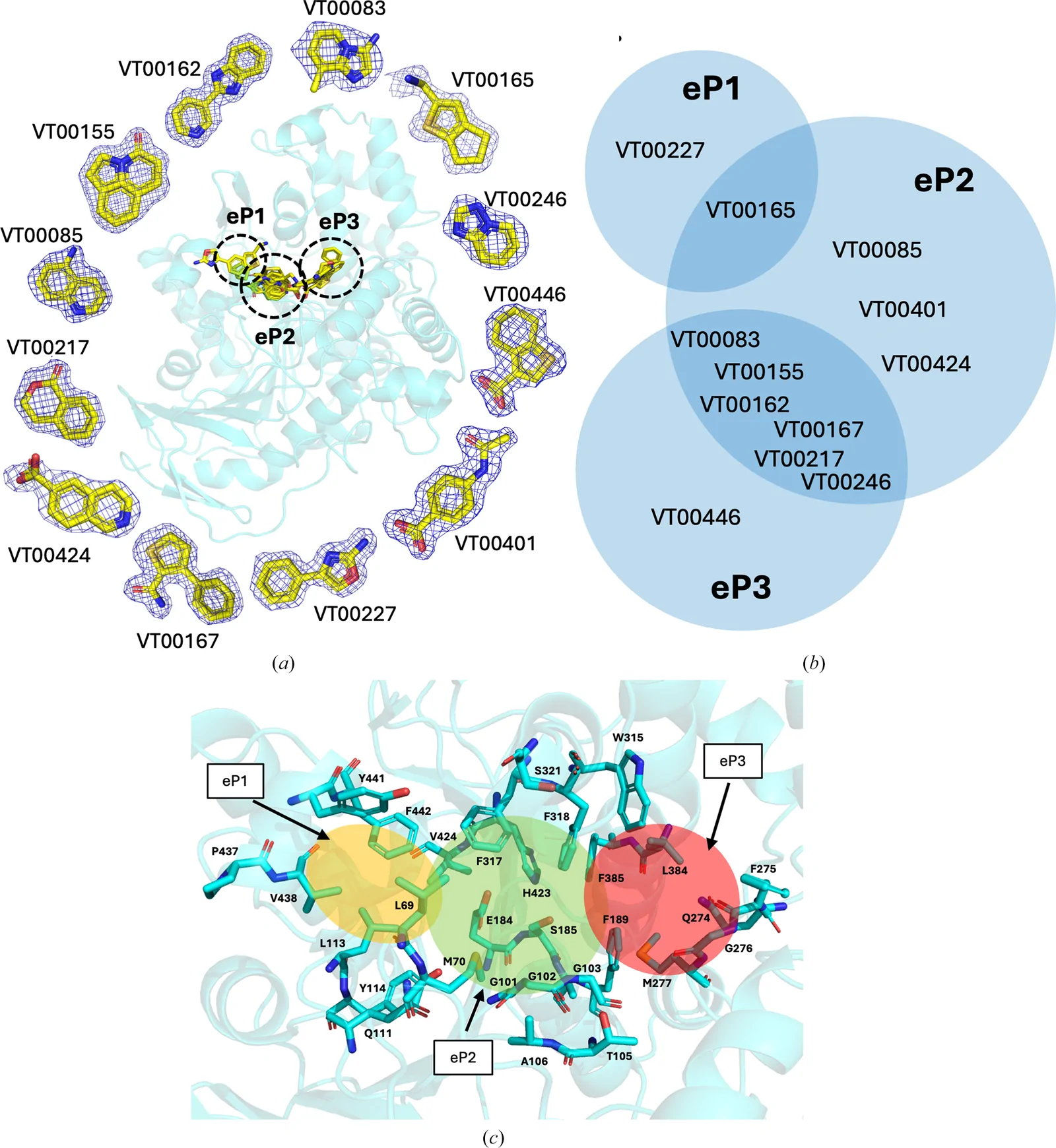
Fragment-based active-site mapping/probing identifies potential pockets for plastic binding to esterase AX^PUR5^. (*a*) Superimposed structures of fragments for esterase with each ligand demonstrated separately. The 2*mF*_o_ − *DF*_c_ standard map at a 1.0σ contour level for esterase fragments is shown as a blue mesh. (*b*) The overlap of fragments that bound to eP1 (two hits), eP2 (ten hits) and eP3 (seven hits) is shown in circles with relative sizes corresponding to the number of hits. (*c*) A close-up view of the fragment-binding pockets with surrounding residues highlighted and the pockets shown in shaded colours.

The fragment-based screening revealed more hits in esterase AX^PUR5^ (12/160) than in amidase U11, which we attribute to the larger and more solvent-accessible active site in the esterase. Three non-overlapping pockets (eP1, eP2 and eP3) are identified in the large active-site cavity (Fig. 4 ▸*a*). Table 2 ▸ summarizes the fragments that bound to the corresponding pockets and the key interactions involved in those pockets. eP1 is located at the exit site of the large cavity, surrounded by Pro437, Val438, Tyr441 and Phe442, and binds two fragments (VT00165 and VT00227). Most of the fragments bind to eP2, which surrounds the nucleophilic Ser185. Specifically, fragments mostly interact with Ser185, the oxy­anion hole (Gly102 and Gly103) and surrounding hydrophobic residues. Notably, fragments with hydrogen-bond donor/acceptor groups (*i.e.* VT00083, VT00085, VT00401 and VT00424) are oriented in a favourable conformation to establish hydrogen bonds with the side chains of Gln111, Tyr114, Glu184 and Ser185, as well as backbone groups in the oxyanion hole (Gly102 and Gly103) (Table 2 ▸), thus mimicking possible interactions with the substrate and the leaving group (either amine or carboxyl) after urethane bond degradation.

eP3 is located slightly above eP2 and is buried deep within the hydrophobic cavity, partially overlapping the peptide-binding site (Fig. 4 ▸*b*). eP3 is surrounded by Phe189, Met277, Val384 and Phe385. Aromatic groups of the binding fragments (*i.e.* VT00162 and VT00167) partially overlap in this pocket, forming mostly hydrophobic interactions and a few favourable π–π stacking interactions with Trp315, as well as hydrogen-bonding interactions with Gly102, Gly103, Ser185 and Gln274 (Table 2 ▸). As observed in the ligand-free structure, the same site is protected from the solvent by a trapped TEV peptide fragment, in which Phe and Tyr rings of the TEV peptide extend towards this extremely hydrophobic pocket and might prevent the influx of an entropically unfavourable ordered solvent network.

All in all, the active-site cavity of the esterase AX^PUR5^ is highly hydrophobic and large, allowing the binding of fragments that are bulkier and more hydrophobic compared with the amidase. Moreover, it is surrounded by several aromatic and aliphatic side chains that contribute to binding, as well as a few strategic polar side chains that can form hydrogen bonds and enhance specificity for the ligand. However, hydrophobic interactions provide the major force stabilizing fragment binding (Table 2 ▸), again highlighting the hydrophobic nature of the active site of esterase AX^PUR5^, appropriate for PU fragments. eP1 is targeted less by fragments and is more polar, whereas eP2 and eP3 are densely populated by hydrophobic side chains, which implies a potential role in binding aromatic groups of PU. Indeed, most fragments bind to eP2 and eP3 due to the larger, more hydrophobic space available to them, in contrast to the more solvent-exposed eP1. For example, bulky fragments such as VT00155 and VT00162 bind to both eP2 and eP3 but not to eP1 (Fig. 4 ▸*a* and Table 2 ▸). Six fragments bind to both eP2 and eP3, whereas one fragment (VT00446) is unique to eP3 and three fragments (VT00085, VT00401 and VT00424) are unique to eP2 (Fig. 4 ▸*b*). This differential binding can be explained by two effects: (i) a preference for hydrogen bonding to residues close to the given pocket and (ii) differences in the local structure of fragments, especially branching of side chains from ring structures, which causes the fit of fragments in different pockets depending on the local stereochemistry (Fig. 4 ▸*c* and Table 2 ▸). In summary, the polar groups in eP1 and eP2 may help orient polar groups in PU and favour catalytically productive conformations, while eP2 and eP3 may bind bulky and hydrophobic ligands such as PU.

### 3.3. Validation of subpockets as PU fragment-binding sites

**Figure 5:**
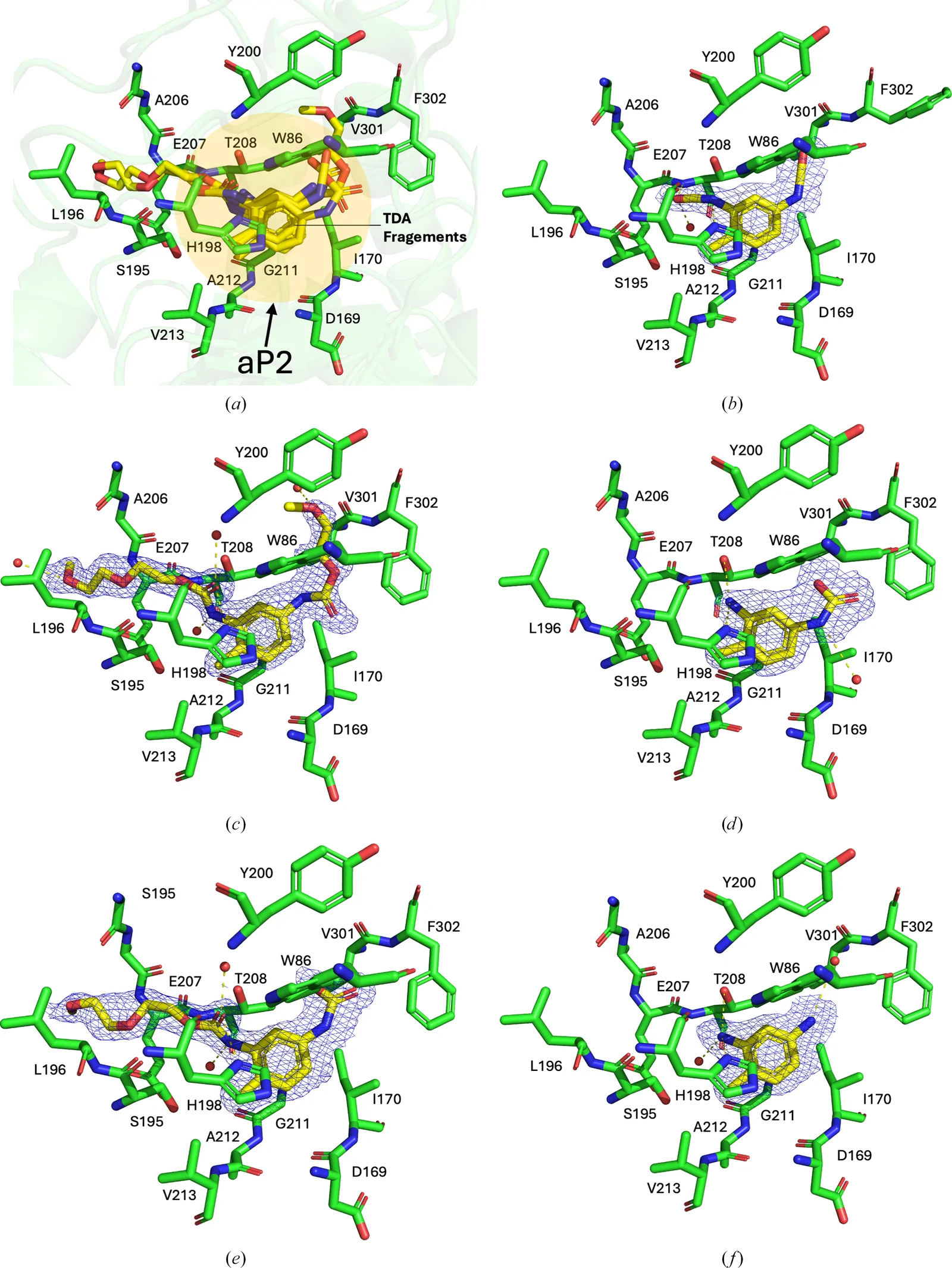
Validation of subpockets as plastic fragment-binding sites in amidase U11. (*a*) Superimposed structures of bound fragments in aP2 and surrounding residues in the pocket are shown. (*b*) 2,4-TDI (PDB entry 30qw). (*c*) DUE-2,4-TDA (PDB entry 30hf). (*d*) 2,4-TDA (carbamic acid; PDB entry 30hg). (*e*) MUE-2,4-TDA (carbamic acid; PDB entry 30nq). (*f*) 2,4-TDA (amine; PDB entry 30rg). 2*mF*_o_ − *DF*_c_ maps at a 1.0σ contour level are shown as a blue mesh for the ligand along with surrounding residues. Hydrogen bonds are shown as dashed yellow lines and water molecules are depicted as red spheres.

**Figure 6:**
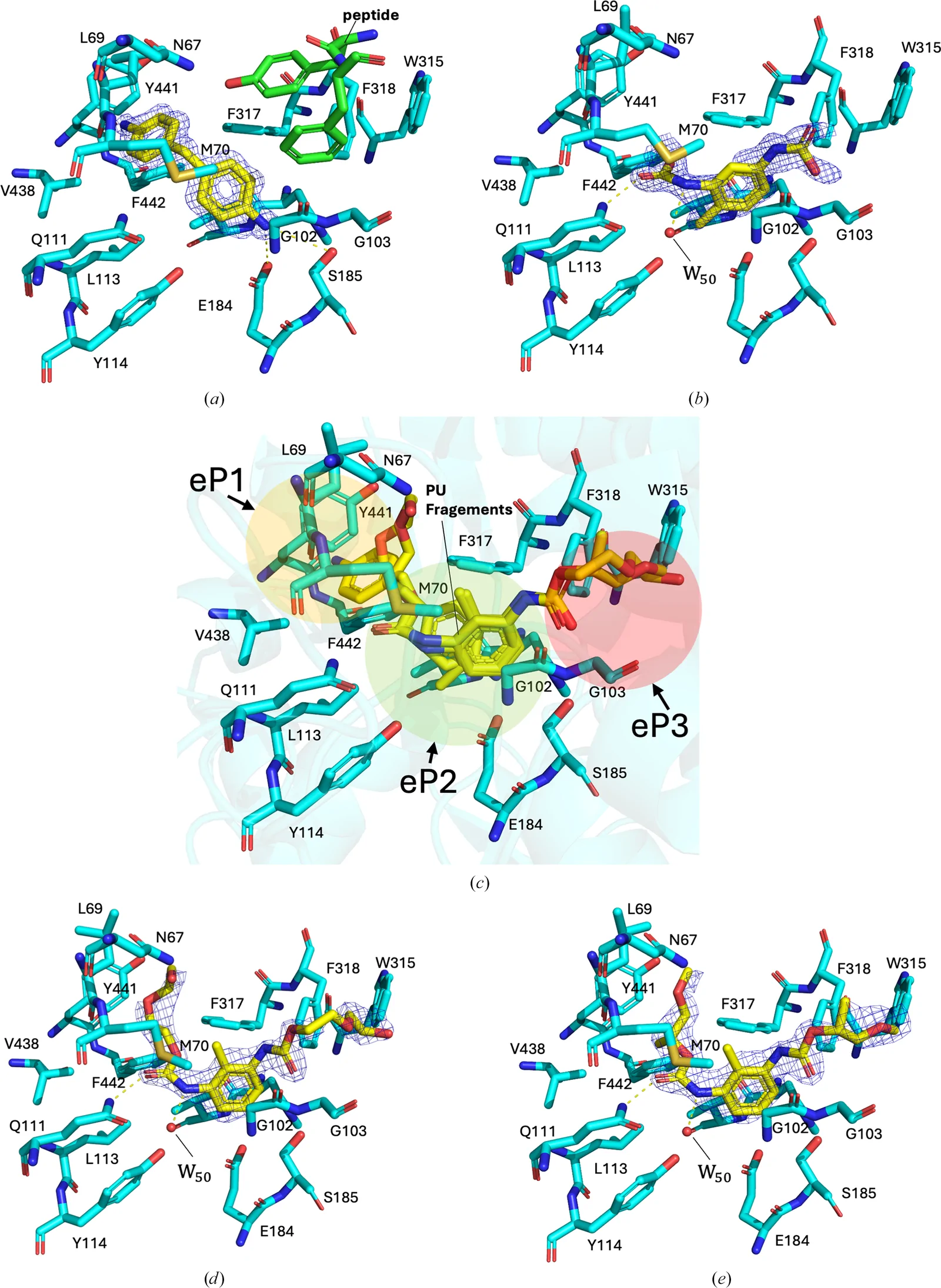
Validation of subpockets as plastic fragment-binding sites in esterase AX^PUR5^. (*a*) 4,4-MDA-bound structure (PDB entry 29kx) in the presence of trapped peptide (green) or (*b*) 2,4-TDA degradation product (carbamate; PDB entry 29nv). (*c*) Superimposed structure of bound fragments in eP1, eP2 and eP3 with surrounding residues are shown: (*d*) 2,6-TDA (ethylene glycol; PDB entry 29oj), (*e*) 2,6-TDA (polypropylene; PDB entry 29oo). The 2*mF*_o_ − *DF*_c_ map at a 1.0σ contour level is shown as a blue mesh for fragments. Hydrogen bonds are shown as dashed yellow lines and water molecules are depicted as red spheres.

The fragment-based pocket identification approach provides two and three pockets as potential binding sites for PU fragments in the amidase and esterase, respectively. To test whether any of these mapped pockets in esterase AX^PUR5^ and amidase U11 participate in the binding of bulky PU fragments, we used several PU analogues as TDA- and MDA-based substrates, intermediates and product analogues. The main problem with hydrophobic plastic fragment soaking is low solubility; many fragments precipitate when added to the crystal drop. We found it necessary to solubilize the fragments at high concentration (100 m*M*) in 100% DMSO stock to maximize solubility to saturate binding sites for low-affinity PU fragments for at least 2 h and up to >20 h. This led to five different TDA-based polyurethane fragments bound to U11, in which the aromatic groups of all TDA fragments bind to aP2 (Fig. 5 ▸). For AX^PUR5^, we determined the structures of four plastic fragments, namely 4,4-MDA and DUE-TDA analogues, bound to eP1 and eP2 (Fig. 6 ▸).

#### 3.3.1. Amidase PU fragment-bound structures show a bulky TDA-based PU binding site at aP2

To obtain plastic fragments in the active-site cavity, the inactive amidase mutant S173A was expressed, purified and crystallized. For soaking experiments, various soluble plastic fragments, including DUE-MDA, DUE-2,4-TDA, MUE-MDA, MUE-2,4-TDA, 2,4-TDA, 2,4-TDI and 4,4-MDA, were soaked in both U11-WT and U11-S173A. As a result, we captured both substrate and product analogues using the wild-type enzyme and the active-site mutant, which degraded substrates to different degrees (see below). Two TDA substrates, namely DUE-2,4-TDA (Fig. 5 ▸*c*) and MUE-2,4-TDA (Fig. 5 ▸*e*), two degradation products, 2,4-TDA (an amine; Fig. 5 ▸*f*) and 2,4-TDA (a carbamic acid; Fig. 5 ▸*d*), and 2,4-TDI (a substrate for TDA synthesis; Fig. 5 ▸*b*) are obtained at high resolution, with visible density for all parts of the ligands in maximum-likelihood weighted 2*mF*_o_ − *DF*_c_ standard maps at a 1.0σ contour level and no significant *mF*_o_ − *DF*_c_ difference map features at a ±3σ contour level. Clear omit maps for ligands also confirm the binding (Supplementary Table S3). Soaking of MDA-related compounds results in no clear density for ligands, even though partial/weak densities are observed at low contour levels in *mF*_o_ − *DF*_c_ maps, but no further modelling of ligands was performed.

All TDA fragments bind aP2, which was previously mapped by fragment screening as a potential binding site (Fig. 3 ▸*a*). Indeed, the binding of aromatic ring groups of fragments overlap with the toluenediamine ring of TDA fragments (Supplementary Fig. S7*f*). Binding of long fragments displaces water molecules otherwise bound in the ligand-free structure (Supplementary Fig. S7*b*). The toluenediamine ring is aligned almost perfectly in the same orientation in different TDA fragments in aP2 (Fig. 5 ▸*a*). The long ethylene glycol tails of DUE-2,4-TDA (Fig. 5 ▸*c*) and MUE-2,4-TDA (Fig. 5 ▸*e*) also bind and extend towards the same long tunnel in aP2, making several contacts with backbone groups of surrounding residues in the pocket. Geometrically, the tail segment fits very nicely into the pocket, indicating lock-and-key-based protein–ligand interaction, even though no significant hydrogen-bonding contacts or salt bridges are observed. Soaking of DUE-2,4-TDA into wild-type amidase U11 crystals results in *in crystallo* degradation and the capture of 2,4-TDA carbamic acid. In the proposed reaction mechanism, urethane bond cleavage occurs via the release of an alcohol and a carbamic acid group, which subsequently hydrolyses spontaneously to an amine group (Paiva *et al.*, 2025 ▸). However, the trapped TDA fragment is not directly exposed to bulk solvent and is therefore stabilized in a relatively hydrophobic cavity of aP2 that might prevent the spontaneous hydrolysis of the carbamic acid group of 2,4-TDA (Fig. 5 ▸*d*). A similar carbamic acid moiety is also observed in the MUE-2,4-TDA-bound structure in the active site of the mutant U11-S173A. This is most likely caused by residual activity remaining in the catalytic pocket from other serine residues in the hydrogen-bonding network, as has also been observed in serine proteases (Hedstrom, 2002 ▸). In summary, aP2, which is probed and mapped by fragment screening, indeed favours binding to TDA-based PU. Since both substrates and products bind to this pocket in a catalytically unfavourable orientation, aP2 is most likely related to binding and stabilizing bulky TDA plastic to guide it towards the catalytic pocket. This observation confirms the potential role of aP2 in binding bulky, hydrophobic fragments, as shown by fragment-based pocket probing in the previous section. Moreover, reduced amidase activity towards TDA-based PU, compared with MDA-based PU (Supplementary Fig. S8*b*), suggests potential product inhibition due to binding of TDA fragments, as the relative concentration of the product (*e.g.* MUE-TDA and 2,4-TDA) increases throughout catalysis and might remain bound to aP2, which restricts the binding of the new substrate, as we saw in the MUE-TDA-bound (Fig. 5 ▸*e*) and 2,4-TDA-bound (Fig. 5*f*) structures.

#### 3.3.2. Esterase PU fragment-bound structures show distinct modes of MDA-based and TDA-based PU binding

Fragment soaking of PU fragments to esterase AX^PUR5^ results in four high-resolution structures bound to 4,4-MDA, 2,4-TDA (carbamic acid), DUE-2,6-TDA and DUP-2,6-TDA (Fig. 6 ▸*c*). All TDA-related fragments bind approximately at the same site, with slight differences in the flexible ethylene glycol arms. Toluene groups of fragments are located in eP2, with arms extending towards eP3. The urethane bond in TDA fragments is near the nucleophilic serine, suggesting a plausible catalytic orientation (Figs. 6 ▸*b*, 6 ▸*d* and 6 ▸*e*). On the other hand, 4,4-MDA, the fully degraded product of DUE-MDA, is slightly shifted from the TDA fragment location towards the eP1 location (Fig. 6 ▸*a*). The first benzylic ring of 4,4-MDA makes a direct hydrogen bond with the nucleophilic serine via its amine group, whereas the second benzylic ring of 4,4-MDA occupies the same location as fragment VT00227 (Supplementary Fig. S9*d*) which mapped eP1. Considering the hydrogen-bonding distance, we captured the leaving group from the carbamate bond-degraded MDA-based polyurethane. Gln111 makes a hydrogen bond to the carbonyl oxygens of the urethane bond of all TDA-related fragments. A conserved water molecule (*i.e.* water 50) in the TDA binding pocket is found to interact with the amine group of the urethane bond of TDA fragments. This water molecule might be important for catalysis due to its proximity to the nucleophilic serine (Figs. 6 ▸*b*, 6 ▸*d* and 6 ▸*e*).

Surprisingly, the PU fragments induce a conformational change in the peptide trapped between Lid1 and Lid2 (Supplementary Fig. S2). Binding of 2,4-TDA releases the peptide fragment due to a steric clash (Fig. 6 ▸*b*). In contrast, binding of DUE-2,6-TDA and DUP-2,6-TDA changes the peptide location and conformation completely, from the initial position (pose 1) to a more solvent-exposed position (pose 2) (Supplementary Figs. S10*b* and S10*d*). Both 2,6-TDA-related fragment structures are resolved in the presence of the peptide, forming what we term double-ligand structures, along with the 4,4-MDA-bound structure. The major difference between 2,4-TDA- and 2,6-TDA-related fragments is the location of the methyl group (*para* in 2,4-TDA and *ortho* in 2,6-TDA; Fig. 1 ▸). The exact reason for peptide release in 2,4-TDA and displacement in 2,6-TDA remains unclear. It might relate to the initial binding mode of the fragments. On the other hand, 4,4-MDA binding does not alter the spatial orientation or position of the peptide, since the 4,4-MDA binding site is located away from the peptide binding site, implying no steric clash (Supplementary Figs. S10*a* and S10*c*). Moreover, when we soaked 4,4-MDA into PMSF-purified esterase crystals, we obtained a triple-ligand complex in which 4,4-MDA, PMS and peptide are bound to the esterase, although with reduced occupancy, as observed by a weaker but still positive unbiased omit map (*mF*_o_ − *DF*_c_) at the 2.5σ contour level (Supplementary Fig. S10*a*). We also noticed that the benzylic ring of PMS and the benzylic ring of phenylalanine from the peptide occupy the same location, consistent with partial occupancy of PMS and the peptide in different unit cells. There is no alternative conformation for phenyl­alanine from the peptide to occupy. Therefore, we modelled the peptide as NLYAQ by substituting phenylalanine with alanine. Even though the trapped peptide is not part of the structure, it acts as an extension of the loop in the Lid1 domain, which contributes to the binding of plastic fragments by covering the active site and increasing the overall hydrophobicity.

In summary, we obtained four high-resolution PU fragment-bound structures of esterase AX^PUR5^: first with 2,4-TDA bound with displaced peptide, then three structures in the presence of 4,4-MDA and TDI fragments along with the peptide in different conformations (double ligand), and finally in the presence of 4,4-MDA, PMS and peptide (triple ligand). Comparison with fragments that are used to map binding sites reveals that aromatic moieties of fragments located at similar locations for eP1 (*i.e.* VT00227) and eP2 (*i.e.* VT00401) and makes similar type of interactions (Supplementary Figs. S9*e* and S9*f*). On the other hand, the flexible tails of 2,6-TDA fragments partially overlap with aromatic groups of fragments (*i.e.* VT00446) at eP3 (Supplementary Fig. S9*g*). All of these structures imply extensive binding of PU fragments to the large active site of the esterase, in which fragments bind almost the entire available active-site cavity. Since the active site of the esterase is larger and more solvent-exposed than that of the amidase, the inherent flexibility of fragments is observed on the urethane arms of 2,4-TDA- and 2,6-TDA-related fragments, as evinced by electron-density maps (Figs. 6 ▸*d* and 6 ▸*e*). We propose that the large and solvent-exposed active-site cavity of esterase should favour the binding of bulky PU fragments, as seen for double- and triple-ligand-bound structures. The 2,4-TDA-bound structure implies *in crystallo* degradation of DUE-TDA; however, this also leads to product inhibition, since 2,4-TDA should compete with the binding of substrate to the active site, as also observed in the residual level of activity for DUE-TDA-based PU (Bendtsen *et al.*, 2026 ▸). In addition, improper positioning of the reactant groups of 2,6-TDA-based PU fragments due to the position of the methyl group (Figs. 1 ▸*e* and 1 ▸*f*) with respect to scissile urethane bonds indicates a steric clash with active-site residues and hence also results in an unproductive and inhibitory binding pose. Finally, the orientation of 4,4-MDA suggests more favourable binding of MDA-based PU and catalysis by the esterase catalytic scaffold, as confirmed by the demonstrated activity of the esterase against MDA-based PU (Bendtsen *et al.*, 2026 ▸).

### 3.4. Transition-state analogues provide catalytic snapshots of PU by extensively probing pockets

Finally, we turned to other types of inhibitors to obtain mechanistic details of the binding and stabilization of catalytic intermediates and transition-state analogues of PU. Inspired by PMSF, novel suicide inhibitors that mimic MUE-MDA and MUE-TDA were designed (Teixeira *et al.*, 2026 ▸), which we synthesized and soaked into the two enzymes. Acylation of the substrate urethane bond and the subsequent tetrahedral intermediate are shown to be common steps in AS amidases (Cerqueira *et al.*, 2017 ▸). Upon reaction with the nucleophilic serine, suicide inhibitors form covalent phosphoester bonds that mimic the hypothetical intermediate transition state during the first step of nucleophilic attack (acylation), in which serine hydrolyses the scissile carbonyl carbon (Teixeira *et al.*, 2025 ▸; Paiva *et al.*, 2025 ▸).

#### 3.4.1. The MUE-MDA TS analogue binds to two different conformations in the amidase active site

**Figure 7:**
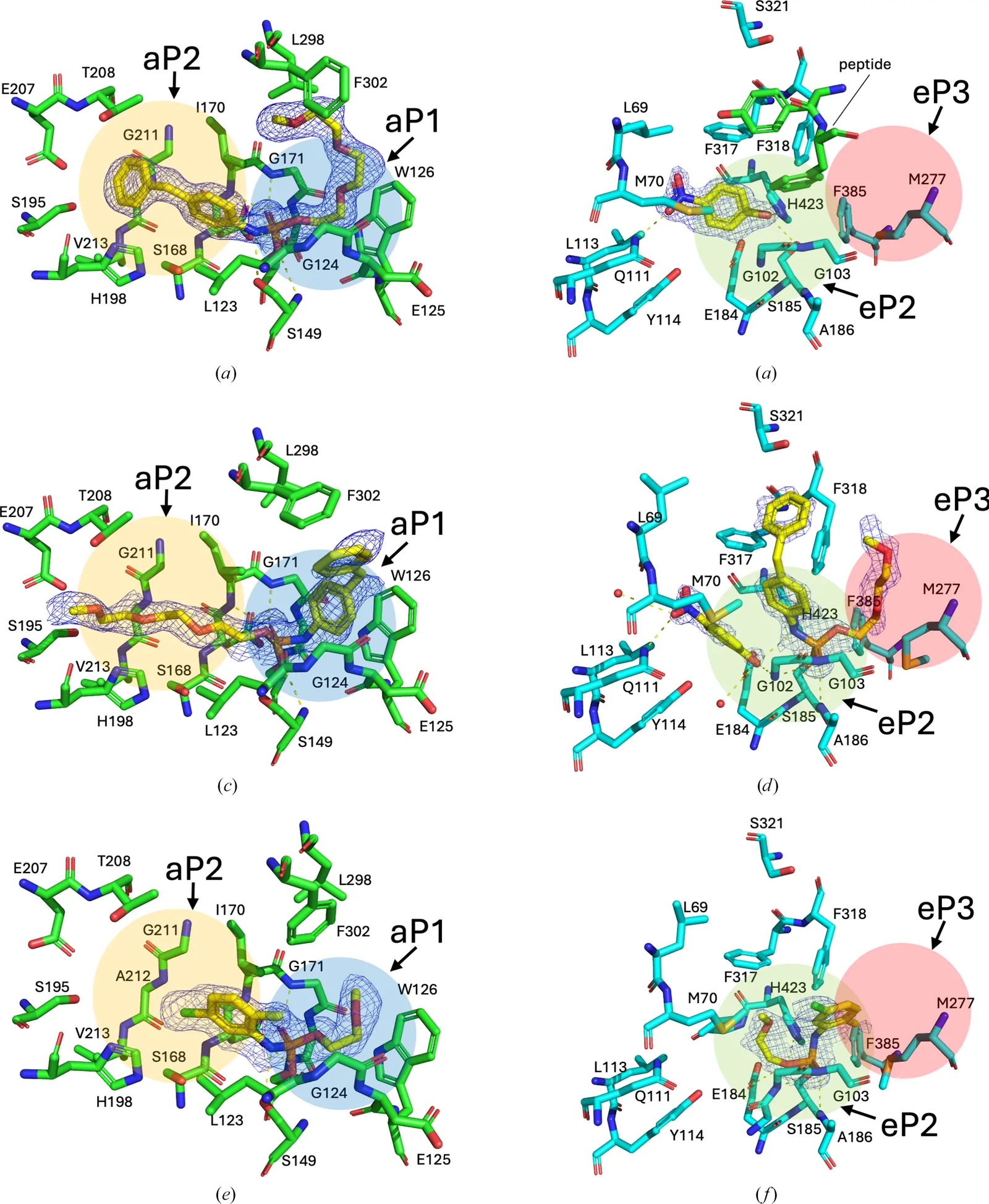
MUE-MDA and MUE-2,4-TDA transition-state analogues bound to amidase U11 and esterase AX^PUR5^. (*a*) U11 bound to the MUE-MDA TS analog in pose 1 (PDB entry 30ge). (*c*) U11 bound to the MUE-MDA TS analog in pose 2 (PDB entry 30gv). (*e*) U11 bound to the MUE-2,4-TDA TS analog (PDB entry 30tu). (*b*) Esterase AX^PUR5^ bound to the leaving group *p*-nitrophenol (PDB entry 29nw). (*d*) Esterase AX^PUR5^ bound to the MUE-MDA TS analog (PDB entry 30tv). (*f*) Esterase AX^PUR5^ bound to the MUE-2,4-TDA TS analog (PDB entry 30va). 2*mF*_o_ − *DF*_c_ standard maps at a 1.0σ contour level are shown for fragments as a blue mesh.

The MUE-MDA TS analogue was soaked for different times ranging from 10 min to overnight. Analysis of the crystal structures clearly indicates that the formation of a covalent bond between the catalytic serine and the phosphate of the inhibitor takes place within 30 min. However, the appearance of clear density for the MDA ring structure and the flexible ethylene glycol tail requires a minimum of 1 h of soaking, and complete density for ethylene glycol tails appeared after several hours (a minimum of 4 h; Supplementary Fig. S11). The large flexibility of the long ethylene glycol tail explains this behaviour, which is also reflected by clear residual densities in the difference map, even after several rounds of refinement without adding the ligand to the model. Strong density is observed near the catalytic serine at shorter times, which implies the presence of the phosphoryl group. This makes it evident that relaxation of the fragment, especially the ethylene glycol tail, and rotation of the methylene bridge in the MDA ring, require more time to reach a stable conformation. Remarkably, our crystal structures also indicate that the inhibitor binds in two different conformations. In the first mode of binding (Fig. 7 ▸*a*) the ethylene glycol tail is oriented towards aP1 and bent to fit into the space created by the flipping of Phe302. On the other side, the MDA ring fits nicely into the junction between aP1 and aP2, with a very good density in the 2*mF*_o_ − *DF*_c_ map, even at higher contour levels (1.5σ–1.7σ); however, the ethylene glycol tail has complete density at the 1σ contour level, as expected due to flexibility. In the second binding pose (Fig. 7 ▸*c*) the MDA ring is oriented into aP1 and makes a T-shaped aromatic π–π stacking interaction with Trp126 through its first benzylic ring (Supplementary Fig. S7*e*). The second benzylic ring of the inhibitor extends into the hydrophobic channel, as described in PMS (Fig. 2 ▸*e*), but shows weak/partial density, possibly because of flexibility or a lack of clear interaction with nearby residues. The ethylene glycol tail in this second pose extends into aP2 and has very good density for all parts, implying tighter interaction compared with the first binding mode. Altogether, the observation of two different binding modes for the MUE-MDA TS analogue indicates the versatility of the active site for binding to MDA-based PU fragments in different orientations. Moreover, clear MDA-based PU degradation activity by the amidase (Rotilio *et al.*, 2026 ▸) supports the mechanistic basis of promiscuous binding to MDA-based PU.

#### 3.4.2. The MUE-2,4-TDA TS analogue binds to the same pocket as TDA fragments in amidase

In the MUE-TDA TS analogue-bound crystal structure, we observed a similar tetrahedral covalent adduct in which a phosphoester bond is formed between O^γ^ of Ser173 and the phosphate of the TS (Fig. 7 ▸*e*). Stabilization of the double-bonded tetrahedral oxygen by the oxyanion hole is also observed, similarly to PMS and the MUE-MDA TS analogue. The aromatic group of the TS analogue fits into aP2, and the ethylene glycol tail extends towards aP1. In comparison with the TDA fragment-bound structures, we see that the aromatic group of the TS analogue is shifted toward the catalytic pocket. This state reproduces the catalytic snapshot during degradation, demonstrating that the TDA moiety would be trapped in aP2 upon cleavage of the urethane bond, in clear agreement with our observations on other TDA fragment-bound structures. Further release of TDA from aP2 and the subsequent binding of a new TDA substrate would require loop motions. However, the additional ethylene glycol tail in the TDA group, which extends deep into the proximal pocket, hinders the release of product.

#### 3.4.3. The MUE-MDA TS analogue and leaving group captured in esterase

In the proposed catalytic mechanism for carboxylesterases, a similar tetrahedral intermediate is formed upon nucleophilic attack of the catalytic serine on the carbonyl carbon of amide, ester or carbamate bonds (Aranda *et al.*, 2014 ▸). In this scenario, it could be expected that our TS analogues would also bind to esterase AX^PUR5^. To validate this theory, we first purified the esterase in the presence of the MUE-MDA TS analogue and, furthermore, we co-crystallized and soaked for a long time in the first trial. Surprisingly, no structures contained a covalent bond between the TS analogue and esterase AX^PUR5^; instead, we found the leaving group *para*-nitrophenol located close to eP2 (Fig. 7 ▸*c*), establishing a hydrogen bond to the nucleophilic serine via its hydroxyl group, which resembles the fragments bound to eP2 (Fig. 4 ▸*a*). On the other hand, the nitro group formed another hydrogen bond to Gln111. Since *para*-nitrophenol bound tightly, we reasoned that an excess amount of *para*-nitrophenol remaining after synthesis and purification of the MUE-MDA TS analogue could remain bound to the active site. Thus, we synthesized a new batch of the MUE-MDA TS analogue in which free *para*-nitrophenol is removed more thoroughly and tried soaking again. In this case, a covalent adduct between nucleophile Ser185 and the phosphoryl group formed. The ethylene glycol tail extends into eP3, which is surrounded by several aromatic and aliphatic side chains, while the MDA core is on the other side of the pocket, where it is surrounded by several hydrophobic residues (Fig. 7 ▸*d*). Moreover, we also located the leaving group *para*-nitrophenol, which is released and captured just after the covalent bond is formed. Interestingly, one of the oxygens from the nitro group makes the same hydrogen-bonding interaction with Gln111 as we also observed in TDA fragment structures (Figs. 6 ▸*b*, 6 ▸*d* and 6 ▸*e*). Moreover, the presence of the leaving group results in the upward conformation for the MDA core, which is almost orthogonal to 4,4-MDA. The second benzylic ring of MDA faces towards Ser321, which might have made a potential hydrogen bond in the MUE-MDA-bound structure (Fig. 7 ▸*d*). It is highly probable that once the leaving group has been released, the MDA group fits into a similar site as the 4,4-MDA product. Additionally, a water molecule that is near to the hydroxyl group of *para*-nitrophenol (Fig. 7 ▸*d*) establishes a hydrogen bond similar to the conserved water molecule that we see in TDA fragments. Taken altogether, this water molecule is most likely to be the catalytic water molecule that attacks the urethane bond of TDA fragments and the tetrahedral TS.

#### 3.4.4. The MUE-2,4-TDA TS analogue inhibitor binds in a different pose to TDA fragments in the esterase

The structure of AX^PUR5^ bound to an MUE-2,4-TDA TS analogue was solved at 1.4 Å resolution (Fig. 7 ▸*f*). We expected to see a TDA group that fits into the same site (*i.e.* eP2 and eP3) as other TDA fragments (Fig. 6 ▸*c*). However, refinement of the TDA-bound suicide inhibitor was not straightforward, as we still observed positive difference peaks and a vague standard map associated with the fragments (Fig. 7 ▸*f*) after several rounds of occupancy refinement. Nevertheless, the clear omit map for the fragment confirmed the presence of an MUE-2,4-TDA TS analogue (Supplementary Table S2). This observation most likely reflects multiple conformations of the fragment. The lack of a single-state conformation for the ligand indicates weak stabilization of the transition-state intermediate, which is essential for lowering the energy barrier for catalysis. We can see that the TDA group is located close to Met277 and Phe318, where the ethylene glycol tails of TDA bind. This binding pose for the toluene group differs from the 2,4-TDA fragment binding pose (Fig. 6 ▸*b*), which might also explain why the latter binding pose for 2,4-TDA-based PU is not suitable for catalysis. However, residual level TDA degradation activity by the esterase (Bendtsen *et al.*, 2026 ▸) suggests potential product inhibition, even though the first nucleophilic attack on the urethane bond yields a tetrahedral TS, as observed in this structure; subsequent hydrolysis of this intermediate might be the rate-limiting step, followed by binding of the 2,4-TDA degradation product to the catalytic pocket as observed in the 2,4-TDA-bound (carbamate; PDB entry 29nv) structure.

## 4. Discussion

In this study, we have demonstrated the efficiency of the fragment-based active-site exploration (FASE) approach to characterize interactions of different urethanases with various PU fragments. Our results highlighted several key features that could be essential to elucidate enzyme–plastic interaction at the molecular level. First, we mapped two binding pockets in amidase U11 (*i.e.* aP1 and aP2) and three pockets in esterase AX^PUR5^ (*i.e.* eP1, eP2 and eP3) in which our fragments are found to be clustered. Moreover, probing the active site of enzymes with fragments permitted the exploration of the binding landscape of novel enzymes for which real substrates are not exactly known. The simplicity of fragments that are used to map the active site effectively covers chemical space for potential subpockets that can be important for substrate recognition. Additionally, we confirm the role of these probed sites as potential plastic fragment-binding ‘hot spots’ using a wide range of short, soluble plastic fragments that are analogues of the substrate, transition state, intermediate and product. The mimicking of aromatic toluene and dianiline rings of PU by fragments clearly shows the applicability of the fragment-based approach as we probe similar types of interactions at mapped pockets (Supplementary Figs. S7 and S9). By saturating binding pockets with fragment-screening campaigns, we uncovered the important sites in the active sites for PU binding and recognition. Additionally, by determining several structures of amidase and esterase in the presence of various PU fragments, we demonstrate the distinct binding modes of MDA- and TDA-based PU to completely different catalytic scaffolds and uncover the mechanistic basis of the differential activities of enzymes towards MDA- and TDA-based plastics. As a result, we demonstrated that by probing the active site with small-molecule compounds, we could map the actual plastic-binding sites that are in catalytically favourable orientations and positions within the large active-site cavity of our target enzymes.

### 4.1. Extensive PU binding by the entire active site of esterase AX^PUR5^

In the esterase, different binding modes of MDA- and TDA-based PU fragments were observed due to the large hydrophobic active-site cleft, which exhibits a high degree of substrate promiscuity, as shown in our fragment-screening, plastic ligands and trapped peptide fragment-bound structures. Depending on the active-site shape, the MDA and TDA cores are positioned differently, either in catalytically favourable or inhibitory poses. By targeting the surrounding residues around the TS analogues, it is possible to stabilize and hence reduce the activation energy that is required for catalysis or facilitate the release of the leaving group by weakening the interaction between the product and eP1 residues. Notably, the capture of *para*-nitrophenol and 4,4-MDA in the same binding site highlights the location for the route of product release, which can be further engineered to reduce steric hindrance and weaken favourable interactions with the leaving group. On the other hand, all TDA fragments bind to eP2 with almost the same orientation of the aromatic moiety and urethane bonds. In contrast, flexible ethylene glycol tails sample multiple conformations, as is evinced by weakened electron-density maps towards eP3 (Figs. 6 ▸*b*, 6 ▸*d* and 6 ▸*e*). Despite the favourable position of urethane bonds of TDA fragments for catalysis, the presence of a methyl group in the toluene ring prevents the proximity between the nucleophilic serine and the urethane bond for actual catalysis, thus correlating with the reported residual activity of this esterase for TDA-based plastic (Bendtsen *et al.*, 2026 ▸). There are a few carboxylic esterases that are reported to degrade PU (Bendtsen *et al.*, 2026 ▸). Interestingly, we only find one homologous esterase for which the structure has been deposited in the PDB (PDB entry 8xrz) along with an MDA degradation product-like MDA analogue suicide inhibitor that we used in this study. The aniline moiety of the MDA degradation product is located in a site analogous to eP3 and the peptide-binding site that we show in our structures. In contrast, the MDA fragments that we observed in our structures (Fig. 6 ▸*a* and Fig. 7 ▸*d*) are located in a different site than in PDB entry 8xrz, which could be associated with local residue differences in eP3 and the peptide-binding site. Nevertheless, this observation further enhances our conclusion, as we expected to see bulky PU fragments in eP3 and the peptide-binding site. Altogether, for a catalytically favourable conformation, the MDA group favours orientation towards eP1, whereas TDA groups are buried toward eP2 that is mapped by fragment screening. Moreover, the large hydrophobic active site of AX^PUR5^ offers the opportunity for substrate promiscuity through multiple binding conformations, although some binding poses may be unproductive or inhibitory.

### 4.2. Binding and guidance of the bulky PU fragment by aP2 for catalysis in amidase U11

In the case of amidase U11, aP2, which was mapped by our fragment screening, clearly stabilizes the aromatic moiety of TS analogues and binds the substrate and product analogues of TDA. Moreover, in the homologous urethanase UMG-SP2, the same pocket binds to 4-hydroxybutyl-[4-(4-aminobenzyl)phenyl] carbamate (BBC), which is a short-chain carbamate (Li *et al.*, 2025 ▸). Even though BBC is not a true mimetic of either the toluene or aniline ring of PU, it further supports our observations that aP2 is important for binding bulky fragments. The relevance of aP1 and the nearby specificity pocket, which bind to small aromatic carbamates in a favourable conformation for catalysis and product release, is highlighted by the MDA TS analogue-bound structure in this study (Fig. 7 ▸*c*), the degradation product 4,4-MDA-bound structure in a homologous urethanase (Rotilio *et al.*, 2026 ▸) and QM/MM calculations on UMG-SP2 (Świderek *et al.*, 2025 ▸). In other AS amidases, such as FAAH (Min *et al.*, 2019 ▸) and malonamidase (Shin *et al.*, 2003 ▸), analogous specificity pockets and surrounding binding sites are observed, with differences in polarity and shape that enable binding to different types of substrates. This observation again highlights the specificity required for catalytically productive PU fragment binding. There are two distinct binding poses for the catalysis of the urethane bond in MDA- and TDA-based PUs. The orientation of the aromatic moiety of both TDA and MDA can either fall into aP2 (Fig. 5 ▸*a*) or extend towards aP1 and the substrate-release channel (Fig. 2 ▸*e*) in the case of MDA. In the first scenario, loop dynamics are required to release the product, suggesting that further optimization of loops could increase the catalytic turnover by enhancing flexibility. Indeed, engineering studies of homologous enzymes show that mutating residues on the same loop that covers aP2 significantly improves the catalytic activity (Bayer *et al.*, 2024 ▸; Li *et al.*, 2025 ▸; Rotilio *et al.*, 2025 ▸). In the second scenario, the specificity pocket at aP1 (Fig. 2 ▸*e*) interacts with and stabilizes the first benzylic ring of the MDA moiety, as demonstrated in this study by PMS and described in Rotilio *et al.* (2025 ▸) (Fig. 2 ▸*e*) and the MDA-TS analogue inhibitor (Fig. 7 ▸*c*), and in previous docking and MD simulations (Paiva *et al.*, 2025 ▸; Zheng *et al.*, 2026 ▸). However, for TDA-based PU, aP1 cannot bind to the toluene ring due to steric clashes with the amine and methyl groups. Furthermore, fragment screening also indicates that all fragments bind to aP2, whereas only three fragments with a single aromatic ring probe aP1 (Fig. 3 ▸*a* and Table 1 ▸). However, aP2 needs to be carefully investigated for engineering since the same site also binds various TDA fragments, including substrate and product, in which extensive stabilization of aromatic groups of PU fragments by engineering might lead to product inhibition and reduce catalytic turnover. Release of the product can be facilitated by engineering the loop that covers aP2 by improving flexibility and reducing hydrophobic interactions, which again emphasizes the important role of loops in ligand binding and turnover. In summary, aP2 is important for binding and stabilizing long, bulky PU fragments for catalysis, whereas the aromatic groups of PU fragments need to bind to aP1 and extend into the hydrophobic channel to favour favourable product-release orientation and catalysis.

### 4.3. Structural features of different catalytic scaffolds for PU binding and degradation

**Figure 8:**
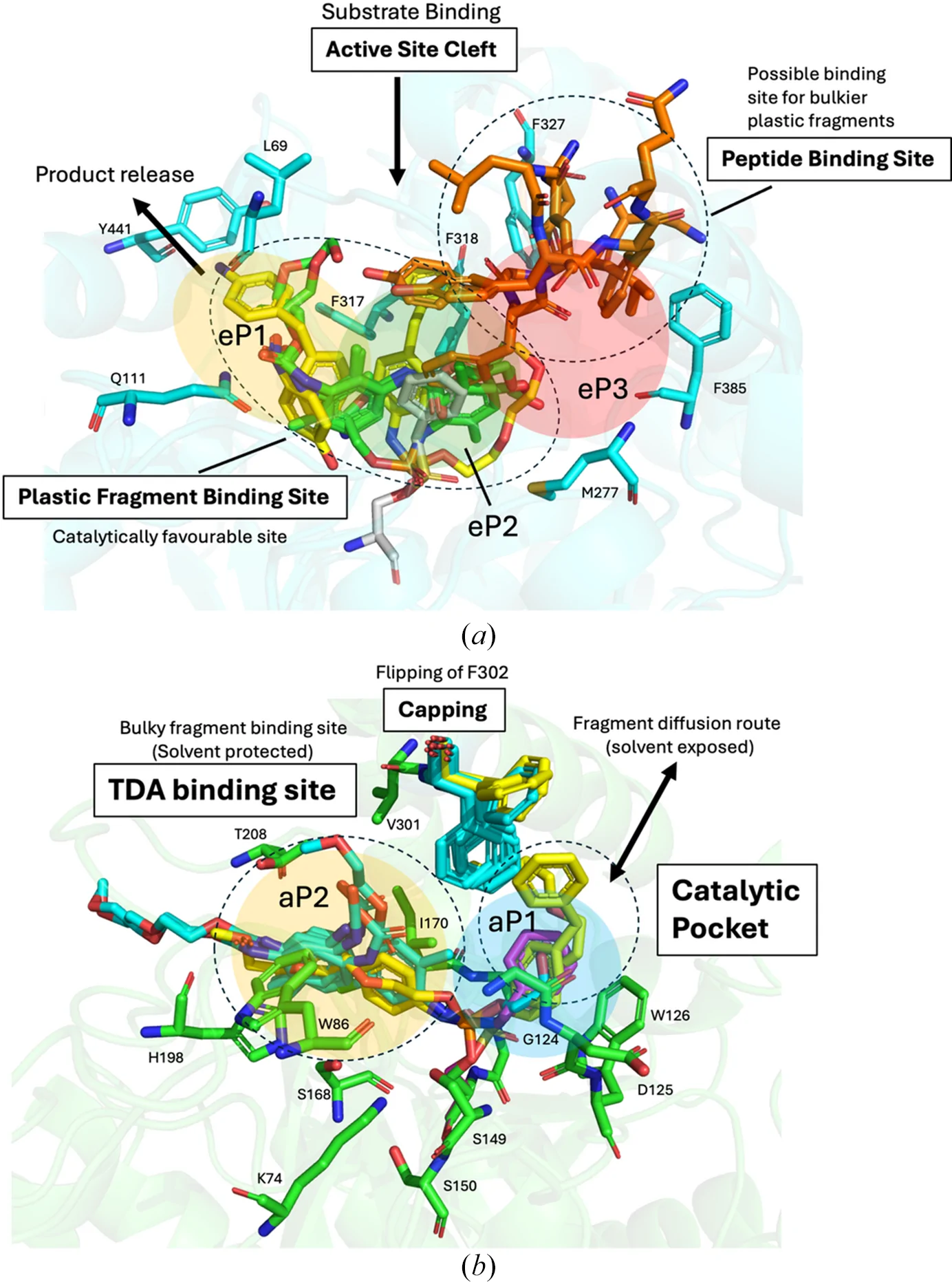
Summary of structural features of amidase and esterase identified by the FASE approach. (*a*) Esterase structural features for plastic fragment binding. (*b*) Amidase structural features. Mapped pockets are shown as shaded colours, and important binding sites are shown as dashed circles.

The fragment-based approach for urethanases identifies several important features for the binding and recognition of PU fragments (Fig. 8 ▸). For esterase AX^PUR5^, we unravelled key structural features as (i) a product-release route in eP1, (ii) promiscuous PU binding sites that span eP2 and eP3, (iii) substrate access by a large hydrophobic cleft and (iv) potential binding of bulky PU fragments at the peptide pocket and eP3 (Fig. 8 ▸*a*). For amidase U11, the study revealed key structural elements, including (i) a substrate and product diffusion channel, (ii) binding of bulky PU fragments and their guidance towards the catalytic site by aP2, (iii) substrate capping by Phe302 and (iv) distinct orientations of TDA- and MDA-based PU for a catalytically favourable conformation (Fig. 8 ▸*b*).

The fragment-based approach elucidates the molecular mechanism of interaction between plastic fragments and two different catalytic scaffolds that degrade polyurethane. This mechanistic understanding of the specificity for TDA- and MDA-based PU by different catalytic scaffolds and active sites holds the potential to guide engineering and discovery campaigns for homologous enzymes efficiently, since the overall structure of active sites and binding pockets is highly conserved in several reported enzymes (Chen *et al.*, 2025 ▸; Bendtsen *et al.*, 2026 ▸; Rotilio *et al.*, 2026 ▸). Future studies can benefit from our fragment-based approach to identify ‘hot-spots’ for engineering campaigns in homologous enzymes.

## 5. Related literature

The following reference is cited in the supporting information for this article: Yeates & McPherson (2019 ▸).

## Supplementary Material

Supplementary Tables and Figures. DOI: 10.1107/S2059798326010624/ag5071sup1.pdf

PDB reference: esterase AX^PUR5^, 29jv

PDB reference: 29jy

PDB reference: 29kj

PDB reference: 29kx

PDB reference: 29nv

PDB reference: 29nw

PDB reference: 29oj

PDB reference: 29oo

PDB reference: 29qc

PDB reference: 29pw

PDB reference: 29pz

PDB reference: 29qa

PDB reference: 29qe

PDB reference: 29qs

PDB reference: 29qu

PDB reference: 29qx

PDB reference: 29rs

PDB reference: 29ru

PDB reference: 29rv

PDB reference: 29rw

PDB reference: 30tv

PDB reference: 30va

PDB reference: amidase U11, 29zk

PDB reference: 30fo

PDB reference: 30ge

PDB reference: 30gv

PDB reference: 30hf

PDB reference: 30hg

PDB reference: 30nq

PDB reference: 30pj

PDB reference: 30ob

PDB reference: 30om

PDB reference: 30op

PDB reference: 30pl

PDB reference: 30qo

PDB reference: 30qp

PDB reference: 30qw

PDB reference: 30qy

PDB reference: 30rg

PDB reference: 30sd

PDB reference: 30tu

PDB reference: 30oc

PDB reference: 34fs

## Figures and Tables

**Table 1:** List of fragments that map out binding pockets in amidase U11 Fragments that bind/do not bind to the pocket are indicated as + and −, respectively. Interacting residues within 4 Å and interaction types are listed for the corresponding pocket. Residues that are involved in hydrogen bonding are highlighted in red and residues that are involved in π–π interactions are highlighted in blue.

Fragment name	PDB code	aP1	Interacting residues at aP1	Interaction type at aP1	aP2	Interacting residues at aP2	Interaction type at aP2
VT00019	30oc	−	−	−	+	Trp86, Ala122, Leu123, Ser149, Ser168, Asp169, Ile170, His198, Tyr200, Thr208, Val301, Phe302	Hydrophobic interaction
VT00022	30om	−	−	−	+	Trp86, Leu123, Ser168, Asp169, Ile170, His198, Gly211, Ala212, Val301, Phe302	Hydrophobic interaction
VT00048	30op	−	−	−	+	Trp86, Leu123, Ser168, Asp169, Ile170, His198, Thr208, Gly211, Val301, Phe302	Hydrophobic interaction
VT00079	30pj	+	Leu123, Gly124, Asp125, Trp126, Gly148, Ser149, Asp169, Ile170, Gly171, Gly172, Ala173, Phe302	Hydrogen bonding, π–π interaction	+	Trp86, Ala122, Leu123, Ser168, Ile170, His198, Thr208, Val301, Phe302	Hydrogen bonding, hydrophobic interaction
VT00136	30pl	−	−	−	+	Trp86, Ala122, Leu123, Ser168, Asp169, Ile170, His198, Thr208, Gly211, Ala212, Val301, Phe302	Hydrophobic interaction
VT00167	30ob	−	−	−	+	Trp86, Ala122, Leu123, Ser168, Asp169, Ile170, Ser195, His198, Thr208, Gly211, Ala212, Val213, Val301	Hydrophobic interaction, π–π interaction
VT00232	30qo	−	−	−	+	Trp86, Ala122, Leu123, Ser168, Asp169, Ile170, His198, Thr208, Gly211, Ala212, Val301, Phe302	Hydrophobic interaction, π–π interaction
VT00235	30qp	+	Gly124, Asp125, Trp126, Gly148, Ser149, Ile170, Gly171, Leu298, Phe302, Leu378	Hydrophobic interaction	+	Trp86, Ala122, Leu123, Ser168, Asp169, Ile170, His198, Thr208, Gly211, Ala212, Val301, Phe302	Hydrophobic interaction
VT00248	30qy	+	Leu123, Gly124, Asp125, Trp126, Gly148, Ser149, Ile170, Gly171, Gly172, Ala173, Phe302	Hydrophobic interaction, hydrogen bonding	+	Trp86, Leu123, Ser168, Asp169, Ile170, His198, Gly211, Ala212, Phe302	Hydrophobic interaction

**Table 2:** List of fragments that mapped the pockets in esterase AX^PUR5^ Fragments that are bound to the pocket are shown as a + sign and fragments that are not bound to the pocket are shown as a − sign. Interacting residues within 4 Å and interaction types are listed for the corresponding pocket. Residues that are involved in hydrogen bonding are highlighted in red and residues that are involved in π–π interaction are highlighted in blue.

Fragment name	PDB code	eP1	Interacting residues at eP1	Interaction type at eP1	eP2	Interacting residues at eP2	Interaction type at eP2	eP3	Interacting residues at eP3	Interaction type at eP3
VT00083	29qa	−	−	−	+	Met70, Gly102, Tyr114, Glu184, Ser185, Phe317, Val424	Hydrogen bonding, hydrophobic interaction	+	Gly103, Ser185, Gln274, Met277, Trp315, Phe318, Leu384	Hydrophobic interaction
VT00085	29qc	−	−	−	+	Met80, Gly102, Gln11, Leu113, Tyr114, Glu184, Ser185, Phe317, Val424	Hydrogen bonding, hydrophobic interaction	−	−	−
VT00155	29pw	−	−	−	+	Leu69, Met70, Gly102, Leu113, Tyr114, Glu184, Ser185, Phe317, Phe318, Val424	Hydrophobic interaction	+	Gly103, Ser185, Gln274, Met277, Phe318, Leu384, Phe385	Hydrophobic interaction
VT00162	29pz	−	−	−	+	Leu69, Met70, Gly102, Gly103, Leu113, Tyr114, Glu184, Ser185, Phe317, Phe318, Val424, Val438, Tyr441, Phe442	Hydrophobic interaction	+	Gly102, Gly103, Thr105, Ala106, Ser185, Phe189, Gln274, Met277, Trp315, Phe318, Leu384, Phe385	Hydrophobic interaction, π–π interaction
VT00165	29qe	+	Leu69, Met70, Gln111, Leu113, Phe317, Ser321, Ala325, Phe415, Val438, Tyr441, Tyr442	Hydrophobic interaction	+	Met70, Gly102, Gly103, Ala106, Glu184, Ser185, Phe317, Phe318, His423, Val424	Hydrophobic interaction	−	−	−
VT00167	29qx	−	−	−	+	Leu69, Met70, Gly101, Gly102, Gly103, Gln111, Leu113, Tyr114, Glu184, Glu185, Phe317, Phe318, His423, His424, Phe442	Hydrophobic interaction	+	Gly102, Gly103, Ser185, Phe189, Gln274, Phe275, Gly276, Met277, Trp315, Phe318, Phe327, Leu384, Phe385	Hydrophobic interaction, π–π interaction
VT00217	29qu	−	−	−	+	Leu69, Met70, Gly101, Gly102, Gln111, Leu113, Tyr114, Glu184, Ser185, Phe317, Phe318, His423, Val424	Hydrophobic interaction	+	Gly103, Ser185, Phe189, Gln274, Phe275, Gly276, Met277, Trp315, Phe318, Leu384, Phe385	Hydrogen bonding, hydrophobic interaction
VT00227	29qs	+	Leu69, Met70, Leu113, Phe317, Pro437, Val438, Tyr441, Phe442	Hydrophobic interaction	−	−	−	−	−	−
VT00246	29rv	−	−	−	+	Leu69, Met70, Gly101, Gly102, Gln111, Leu113, Tyr114, Glu184, Ser185, Phe317, Phe318, His423, Val424	Hydrophobic interaction	+	Gly102, Gly103, Ser185, Ala186, Phe189, Gln274, Gly256, Met277, Trp315, Phe318, Leu384, Phe385	Hydrophobic interaction
VT00401	29rs	−	−	−	+	Leu69, Met70, Gly101, Gly102, Gly103, Gln111, Leu113, Tyr114, Glu184, Ser185, Ala186, Phe317, Phe318, His423, Val424, Phe442	Hydrogen bonding, hydrophobic interaction	−	−	−
VT00424	29rw	−	−	−	+	Leu69, Met70, Gly101, Gly102, Gly103, Gln111, Leu113, Tyr114, Glu184, Ser185, Ala186, Phe317, Phe318, His423, Val424, Phe442	Hydrogen bonding, hydrophobic interaction	−	−	−
VT00446	29ru	−	−	−	−	−	−	+	Gly102, Gly103, Ser185, Ala186, Phe189, Gln274, Gly276, Met277, Trp315, Phe318, Leu384, Phe385	Hydrogen bonding, hydrophobic interaction
