# Supplementary material for: Fragment-based active-site exploration of urethane hydrolases reveals a diversity of urethane binding modes
Source: Acta Crystallogr D Struct Biol. 2026 Oct 26;82(Pt 11):1298–317. doi: 10.1107/S2059798326010624 (PMC13717833; doi:10.1107/S2059798326010624)
Supplement: Supplementary file 1 [file d-82-01298-sup1.pdf]

STRUCTURAL BIOLOGY

Volume 82 (2026)

Supporting information for article:

Fragment-based active-site exploration of urethane hydrolases reveals a diversity of urethane binding modes

Deniz Bicer, Diana Kochubei, Rosie Graham, Samuel Peña-Díaz, Laura Rotilio, Nikolaj L. Villadsen, Andreas Sommerfeldt, Martin B. Johansen, Alexander Sandahl, Søren S. Thirup, J. Preben Morth and Daniel E. Otzen

a)

>AX^{PUR5}
MANEQTVTLSQGRYQGVPGEGVHEFRGVPYALPPLGECRFKAPQPLPDSDQVQRADRYPLPSLQRRNPLMGVEESG
EDCLYLNIWVPEDEGPFPVMLWYHGGGYTAGSVSQMLYNGAELARNGKVVVVNAAYRLGAHGFADFASVLPEADSN
LGLRDQIAALEWVNRHIEAFGGRADQVTIFGESAGGFSVASLMAAPRARPLFQRAIIQSGGADFVLSPEESRRVTEAFLA
ALPGEGDVADRLRQADGEAWLSAQGEALRTSVARGLRDSTPQFGMNFLPMVDGDLLPRVPVQAMADGACADIELLA
GVCRDEWNFFQYSGPLAGFPTVEQMRAMDDAEILRRFERAAPGNGQRLFDYYRAHVTPHPQRSNMDWFSAMESDR
LFRVPTLRLLDAQAARQPRTWGFQFTWPSTQFGIPMGACHVVDVPFVFGITDTPVGQYFTGGGEQPKQLARRVQQVW
SDFAHGRGADWTAWGESRRVHQFGPGDSETELLDQEGEALWERVIPRPETAKSENLYFQGHHHHHHHH

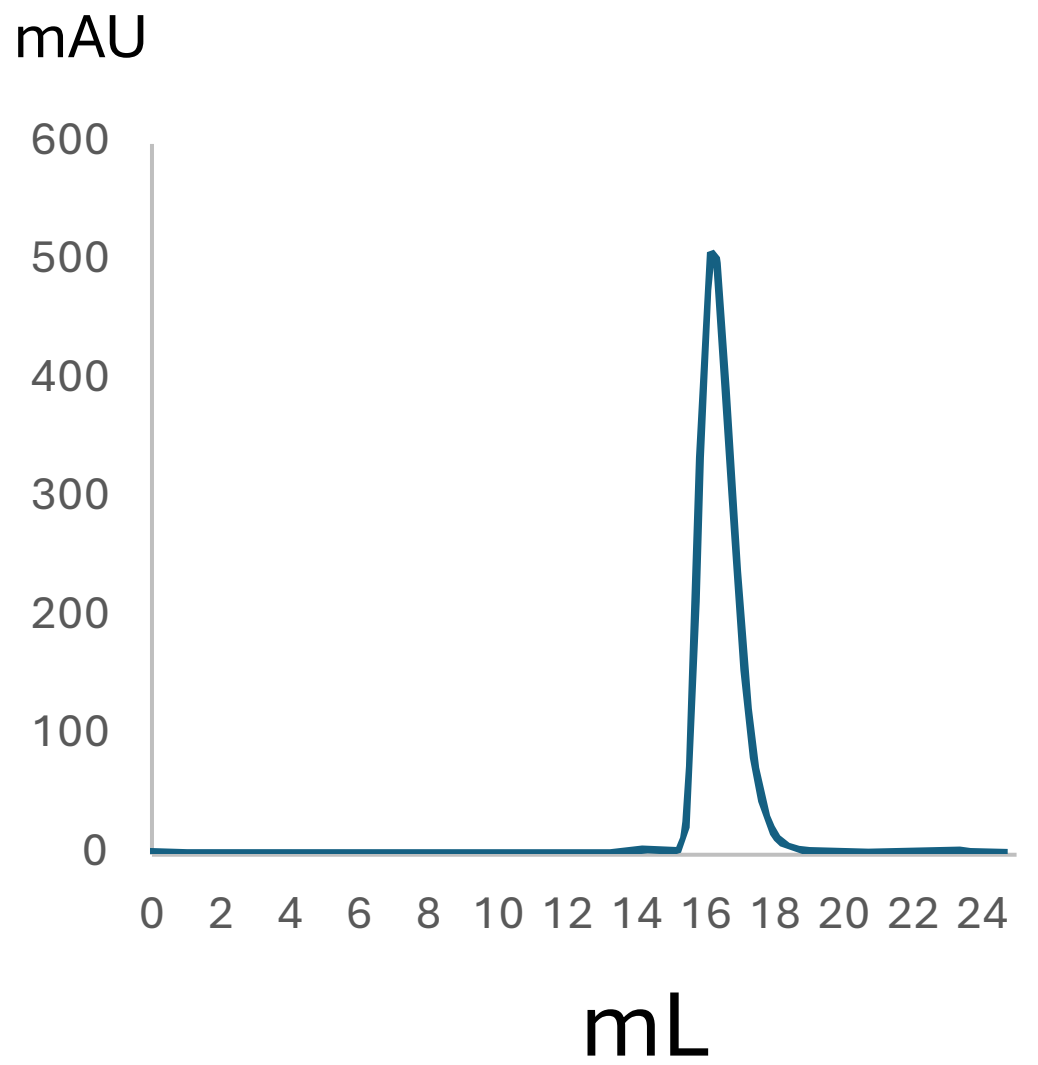

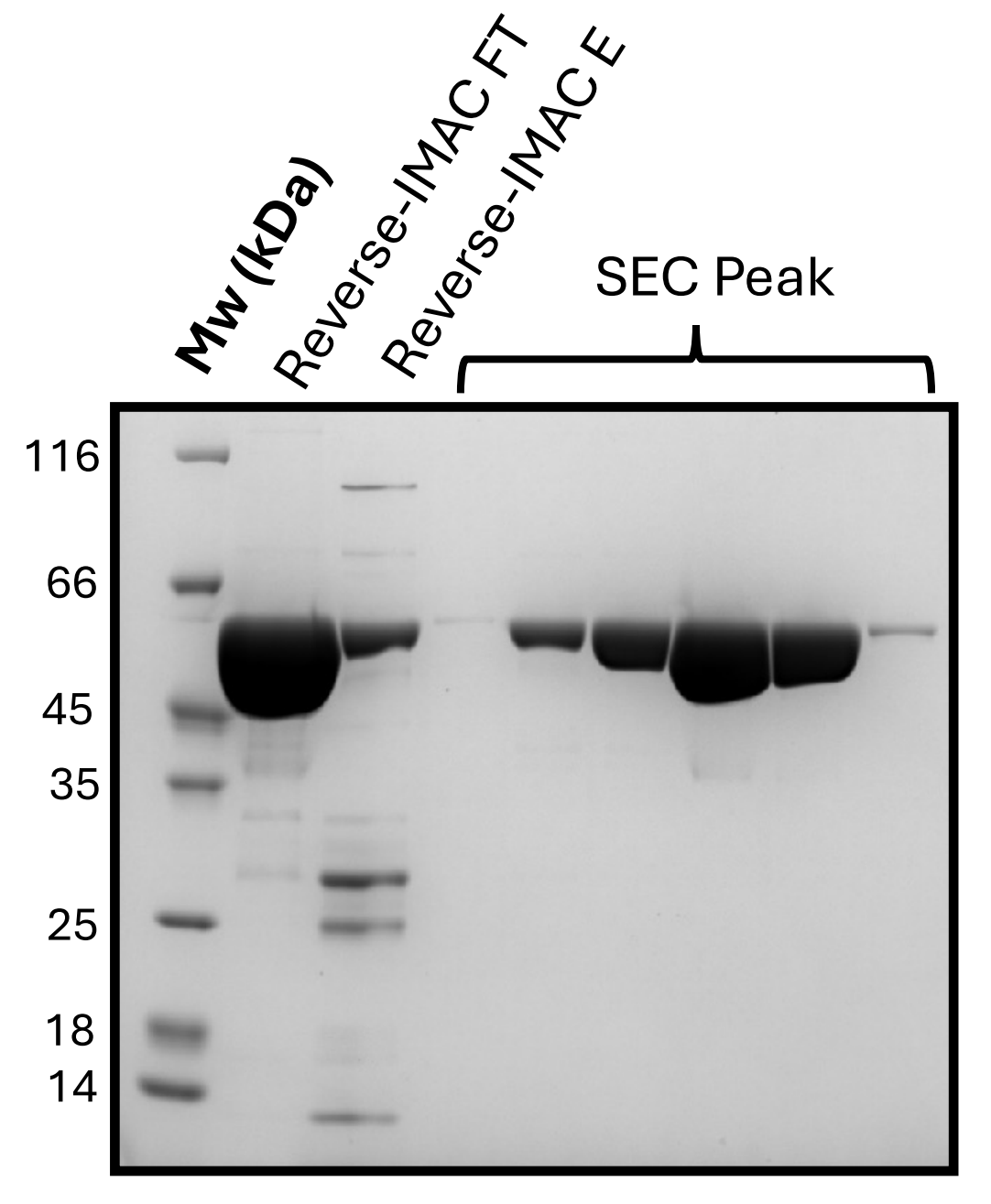

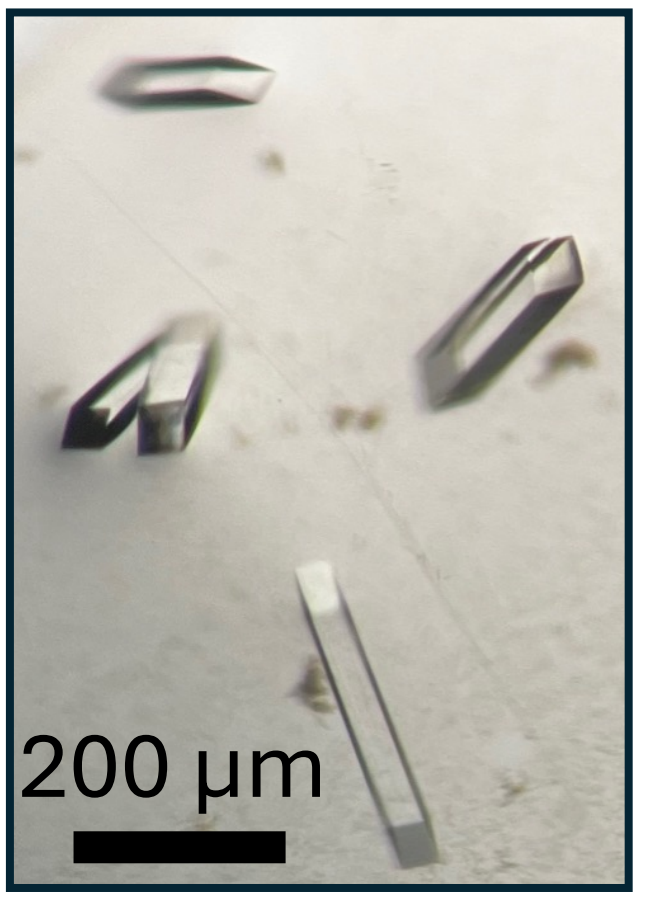

b)

>U11
MRSALATAAAIRAGETSARAETDTAIARIEAGDAAINAVVVRDFDRARAAADAIDARGPVADAMPLLGVPMTVKEAFDIEGLP
THWGLAQHAGNIATSDAEAVRRLKAAGAIILGKTNLPKALGDWQSVNSIHGRTNHPLDPTRTPGGSSGGSAAALAAGYVPI
EMGSDIGGSIRVPAHFCGVWGLKPTWNAISSLGHRYPKTDGAETVLGAVGPMARTGEDLAVLLDLLSALPLPPPRPAKRVL
ALTQHPETRTAAACVEAVEHAAEALVRAGVEVIRDHAALPDLSRQHEAYGKMLNVVFARSQPDLHHTMPTLLAWLNWLDA
QARSTRAWGALFEEVDAVIAPPAATLAFRHDSRPLAERTLDIDGVDSPYDAHLAWAGLATYPGLPACVVPIGTFDGLPGGV
QVMCAQHRDHDAIAIAQIIDTQLQGAAENLYFQGHHHHHHHH

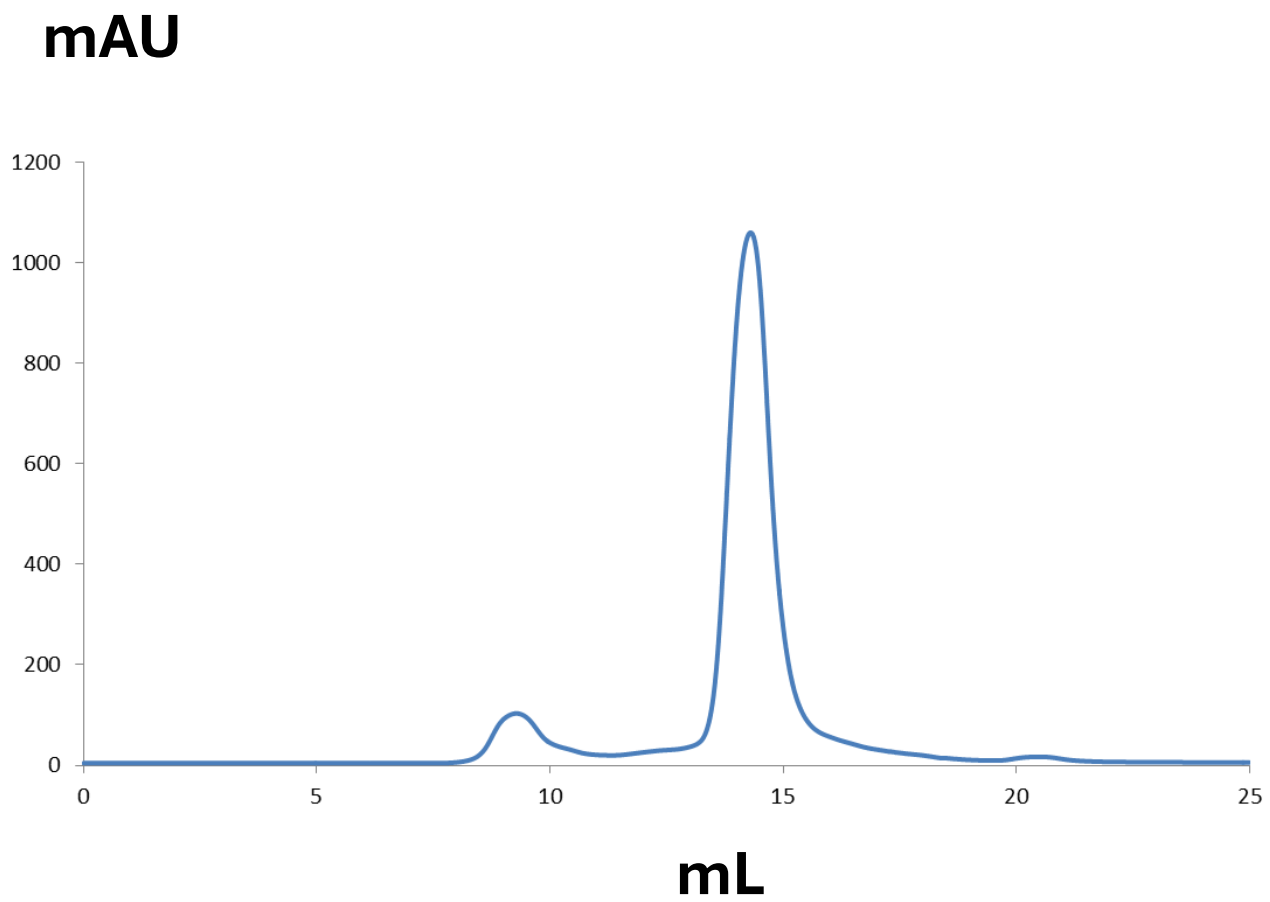

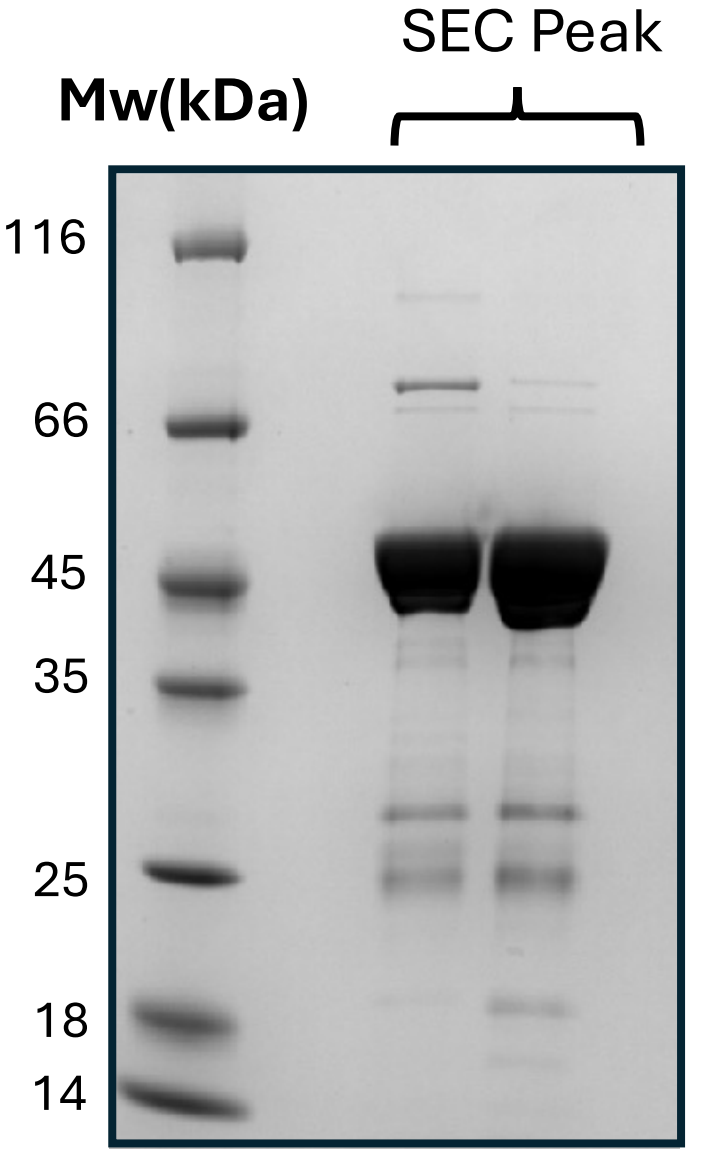

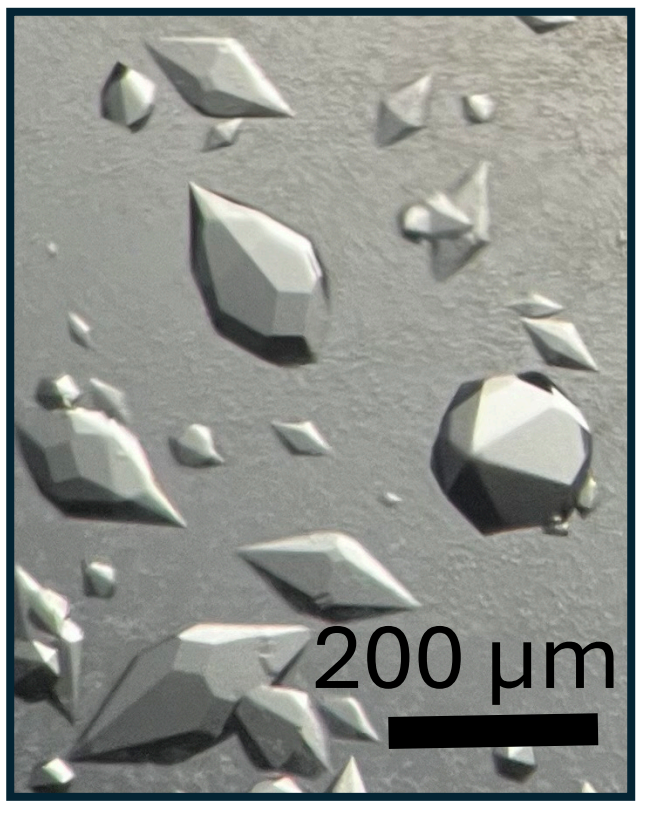

Figure S1 Sequences, purification (SEC & SDS-PAGE), and crystallization of a) AX^{PUR5} and b) u11.

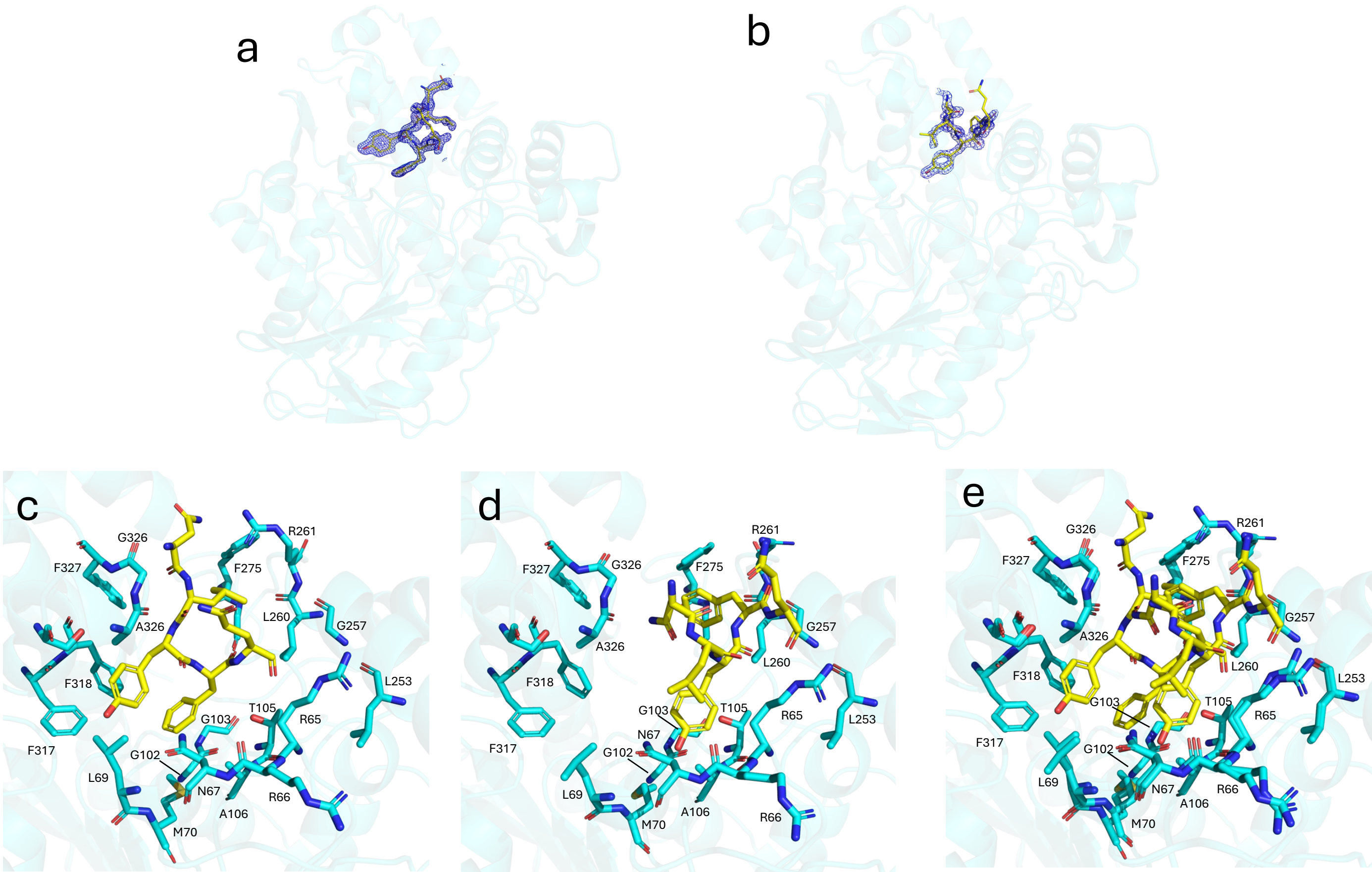

Figure S2 The change in TEV peptide conformation upon fragment binding in AX^{PUR5}. a) overall structure of AX^{PUR5}bound to the TEV peptide in pose 1 and b) overall structure of AX^{PUR5}bound to the TEV peptide in pose 1c) close view of pose-1with surrounding residues d) close view of pose-2 with surrounding residues and e) superimposed structures of pose-1 and pose-2. peptide fragment coloured as yellow for carbon atoms.

Table S1 FragMAX library hits for both enzymes.

Compound_ID	Vendor_ID	Vendor	Smiles
**VT00019**	Z2168499556	Enamine	O=S1(=O)CC(CN1)C1=CC=CC=C1
**VT00022**	EN300-35262	Enamine	N1C=NC(=C1)C1=CC=CC=C1
**VT00048**	EN300-14213	Enamine	ClC1=CC=C2NC(=O)OC2=C1
**VT00079**	EN300-49174	Enamine	COC1=C(C=CC=C1)C(N)=O
**VT00083**	EN300-221874	Enamine	CC1=CC=CN2C(N)=CN=C12
**VT00085**	EN300-30137	Enamine	NC1=CC=CC2=NC=CC=C12
**VT00136**	EN300-63856	Enamine	CNC1=NC=NC2=CC=CC=C12
**VT00155**	EN300-310875	Enamine	O=C1CCC2=CC=CC3=C2N1CC3
**VT00162**	EN300-48617	Enamine	N1C2=C(C=CC=C2)N=C1C1=CC=CN=C1
**VT00165**	EN300-6760173	Enamine	NCC1=CC2=C(CCC2)S1
**VT00167**	EN300-45212	Enamine	NC(=O)C1=C(C=CS1)C1=CC=CC=C1
**VT00217**	EN300-65251	Enamine	O=C1OCCC2=CC=CC=C12
**VT00227**	EN300-172459	Enamine	NC1=NC(=CO1)C1=CC=CC=C1
**VT00232**	EN300-138378	Enamine	C1=CN(N=C1)C1=CC=CC=N1
**VT00235**	EN300-53702	Enamine	CC1=CC=C(Cl)N=N1
**VT00246**	EN300-122828	Enamine	C1=NN2C=CC=CC2=N1
**VT00248**	EN300-1590342	Enamine	CS(=O)(=O)N1C=CC=C1
**VT00401**	EN300-17813	Enamine	CC(=O)NC1=CC=C(C=C1)C(=O)O
**VT00424**	EN300-97898	Enamine	C1=CC2=C(C=CN=C2)C=C1C(=O)O
**VT00446**	EN300-00863	Enamine	C1CCC2=C(C1)C(=CS2)C(=O)O

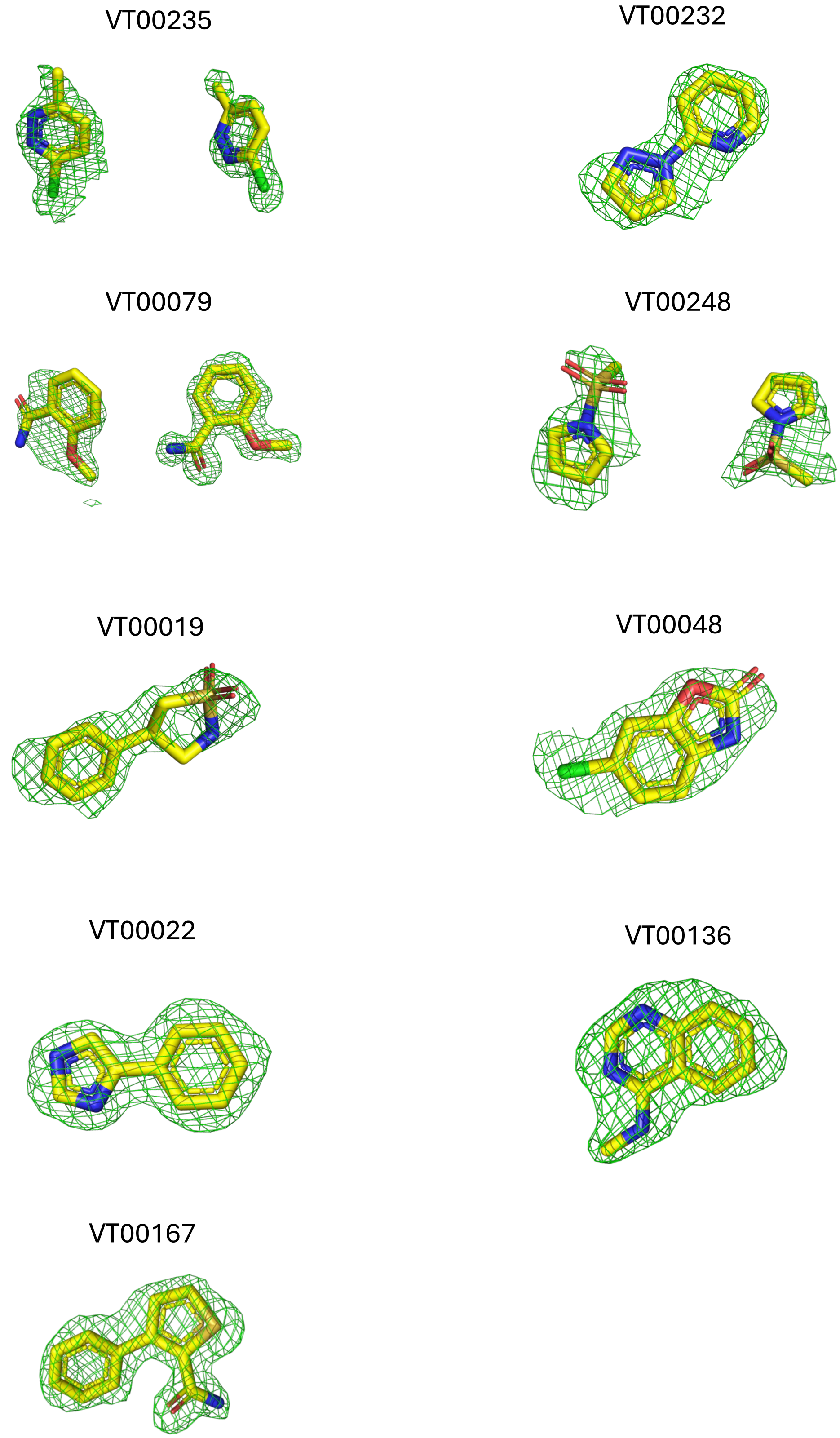

Figure S3 Fragment ID and omit maps for crystallographic fragment screening results for amidase U11 pockets that fragments bind are written. mFo-DFc omit maps are depicted as green mesh and countered at 3.0 σ level.

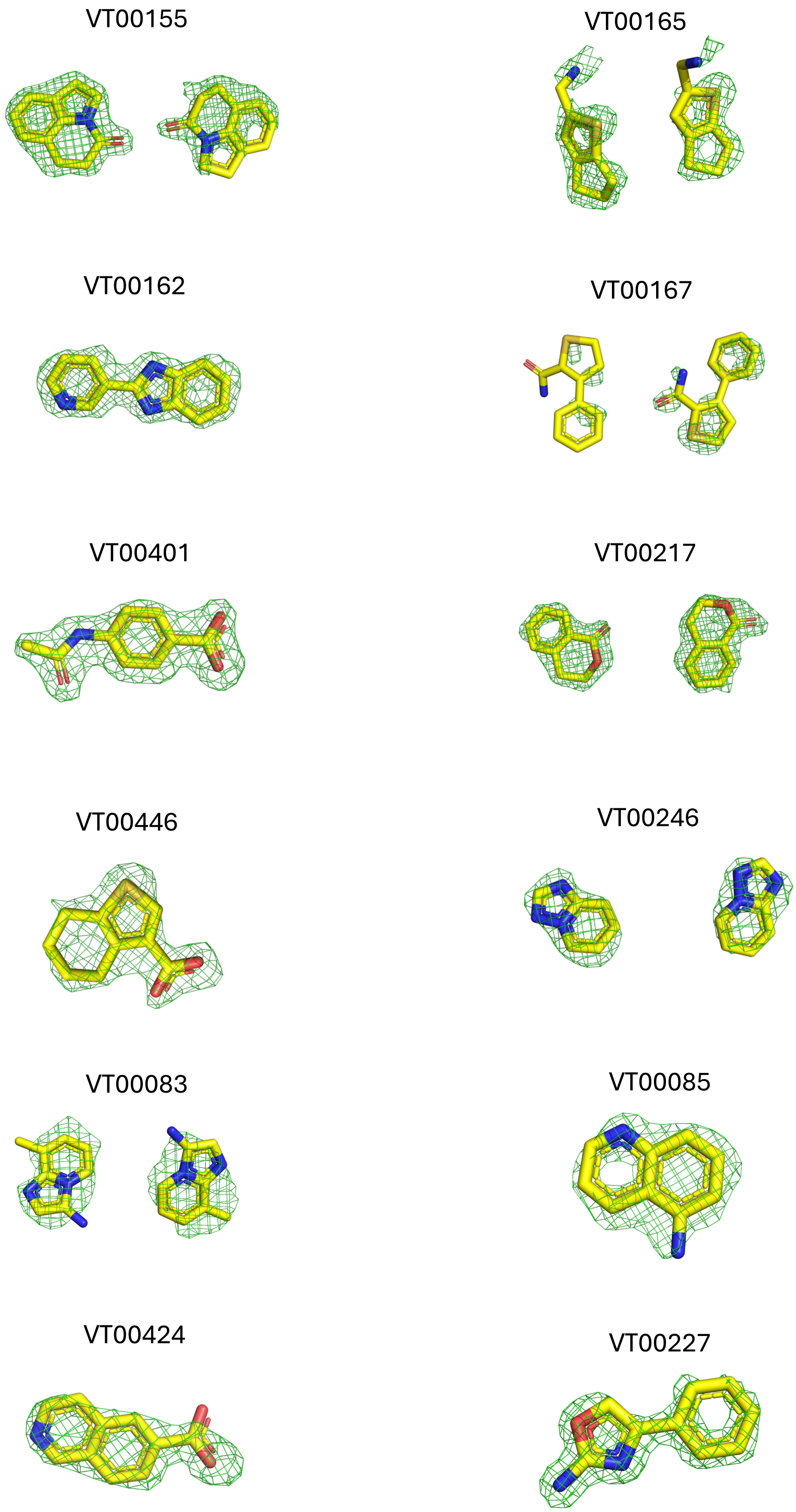

Figure S4 Fragment ID and omit maps for crystallographic fragment screening
Results for AX^{PUR5}. pockets that fragments bind are written. mFo-DFc omit maps are depicted as green mesh and countered at 3.0 σ level.

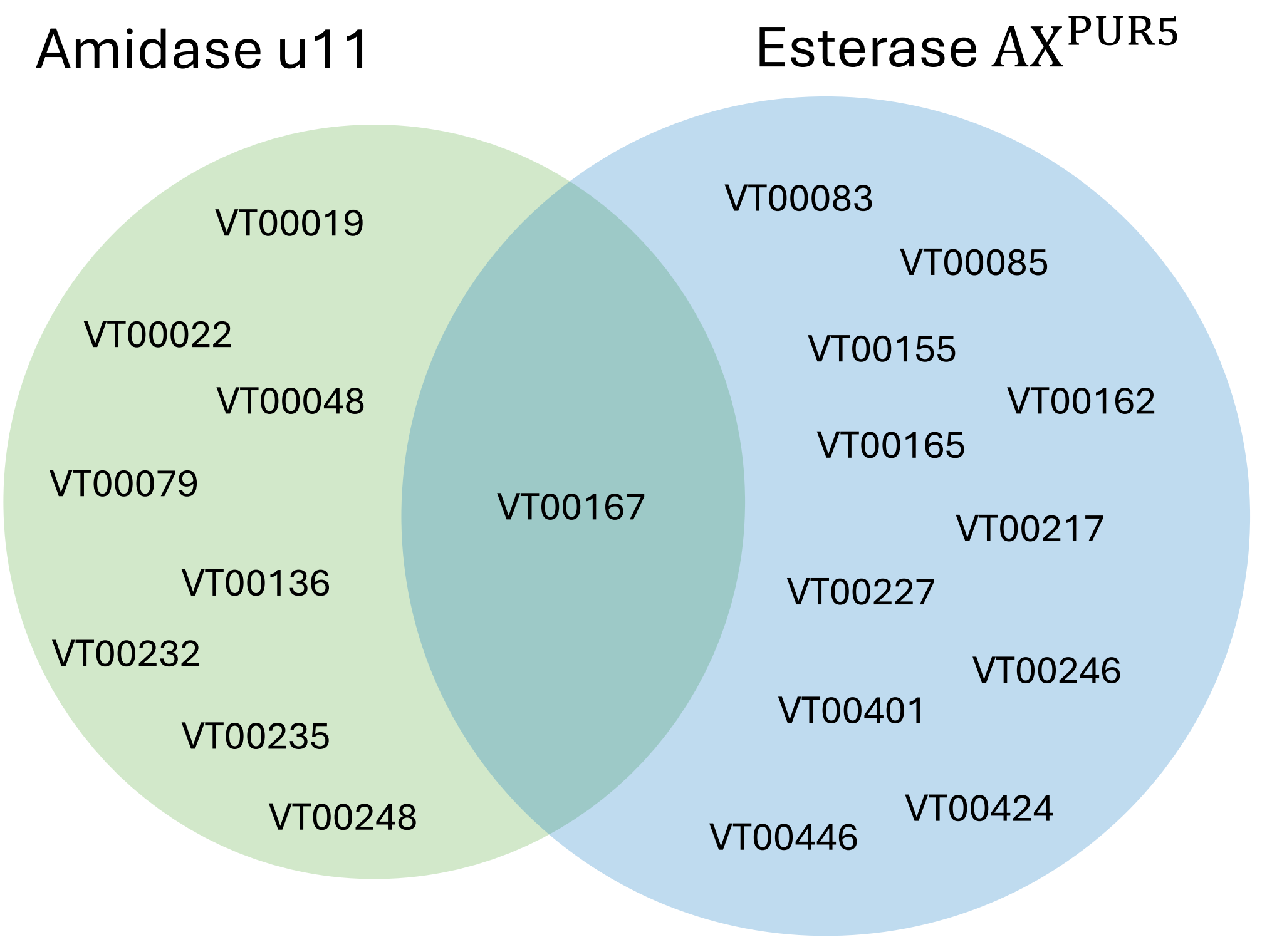

Figure S5. Distribution of fragments that bound to amidase u11 and esterase AX^{PUR5}. Only one fragment binds to both enzymes.

Table S2 PU Fragment table for name, structure and omit maps for AX^{PUR5}. Omit density maps (mFo-DFc) shown as green mesh and contoured at 3.0 σ level.

**Fragment name**	**Fragment structure**	**Omit Map**
4,4-MDA	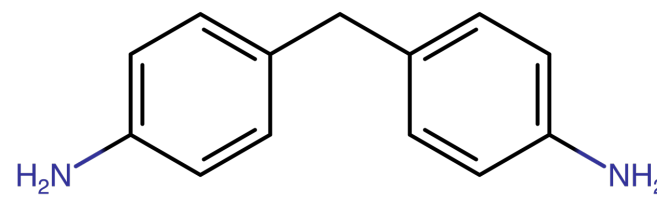	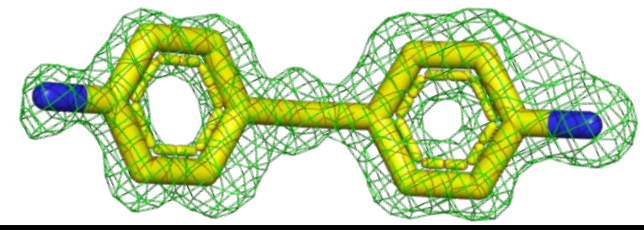
DUE-2,6-TDA	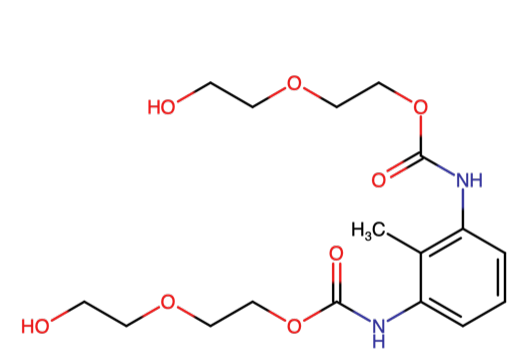	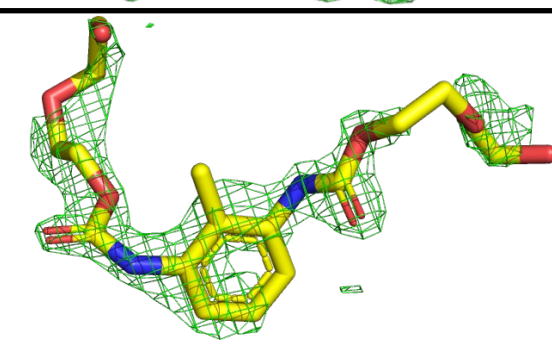
DUP-2,6-TDA	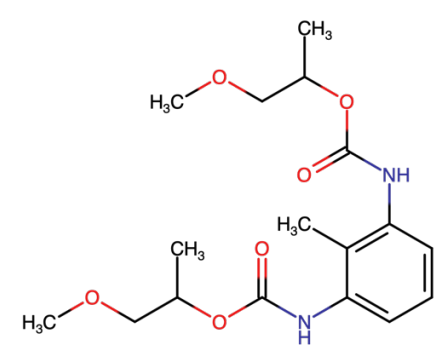	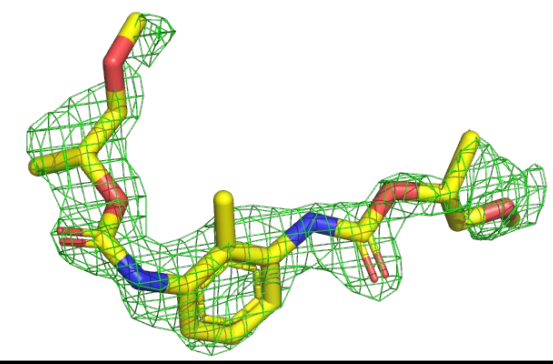
2,4-TDA (dicarbamate)	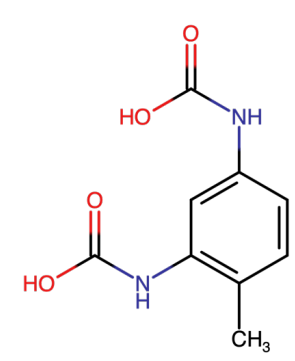	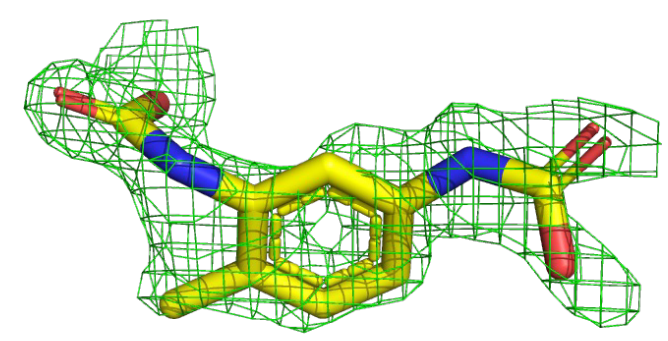
MDA-TS Analogue	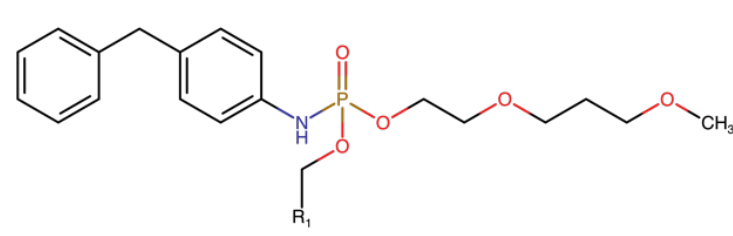	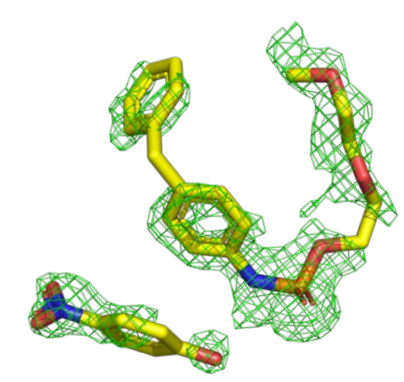
Para-nitrophenol	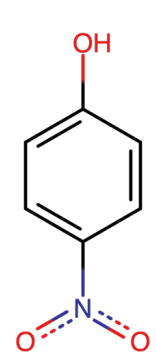	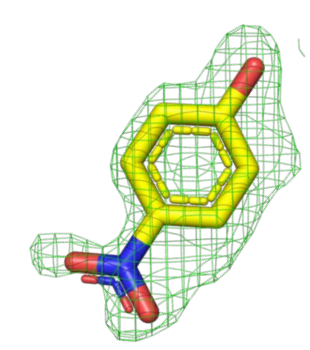
TDA-TS Analogue<br>occupancy:0.92<br>RSR: 0.15 (<0.40)<br>RSCC: 0.89 (>0.80)	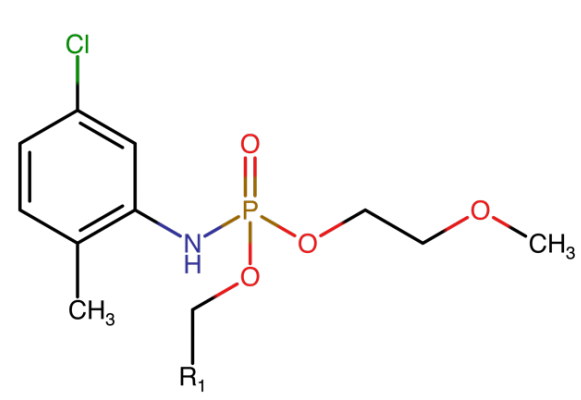	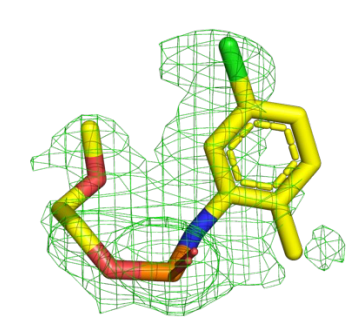
Peptide pose 1	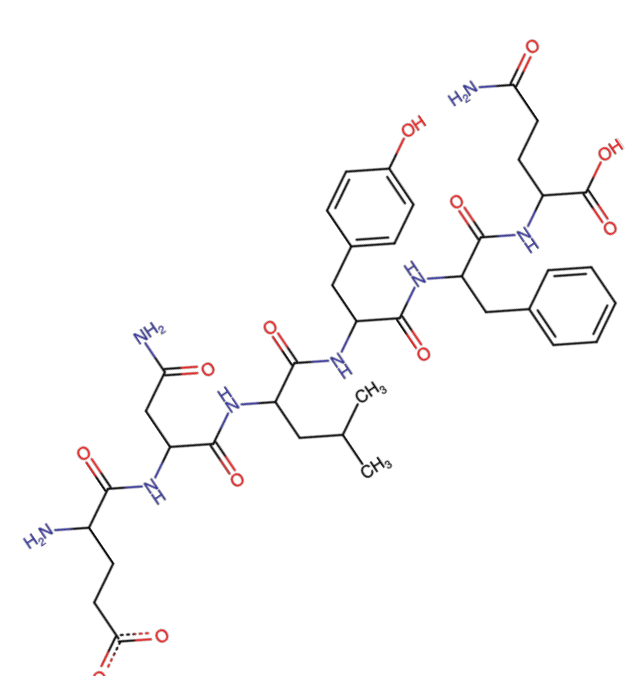	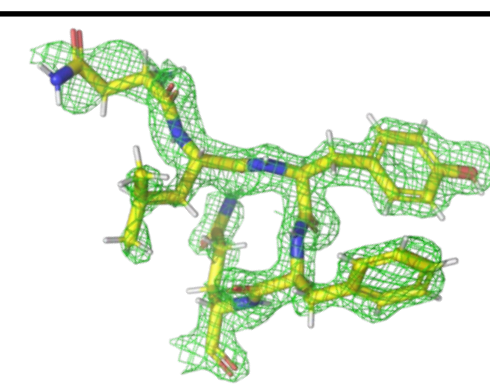
Peptide pose 2		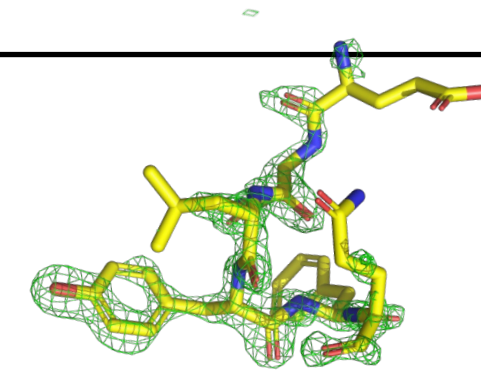

Table S3 PU Fragment table for name, structure and omit maps for u11. Omit density maps (mFo-DFc) shown as green mesh and countered at 3.0 σ level.

Fragment name	Fragment structure	Omit Map
2,4-TDA (Amine)	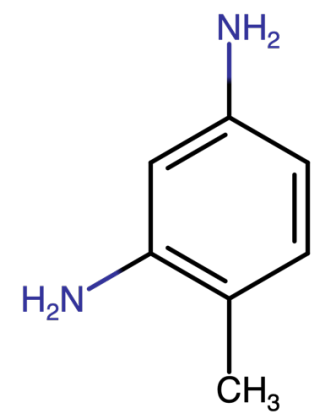	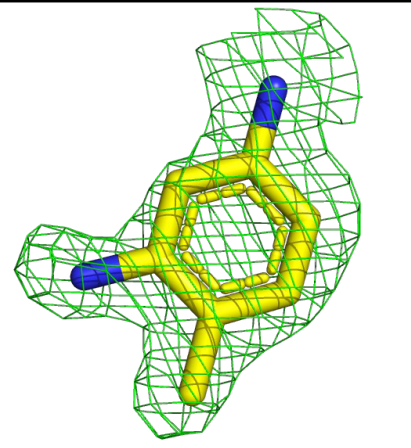
2,4-TDA (Carbamic Acid)	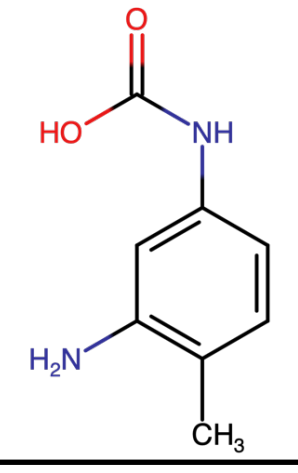	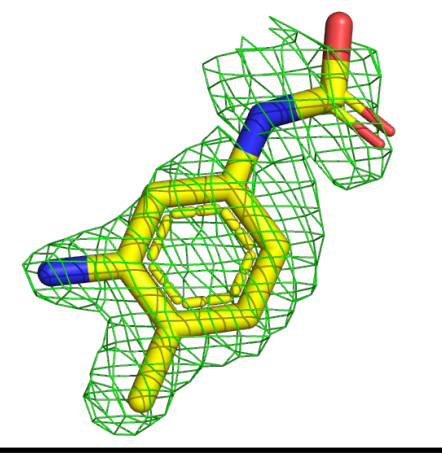
2,4-TDI	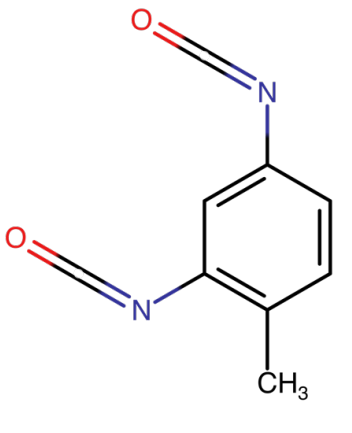	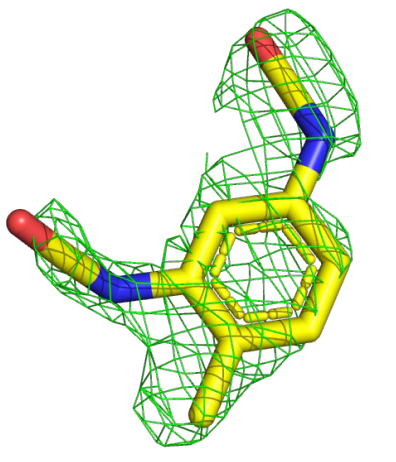
MUE-2,4-TDA	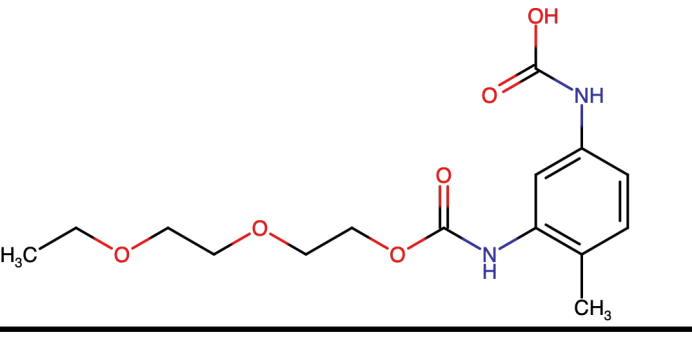	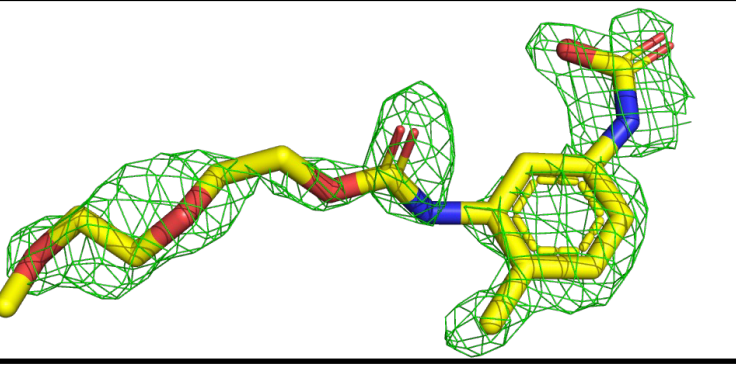
DUE-2,4-TDA	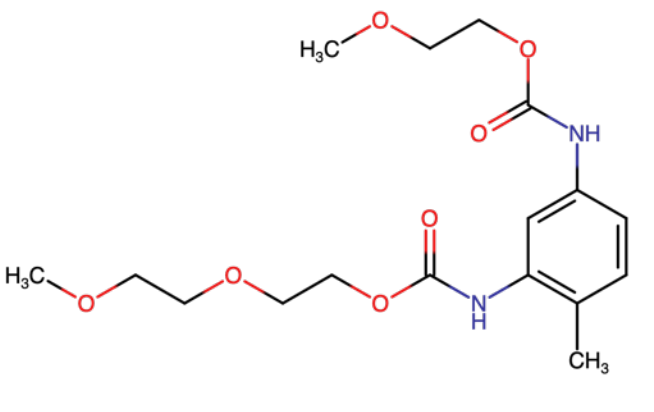	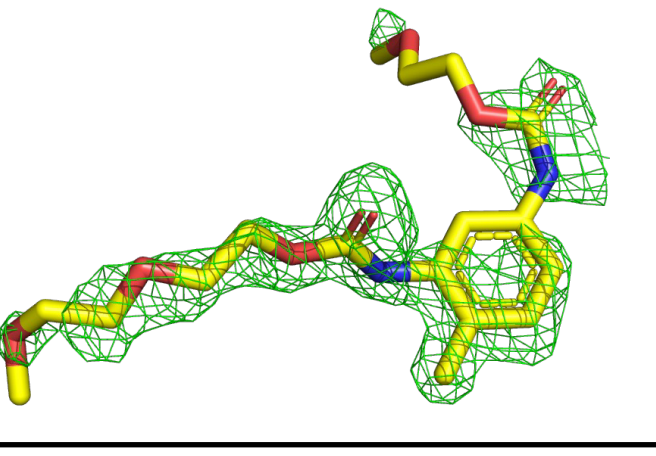
TDA TS	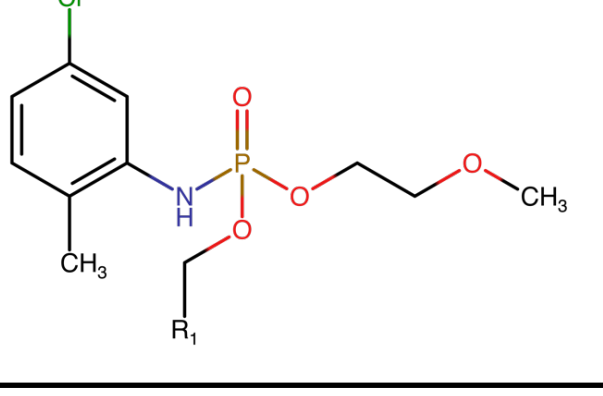	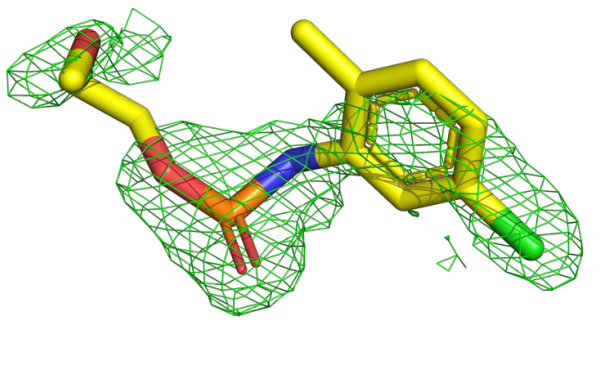
MDA TS pose 1	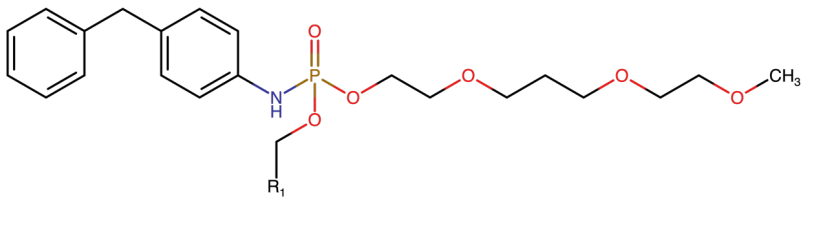	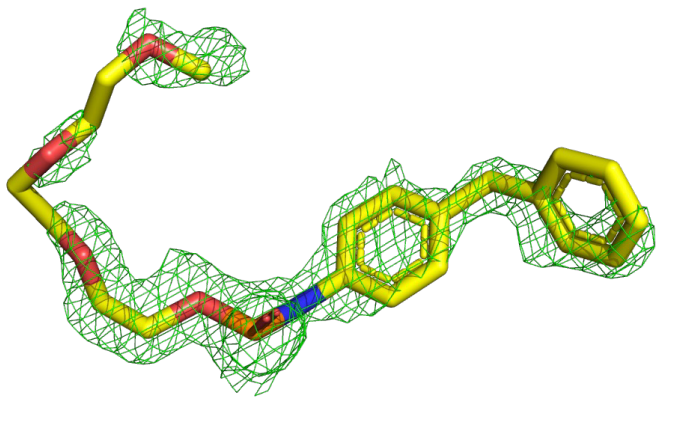
MDA TS pose 2<br><br>occupancy: 0.86<br>RSCC: 0.83 (>0.80)<br>RSR: 0.20 (<0.40)		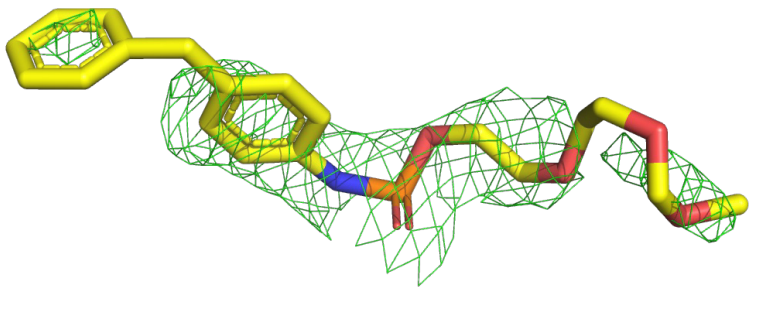

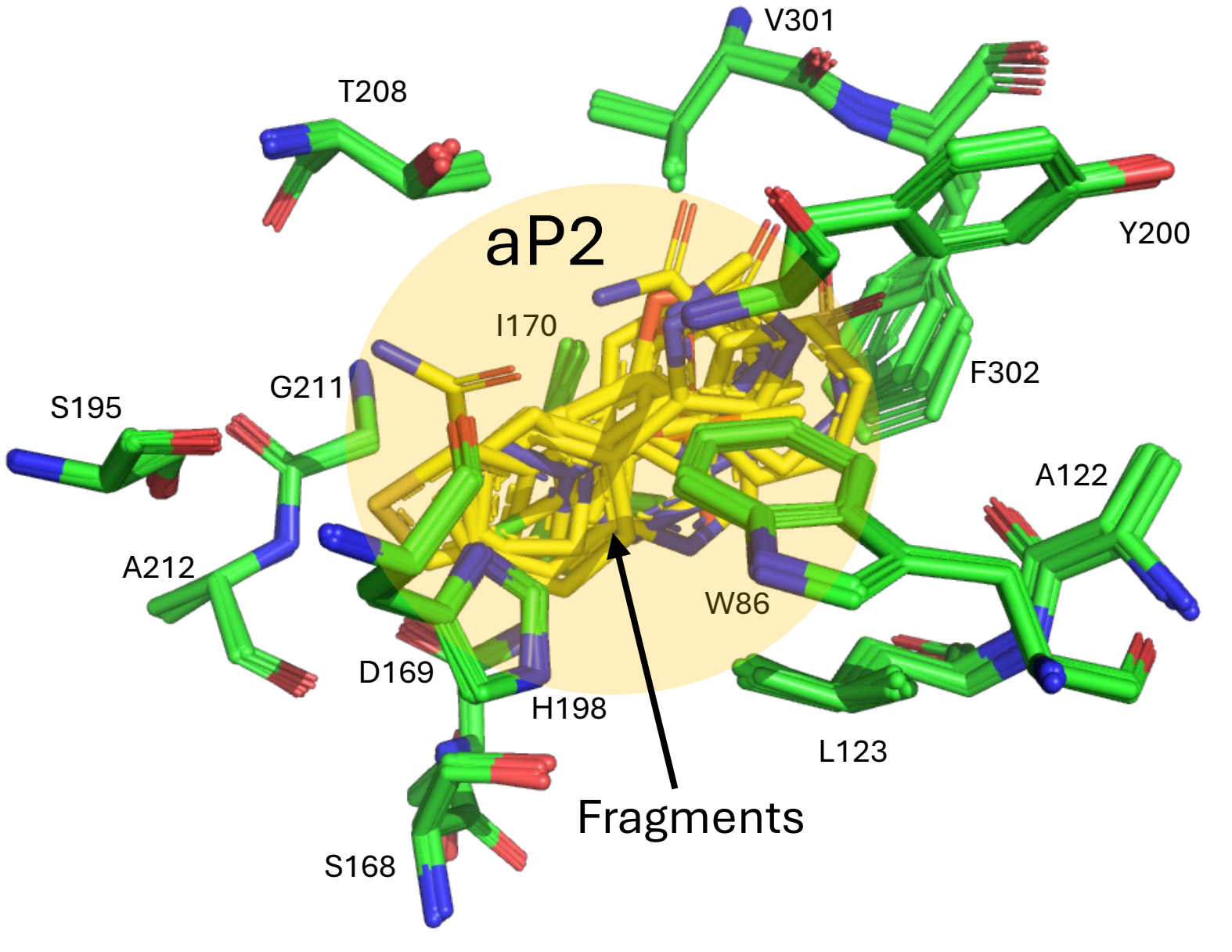

Figure S6 superimposed structures of u11 ligand free and fragments bound structures with surrounding residues depicted as a stick.

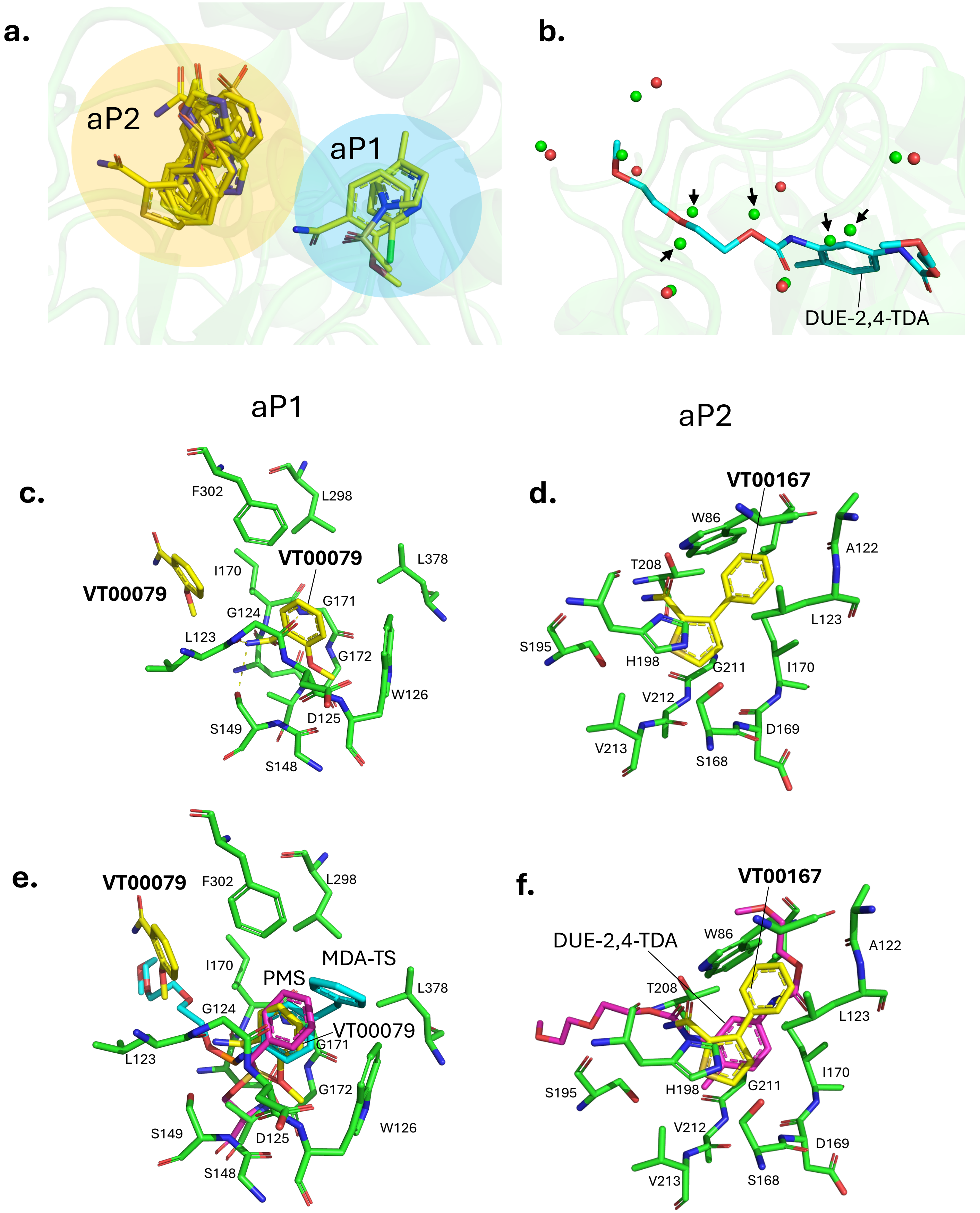

Figure S7 Selected fragments bound to the aP1 and the aP2 in amidase u11.a) close view of superposed structures of fragments in aP1 and aP2. b) superposed structures of ligand free and DUE-2,4-TDA bound states reveals water molecules(indicated by arrows) that are released upon fragment binding. c) Fragment VT00079 bound to aP1 and as well as aP2. e) Superposition of six-membered ring structures of PMS and MDA-TS with VT00079 at aP1. d) Fragment VT00167 bound to aP2. f) DUE-2,4-TDA bound to aP2 in which aromatic groups of both fragments are partially overlapped.

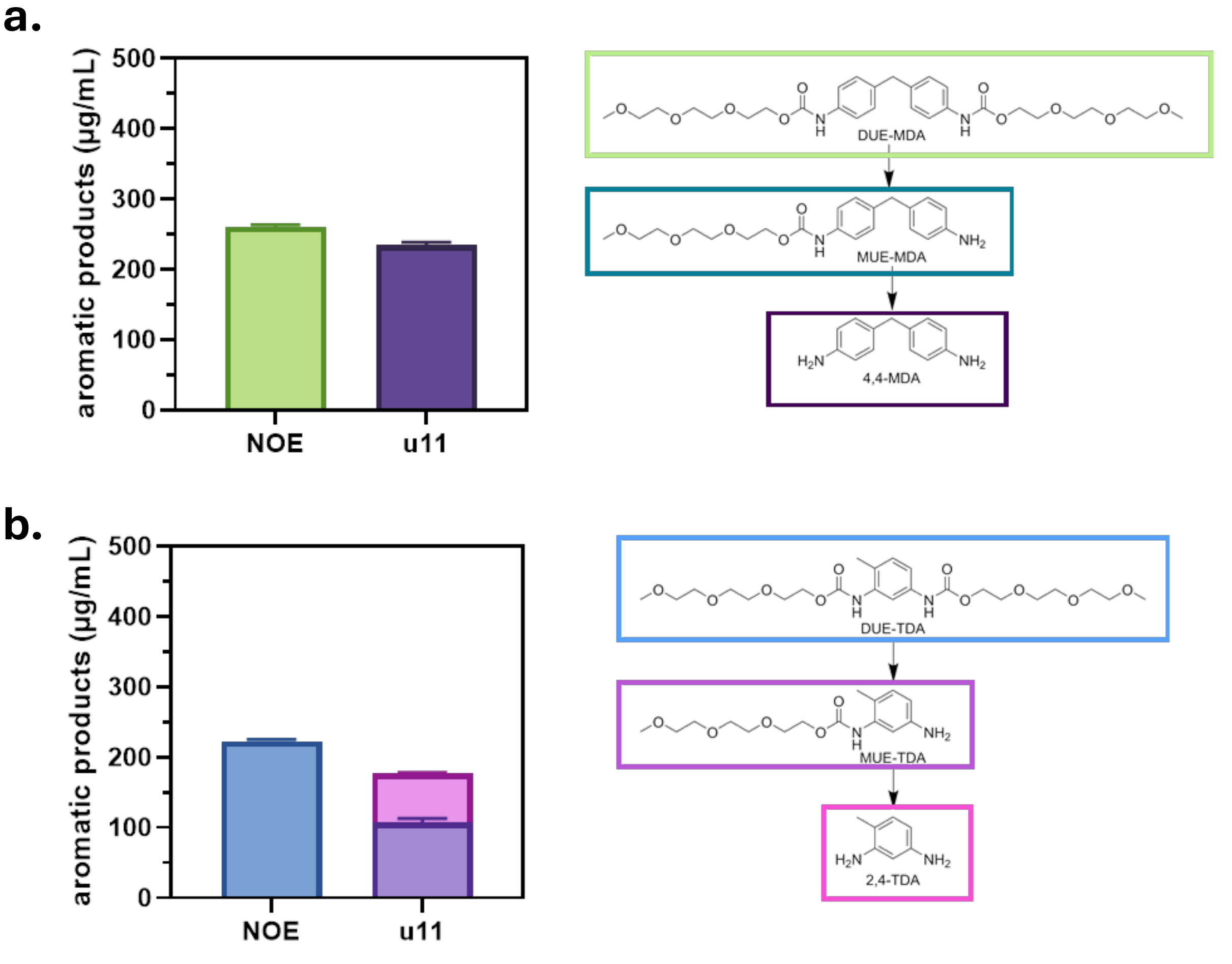

Figure S8 HPLC analysis of DUE-MDA & DUE-TDA degradation by u11. a) DUE-MDA b) DUE-TDA

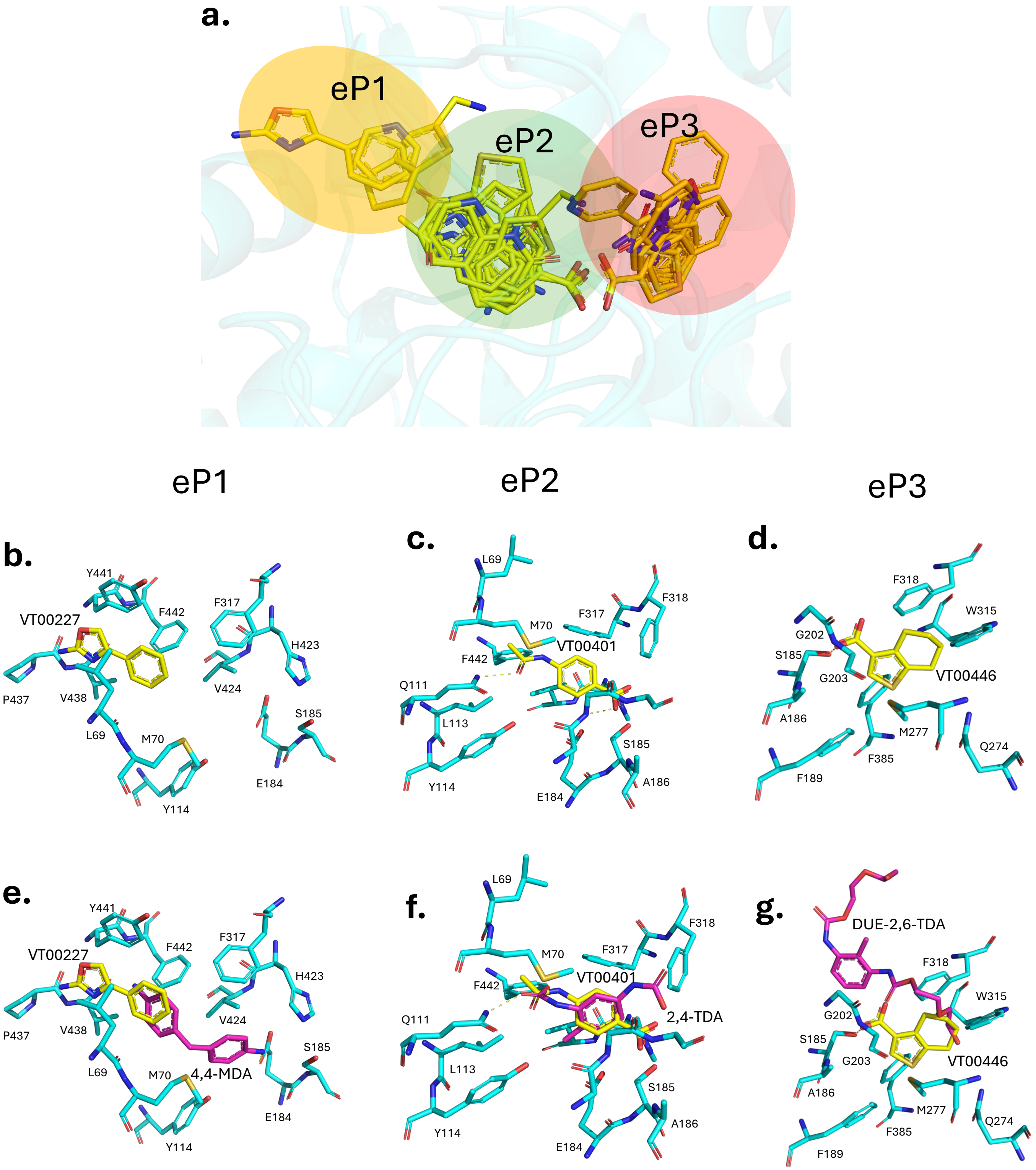

Figure S9 Selected fragments bound to eP1, eP2 and eP3. a) close view of superposed structures of fragments in eP1, eP2 and eP3. The fragment VT00227 mapped the eP1 site in b) and in e) second benzylic ring of 4,4-MDA and VT00227 partially superimposed. c) shows the fragment VT00401 bound to eP2 and make hydrogen bonding interaction with surrounding residues. f) eP2 is also targeted by 2,4-TDA fragment in which aromatic groups of VT00401 and 2,4-TDA occupy the same site, and polar groups probed the same hydrogen bonding interaction with Gln111. In d) the fragment VT00446 probed the eP3 and ethylene glycol tail of DUE-2,6-TDA partially occupy the eP3 in g).

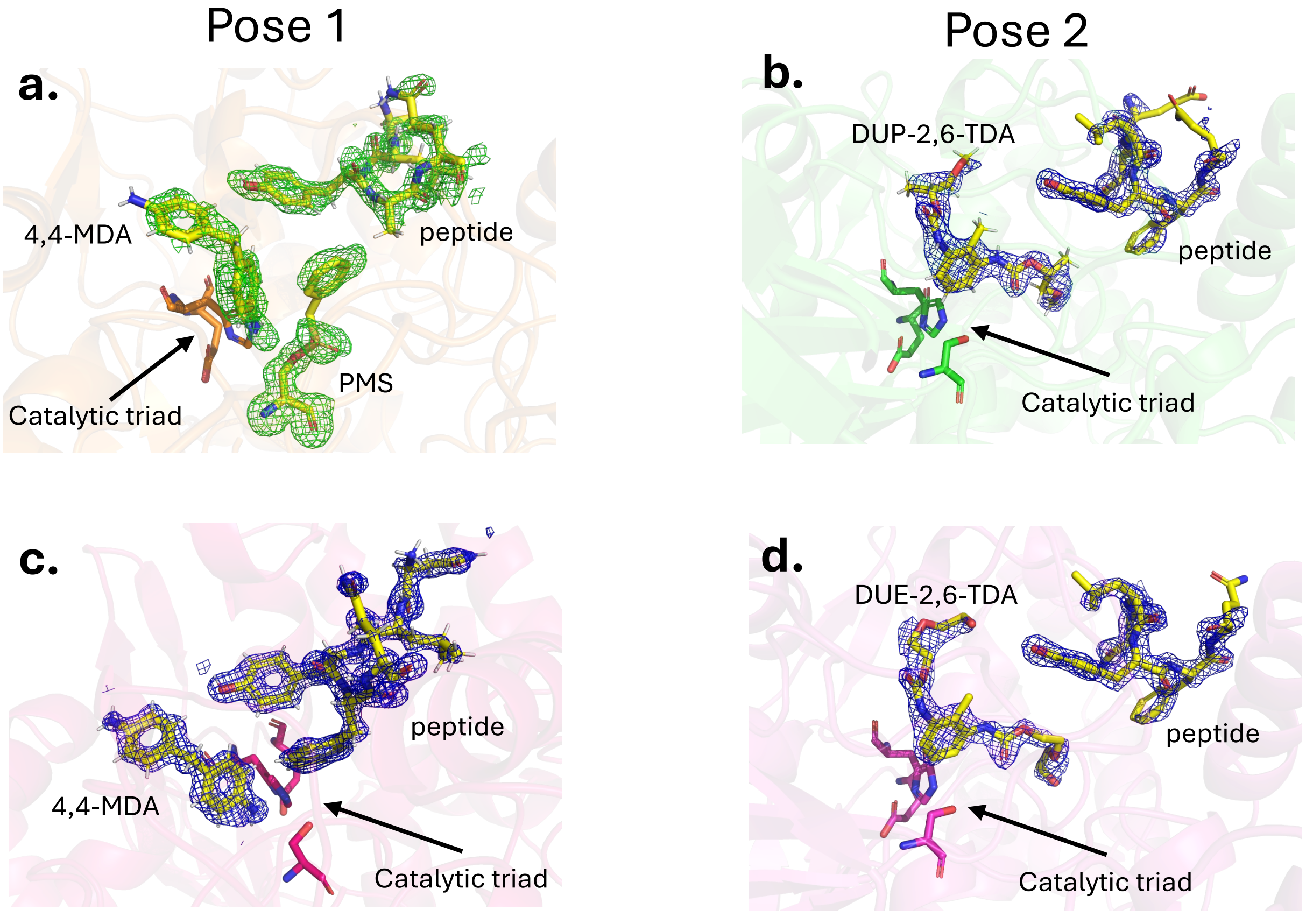

Figure S10 Change in TEV peptide conformation upon fragment binding. a) pose-1 of triple ligand bound state (PDB: 30KJ) which include 4,4-MDA (occupancy: 0.61), PMS (occupancy: 0.80) and peptide fragment (occupancy: 0.89). Omit difference mFo-DFc map at 3.0 σ contour level. b) pose-2 of peptide in DUP-2,6-TDA bound state. 2mFo-DFc map at 1.0 σ contour level c) pose-1 of peptide in 4,4-MDA bound state. 2mFo-DFc map at 1.0 σ contour level. d) b) pose-2 of peptide in DUE-2,4-TDA bound state. 2mFo-DFc map at 1.0 σ contour level

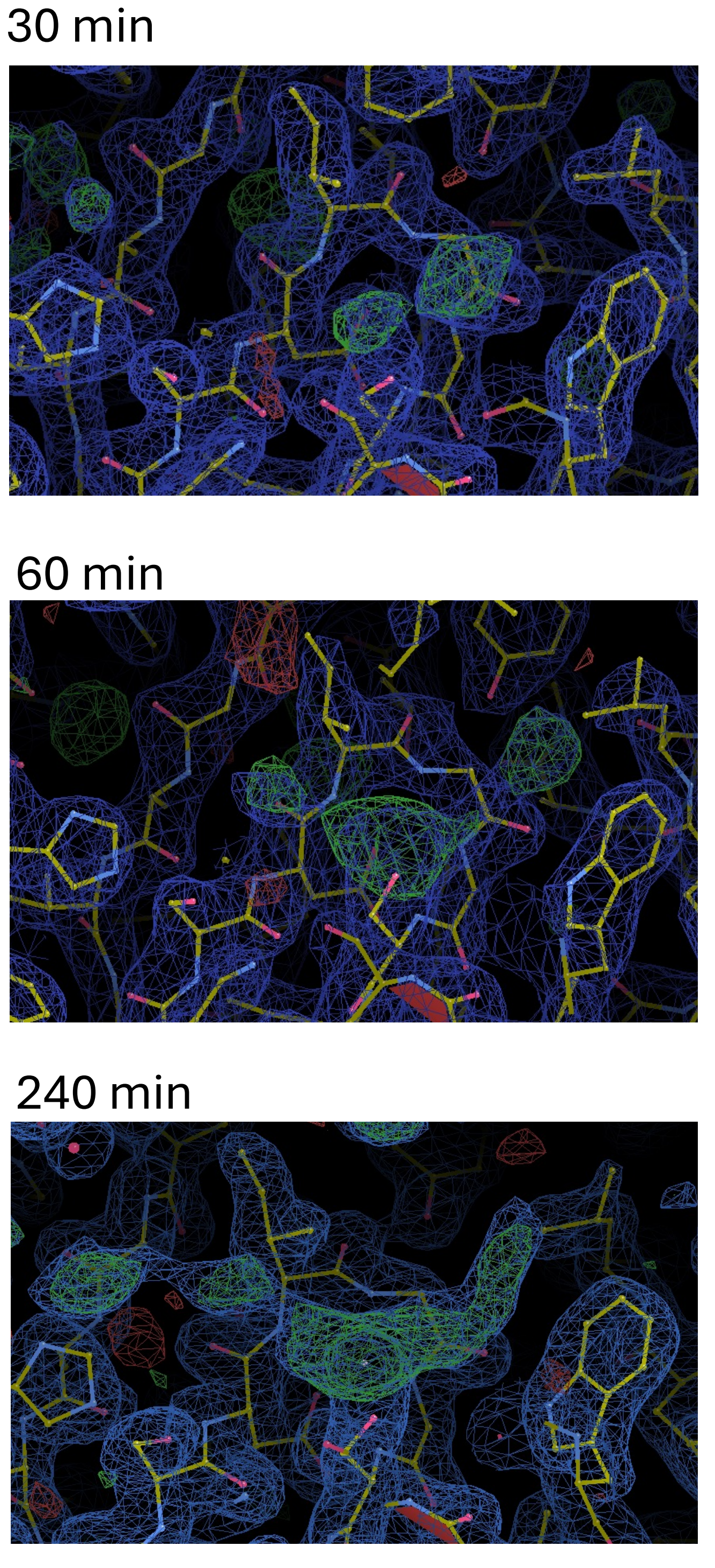

Figure S11 Appearance of positive difference peaks for MDA-TS analogue in u11 for different soaking times. 2mFo-DFc maps(blue mesh) are countered at 1.5σ counter level and mFo-DFc maps (green for positive and red for negative peaks) are countered at 3.0σ counter level.

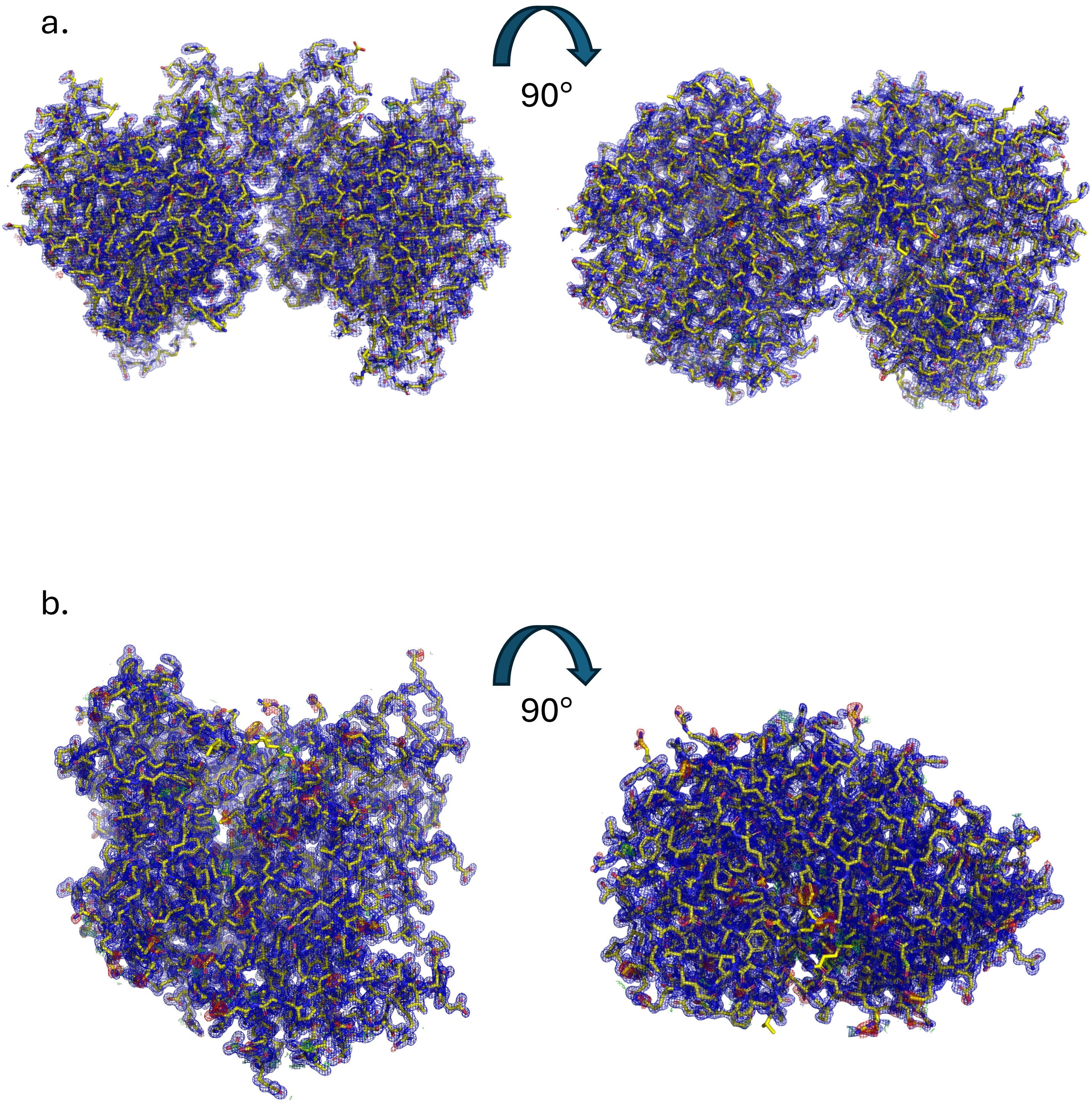

Figure S12 Electron density maps for a) u11(1.80 A) 2mFo-DFc map (blue mesh) at 1.5σ contour level and mFo-DFc map (green/red mesh) $\pm 3\sigma$ contour level at 3.0 and b) AX^{PUR5} (1.17 A) 2mFo-DFc map (blue mesh) at 1.0σ contour level and mFo-DFc map (green/red mesh) $\pm 3\sigma$ contour level at 3.0 .

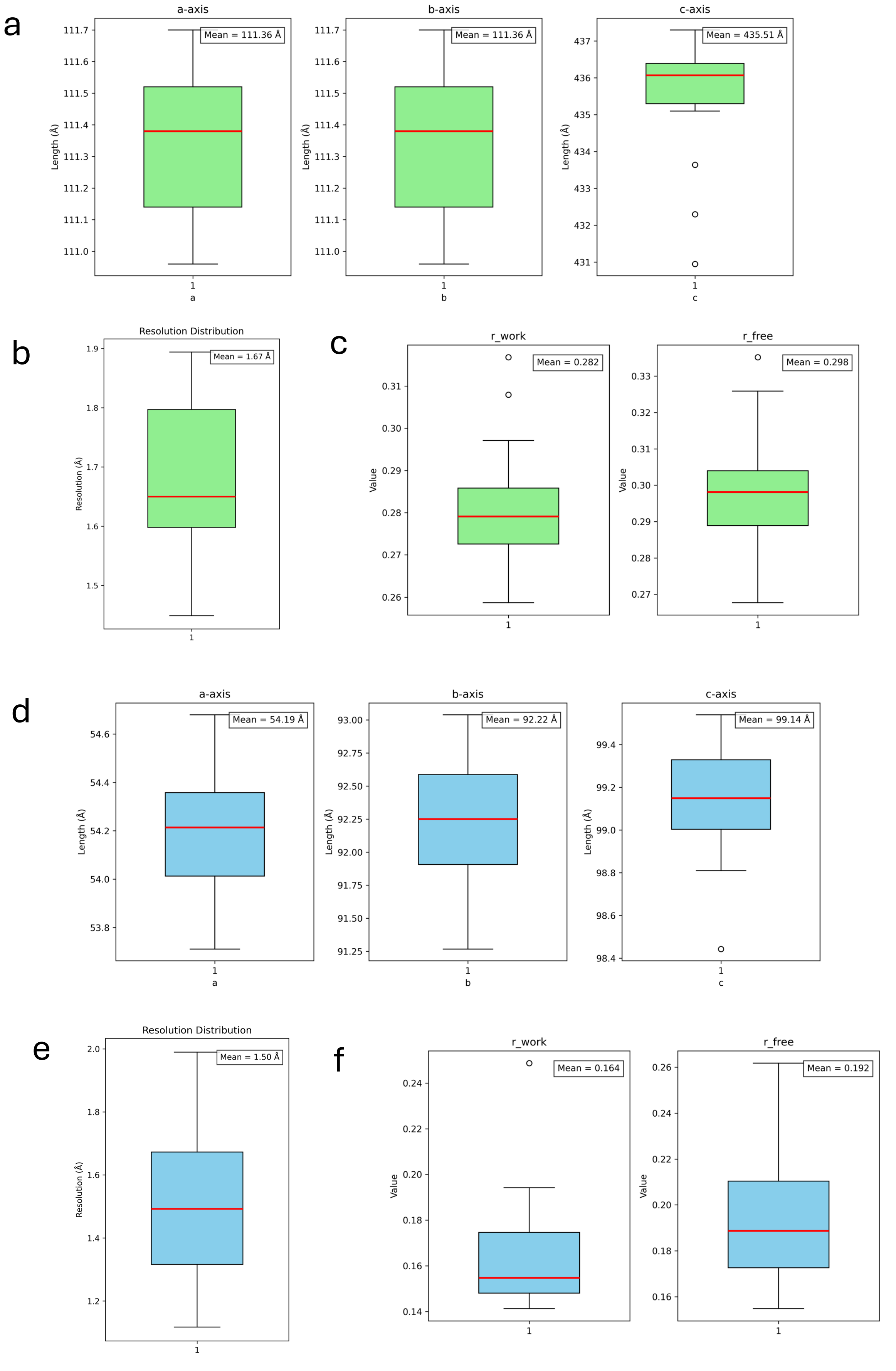

Figure S13 Statistical analysis of multi crystal data sets for distribution of unit cell dimensions, resolution, and R-factors. 22 data sets for AX^{PUR5} in a), b) and c) and 17 data sets (three of them are excluded due to different space group) for u11 in d), e) and f).

Table S4. amidase U11 x-ray diffraction data collection, processing and refinement statistics. Parenthesis for outer shell.

	crystal form 1	crystal form 2	PMSF	2,4-TDA (Carbamate)	2,4-TDA (Amine)	2,4-TDI	DUE-TDA	MUE-TDA	MDA-TS-pose 1	MDA-TS-pose 2
**Data collection**										
PDB code	29ZK	30SD	30FO	30HG	30RG	30QW	30HF	30NQ	30GE	30GV
Resolution range (Å)	49.42-2.497 (2.53-2.497)	49.58-1.894 (1.926-1.894)	49.65-1.799 (1.82-1.799)	49.62-1.599(1.62-1.599)	49.46-1.696 (1.72-1.696)	49.45-1.80 (1.82-1.80)	49.55-1.647 (1.67-1.647)	49.39-1.797 (1.82-1.797)	49.68-1.5 (1.52-1.50)	48.46-1.80 (1.82-1.80)
Space group	R32	$P6_122$	$P6_122$	$P6_122$	$P6_122$	$P6_122$	$P6_122$	$P6_122$	$P6_122$	$P6_122$
Cell dimensions a,b,c (Å) α,β,γ (°)	228.73 228.73 263.08 90 90 120	111.36 111.36 435.36 90 90 120	111.52 111.52 436.37 90 90 120	111.49 111.49 435.3 90 90 120	111.14 111.14 433.64 90 90 120	110.96 110.96 436.07 90 90 120	111.5 111.5 432.3 90 90 120	111.12 111.12 430.95 90 90 120	111.6 111.6 436.3 90 90 120	110.96 110.96 436.07 90 90 120
Total reflections	954597(68157)	2420439(157138)	4227392(142580)	7718947(147583)	3525858(234831)	5910349(444513)	6800165(373261)	5617451(411011)	9346570(367884)	5909110(421623)
Unique reflections	91093(6563)	127711(9263)	149042(10576)	399791(14661)	175296(12685)	147454(10743)	191270(13461)	146618(10512)	255729(18558)	147052(10167)
⟨I/σ(I)⟩	9.00(1.12)	15.62(1.43)	18.78(1.44)	24.70(1.80)	18.05(1.18)	23.11(2.82)	18.94(2.76)	17.96(2.72)	29.92(1.39)	17.87(1.99)
Wilson B-factor	47.71	31.66	28.47	20.82	28.95	25.53	21.56	24.8	23.39	26.77
R-meas	25.4(241.9)	13.6(221.5)	12.6(193.5)	8.20(141)	11.2(247.5)	16.4(209.4)	14.6(141.6)	23.0(188.2)	7.7(205.2)	19.7(298.1)
CC1/2 (%)	99.5(40.7)	99.9(53.9)	99.9(41.6)	100(48.4)	99.9(37.4)	99.9(59.9)	99.9(63.7)	99.9(60.5)	100.0(57.4)	99.9(41.9)
Completeness	99.8(97.4)	99.9(100.0)	99.8(97.9)	99.92(98.55)	99.9(99.2)	100.0(100.0)	99.7(96.3)	99.9(98.7)	100.0(99.7)	99.6(94.6)
Multiplicity	10.48(10.39)	18.95(17.0)	28.36(13.48)	19.3(10.1)	20.1(18.5)	40.08(41.37)	35.55(27.73)	38.31(39.1)	36.5(19.8)	40.2(41.5)
**Refinement**										
Reflections used in refinement	91011(3080)	127427(4159)	148843(4633)	210444(7616)	175020(5643)	147400(4823)	190982(5794)	146603(5199)	255429(8296)	146744(4238)
Rwork/Rfree	0.2166(0.3647)/ 0.2401(0.3725)	0.3079(0.3486)/ 0.3259(0.3973)	0.2830(0.3438)/ 0.2929(0.3537)	0.2646(0.2991)/ 0.2776(0.3382)	0.2726(0.3520)/ 0.2854(0.3670)	0.2807(0.3066)/ 0.2889(0.3243)	0.2701(0.3125)/ 0.2910(0.3507)	0.2587(0.3179)/ 0.2677(0.2931)	0.2822(0.3296)/ 0.2995(0.3777)	0.3168(0.3875)/ 0.3352(0.4257)
Number of non-hydrogen atoms	12737	6778	6788	6956	6876	6732	6838	6877	7141	
macromolecules	12675	6252	6312	6244	6252	6250	6235	6286	6252	
ligands	0	10	0	46	20	48	74	63	76	
solvent	62	516	476	666	604	434	529	528	813	
RMS(bonds) (Å)	0.003	0.002	0.006	0.007	0.004	0.002	0.003	0.003	0.008	
RMS(angles) (°)	0.7	0.49	0.95	0.86	0.66	0.57	0.61	0.67	0.93	
Ramachandran favored (%)	97.81	98.09	97.37	97.73	97.37	97.49	97.37	97.63	97.61	
Ramachandran allowed (%)	2.13	1.91	2.63	2.27	2.63	2.51	2.63	2.25	2.39	
Ramachandran outliers (%)	0.06	0	0	0	0	0	0	0.12	0	
Clashscore	3.29	0	2.15	0.79	0.32	0.32	0.47	1.26	0.31	
Average B-factor	53.82	30.01	31.52	23.67	30.26	26.72	24.56	26.9	26.17	
macromolecules	53.88	30.44	31.57	22.96	29.76	26.44	24.1	26.47	25.08	
ligands		22.79		29.31	32.5	33.42	32.8	35.07	31.96	
solvent	42.19	24.94	33.6	29.93	35.39	30.04	28.88	31.05	33.99	

	TDA-TS	VT00019	VT00022	VT00048	VT00079	VT00136	VT00167	VT00232	VT00235	VT00248
**Data collection**										
PDB code	30TU	30OC	30OM	30OP	30PJ	30PL	30OB	30QO	30QP	30QY
Resolution range (Å)	48.09-1.64 (1.66-1.64)	49.65-1.549 (1.57-1.549)	49.68-1.60 (1.62-1.60)	49.56-1.696 (1.71-1.696)	49.7-1.449 (1.47-1.449)	49.49-1.598 (1.62-1.598)	49.55-1.549 (1.57-1.549)	49.71-1.649 (1.67-1.649)	48.56-1.65 (1.67-1.65)	48.23-1.786 (1.81-1.786)
Space group	$P6_222$	$P6_122$	$P6_222$	$P6_122$	$P6_122$	$P6_122$	$P6_122$	$P6_122$	$P6_122$	$P6_122$
Cell dimensions a,b,c (Å) α,β,γ (°)	111.05 111.05 217.07 90 90 120	111.5 111.5 436.6 90 90 120	111.55 111.55 218.41 90 90 120	111.26 111.26 436.19 90 90 120	111.6 111.6 437.1 90 90 120	111.1 111.1 435.7 90 90 120	111.3 111.3 435.1 90 90 120	111.6 111.6 437.3 90 90 120	111.7 111.7 437 90 90 120	111.38 111.38 436.39 90 90 120
Total reflections	3874321(382547)	8823682(407510)	4112419(105864)	6977248(486200)	9831131(320914)	8166951(430028)	8749367(398593)	7405561(351868)	3085718(119177)	4054476(433785)
Unique reflections	97133(9404)	227626(13924)	219049(7602)	176622(12680)	283318(20391)	209563(15067)	230493(16719)	189700(11401)	193521(13978)	150453(22642)
⟨I/σ(I)⟩	20.9(1.1)	29.08(3.09)	28.05(1.39)	22.81(2.52)	24.92(1.02)	28.21(2.06)	25.47(1.27)	36.74(4.55)	19.03(1.83)	10.40(1.53)
Wilson B-factor	28.43	22.1	31.94	25.8	20.7	24.47	25.53	20.67	20.92	22.03
R-meas	13.2(501.5)	8.6(120.7)	7.4(244.8)	11.8(166.0)	9.3(227.0)	8.8(176.4)	9.6(262.3)	8.3(100.7)	9.8(116.2)	24.2(103.0)
CC1/2 (%)	100.0(56.0)	100.0(77.0)	99.9(61.3)	100.0(75.8)	100.0(42.5)	100.0(67.9)	100.0(47.8)	100.0(81.5)	99.9(53.8)	99.4(56.0)
Completeness	100.0(100.0)	98.1(82.6)	99.8(98.2)	99.8(98.2)	99.9(98.7)	99.9(98.6)	100.0(99.6)	97.9(80.9)	99.9(98.9)	98.9(93.8)
Multiplicity	39.9(40.7)	38.8(29.3)	38.8(28.8)	39.5(38.3)	34.7(15.7)	38.97(28.5)	38.0(23.8)	39.0(30.9)	16.0(8.5)	27.0(19.2)
**Refinement**										
Reflections used in refinement	96927(3222)	227329(19289)	105738(10289)	175406(17267)	282765(27808)	208964(20613)	229949(22598)	189374(15885)	193267(18864)	149944(14063)
Rwork/Rfree	0.3187(0.4085)/ 0.3312(0.4328)	0.2783(0.2891)/ 0.2996(0.3247)	0.2466(0.3256)/ 0.2680(0.3470)	0.2886(0.2798)/ 0.3054(0.3107)	0.2858(0.3285)/ 0.3040(0.3381)	0.2791(0.2939)/ 0.2981(0.3213)	0.2679(0.3088)/ 0.2791(0.3361)	0.2782(0.2632)/ 0.3038(0.2990)	0.2765(0.2953)/ 0.2952(0.3150)	0.2971(0.3186)/ 0.3231(0.3644)
Number of non-hydrogen atoms	6606	3328	7135	3367	7000	7249	7058	6978	6962	6970
macromolecules	6280	3126	6250	3125	6250	6250	6252	6250	6252	6250
ligands	59	27	57	12	32	82	44	48	58	74
solvent	267	175	853	230	726	953	780	698	666	666
RMS(bonds) (Å)	0.003	0.003	0.077	0.078	0.026	0.111	0.083	0.073	0.004	0.069
RMS(angles) (°)	0.69	0.69	1.7	1.86	1.75	1.93	1.99	1.81	0.65	0.59
Ramachandran favored (%)	97.98	96.66	97.49	97.37	97.85	97.73	97.49	97.61	97.73	97.37
Ramachandran allowed (%)	2.02	3.24	2.51	2.63	2.15	2.27	2.51	2.39	2.27	2.63
Ramachandran outliers (%)	0	0	0	0	0	0	0	0	0	0
Clashscore	3.26	2.86	1.2	2.08	3.93	3.63	3.2	3.04	3.27	3.75
Average B-factor	28.06	30.9	24.82	34.78	26.98	23.9	26.69	28.56	22.46	22.24
macromolecules	27.91	30.79	23.77	34.48	26.19	22.59	25.39	27.73	21.71	21.55
ligands	35.41	34.44	29.14	29.15	35.94	27.74	28.39	29.86	29.97	28.89
solvent	30.08	32.24	32.35	39.13	33.45	32.27	33.49	35.49	29.06	28.23

Number of non-hydrogen atoms	6970
macromolecules	6246
ligands	58
solvent	666
RMS(bonds) (Å)	0.003
RMS(angles) (°)	0.6
Ramachandran favored (%)	97.01
Ramachandran allowed (%)	2.99
Ramachandran outliers (%)	0
Clashscore	2.31
Average B-factor	22.61
macromolecules	22.12
ligands	28.46
solvent	26.77

Table S5. esterase AX^{PUR5} x-ray diffraction data collection, processing and refinement statistics. Parenthesis for outer shell.

	Apo	PMSF	2,4-TDA	DUE-2,6-TDA	DUP-2,6-TDA	4,4-MDA	MDA-TS	TDA-TS	p-nitrophenol	Triple
**Data collection**										
PDB code	29JV	29JY	29NV	29OJ	29OO	29KX	30TV	30VA	29NW	29KJ
Resolution range (Å)	36.495-1.298 (1.345-1.298)	41.851-1.298 (1.344-1.298)	33.281-1.168 (1.210-1.168)	47.67-1.524 (1.54-1.524)	47.6-1.564 (1.58-1.564)	34.009-1.117 (1.157-1.117)	24.840-1.442 (1.494-1.442)	67.623-1.386 (1.410-1.386)	47.53-1.167 (1.18-1.167)	47.65-1.248 (1.26-1.248)
Space group	$P2_12_12_1$	$P2_12_12_1$	$P2_12_12_1$	$P2_12_12_1$	$P2_12_12_1$	$P2_12_12_1$	$P2_12_12_1$	$P2_12_12_1$	$P2_12_12_1$	$P2_12_12_1$
Cell dimensions a,b,c (Å) α,β,γ (°)	54.05 92.79 98.95 90 90 90	54.38 92.40 98.81 90 90 90	54.39 92.89 99.39 90 90 90	54.32 92.302 99.347 90 90 90	54.238 92.127 99.266 90 90 90	54.288 92.162 99.066 90 90 90	54.68 93.04 99.36 90 90 90	54.503 92.306 99.348 90 90 90	54.19 92.6 98.92 90 90 90	54.26 92.61 99.54 90 90 90
Total reflections	1687631(110381)	1690747(112607)	2127717(85069)	1019970(74847)	960538(70159)	2209109(68092)	1240920(84012)	1072374(34918)	2287673(150885)	1904297(138243)
Unique reflections	123217(8999)	123342(8975)	169971(11901)	76833(5509)	71047(5157)	187235(11371)	91681(6486)	102129(4971)	167962(12074)	139636(10184)
⟨I/σ(I)⟩	16.12(1.73)	14.74(1.51)	19.27(1.96)	17.1(3.2)	19.4(3.1)	20.9(2.5)	16.48(1.72)	10.5(1.0)	25.41(3.68)	15.75(1.24)
Wilson B-factor	14.78	14.94	14.88	17.43	18.3	12.76	14.51	17.05	14.27	15.87
R-meas	8.7(118.0)	9.3(135.1)	6.2(104.5)	10.0(93.1)	8.9(93.4)	5.6(68.3)	16.5(249.5)	11.4(251.3)	4.8(72.9)	8.5(222.0)
CC1/2 (%)	100.0(73.2)	100.0(75.2)	99.9(77.4)	99.9(87.7)	99.9(87.2)	99.9(80.7)	99.9(63.4)	99.9(30.8)	99.9(91.5)	99.9(54.2)
Completeness	100.0(99.7)	100.0(99.7)	99.6(95.1)	99.8(98.0)	99.9(99.2)	97.6(81.8)	99.7(96.5)	99.8(98.4)	99.3(97.5)	100.0(99.9)
Multiplicity	13.7(12.3)	13.7(12.5)	12.5(7.1)	13.3(13.6)	13.5(13.6)	11.8(6.0)	13.5(13.0)	10.5(7.0)	15.2(14.0)	13.6(13.6)
**Refinement**										
Reflections used in refinement	123195(4037)	123296(4037)	169811(5257)	76726(7479)	70936(6961)	187095(10945)	91619(8803)	100783(9617)	167835(5346)	139576(4627)
Rwork/Rfree	0.1452(0.2557)/ 0.1679(0.3114)	0.1519(0.2892)/ 0.1762(0.3190)	0.1417(0.2442)/ 0.1629(0.2751)	0.1662(0.2480)/ 0.1886(0.2808)	0.1629(0.2847)/ 0.1958(0.3284)	0.1461(0.19719)/ 0.1548(0.1974)	0.1603(0.3469)/ 0.1864(0.3749)	0.1547(0.3334)/ 0.1836(0.3696)	0.1413(0.2154)/ 0.1606(0.2499)	0.1467(0.3585)/ 0.1705(0.3694)
Number of non-hydrogen atoms	4475	4461	4429	4499	4506	4399	4385	4415	4473	4500
macromolecules	4008	3970	3960	4008	4017	4008	3960	3960	4008	4018
ligands	10	17	25	37	61	25	90	54	22	25
solvent	457	474	444	454	454	366	379	429	443	457
RMS(bonds) (Å)	0.007	0.004	0.005	0.165	0.098	0.005	0.179	0.088	0.007	0.007
RMS(angles) (°)	0.99	0.83	0.91	2.23	2.4	0.87	2.99	2.55	1	1.06
Ramachandran favored (%)	96.66	97.61	97.04	97.25	96.86	96.86	97.04	96.84	97.05	97.23
Ramachandran allowed (%)	3.14	2.19	2.77	2.55	2.94	2.95	2.77	2.96	2.75	2.57
Ramachandran outliers (%)	0.2	0.2	0.2	0.2	0.2	0.2	0.2	0.2	0.2	0.2
Clashscore	1.02	1.03	1.28	1.65	3.67	1.14	2.43	1.93	2.03	2.79
Average B-factor	19.74	20.54	21.69	23.26	25.5	17.17	20.07	25.39	19.76	21.9
macromolecules	18.76	19.51	20.76	22.18	24.43	16.53	19.42	24.45	18.8	20.87
ligands	18.16	23.22	23.75	35.11	35.36	16.51	21.18	31.76	22.63	20.74
solvent	28.43	29	29.86	31.81	34.17	24.22	26.7	33.68	28.25	30.98

	VT00083	VT00085	VT00155	VT00162	VT00165	VT00167	VT00217	VT00227	VT00246	VT00401	VT00424	VT00446
**Data collection**												
PDB code	29QA	29QC	29PW	29PZ	29QE	29QX	29QU	29QS	29RV	29RS	29RW	29RU
Resolution range (Å)	47.67-1.78 (1.83-1.78)	47.44-1.53 (1.57-1.53)	47.75-1.37 (1.41-1.37)	47.55-1.59 (1.63-1.59)	47.61-1.53 (1.57-1.53)	47.15-1.7 (1.761-1.7)	47.49-1.394 (1.41-1.394)	47.39-1.46 (1.49-1.46)	47.36-1.99 (2.04-1.99)	47.39-1.85 (1.90-1.85)	67.338-1.725 (1.755-1.725)	67.365-1.784 (1.815-1.784)
Space group	$P2_12_12_1$	$P2_12_12_1$	$P2_12_12_1$	$P2_12_12_1$	$P2_12_12_1$	$P2_12_12_1$	$P2_12_12_1$	$P2_12_12_1$	$P2_12_12_1$	$P2_12_12_1$	$P2_12_12_1$	$P2_12_12_1$
Cell dimensions a,b,c (Å) α,β,γ (°)	54.370 91.737 99.114 90 90 90	54.046 91.652 99.001 90 90 90	54.463 92.798 99.254 90 90 90	54.180 92.549 99.177 90 90 90	54.275 92.327 99.122 90 90 90	53.711 91.267 98.443 90 90 90	54.08 92.2 99.2 90 90 90	53.911 91.903 99.411 90 90 90	53.945 92.113 98.917 90 90 90	53.932 91.361 99.274 90 90 90	54.002 91.788 99.090 90 90 90	53.874 91.920 99.011 90 90 90
Total reflections	635523(42344)	1004246(71014)	1422516(102585)	918258(64343)	1022701(70273)	668624(37787)	1339644(97167)	1178005(80697)	449756(26820)	574961(42609)	654037(26973)	549584(27514)
Unique reflections	48346(3475)	74136(5259)	104505(7358)	67700(4858)	75421(5281)	62333(4349)	99499(7218)	86663(5998)	34274(2283)	42729(3086)	49881(2591)	47544(2333)
⟨I/σ(I)⟩	15.6(3.2)	17.9(3.0)	19.3(3.1)	16.8(2.9)	16.6(3.0)	6.4(1.5)	13.94(1.25)	18.8(3.3)	11.0(2.3)	18.5(3.2)	9.9(2.0)	7.5(1.7)
Wilson B-factor	21.69	16.97	15.25	18.69	16.21	16.08	18.02	16.05	25.21	27.00	17.92	20.42
R-meas	11.8(84.4)	9.4(96.4)	7.6(90.6)	10.1(93.0)	10.5(101.8)	28.2(78.1)	10.0(216.6)	8.2(93.6)	21.5(101.7)	10.0(85.4)	19.8(153.5)	27.9(241.1)
CC1/2 (%)	99.9(80.8)	99.9(77.8)	99.9(88.9)	99.9(81.3)	99.9(77.4)	98.5(53.8)	99.9(56.1)	99.9(86.2)	99.5(82.4)	99.9(77.6)	99.7(34.4)	99.7(36.7)
Completeness	99.8(97.9)	99.7(96.6)	98.4(94.5)	99.4(97.7)	99.7(95.5)	99.6(94.7)	99.9(99.2)	99.6(94.6)	99.4(91.8)	99.9(99.2)	94.8(99.6)	100.0(100.0)
Multiplicity	13.1(12.2)	13.5(13.5)	13.6(13.9)	13.6(13.2)	13.6(13.3)	10.7(8.7)	13.5(13.5)	13.6(13.5)	13.1(11.7)	13.5(13.8)	13.1(10.4)	11.6(11.8)
**Refinement**												
Reflections used in refinement	48226(4731)	74049(7170)	104393(10066)	67316(6597)	75317(7218)	53958(5297)	99388(9765)	86325(8482)	34170(3158)	42549(4186)	49412(5125)	47523(4684)
Rwork/Rfree	0.1800(0.2251)/ 0.2112(0.2862)	0.1942(0.2547)/ 0.2321(0.3150)	0.1853(0.2495)/ 0.2111(0.2590)	0.1546(0.2050)/ 0.1926(0.2582)	0.1591(0.2196)/ 0.1887(0.2443)	0.2487(0.2608)/ 0.2618(0.3176)	0.1471(0.3401)/ 0.1786(0.3778)	0.1511(0.2055)/ 0.1714(0.2550)	0.1650(0.25589)/ 0.2126(0.3101)	0.1548(0.2520)/ 0.1930(0.2987)	0.1775(0.2974)/ 0.2081(0.3340)	0.1886(0.3308)/ 0.2261(0.3402)
Number of non-hydrogen atoms	4185	4209	4287	4262	4305	4122	4450	4283	4394	4454	4451	4423
macromolecules	3960	3961	3960	3999	3960	3960	3960	3960	3960	4017	4017	3960
ligands	46	11	50	72	66	60	62	44	52	45	43	45
solvent	193	237	291	223	315	128	458	301	406	414	411	441
RMS(bonds) (Å)	0.073	0.059	0.066	0.176	0.1	0.082	0.034	0.091	0.06	0.045	0.042	0.018
RMS(angles) (°)	2.95	1.86	2.18	3.23	1.94	1.97	2.52	1.45	2.61	1.44	1.73	1.57
Ramachandran favored (%)	96.64	96.05	97.04	96.46	96.64	96.64	97.23	96.64	96.84	96.86	96.86	97.04
Ramachandran allowed (%)	3.16	3.16	2.37	3.35	3.16	3.16	2.57	3.16	2.96	2.94	2.94	2.77
Ramachandran outliers (%)	0.2	0.79	0.59	0.2	0.2	0.2	0.2	0.2	0.2	0.2	0.2	0.2
Clashscore	4.62	4.39	3.34	2.92	1.8	1.03	1.16	2.06	2.31	1.9	3.81	1.67
Average B-factor	24.21	22.56	22.2	23.53	22.15	21.52	25.07	22.46	28.84	30.23	23.46	25.36
macromolecules	24.04	22.26	21.78	23.19	21.49	21.53	23.74	21.95	28.32	29.62	22.84	24.85
ligands	29.95	28.39	25.67	30.17	42.03	27.25	31.06	25.63	30.2	28.53	25.03	25.01
solvent	26.68	27.16	27.47	28.37	28.61	19.78	36.23	28.96	33.84	36.25	29.46	29.95

Unusual Crystal Pathology in Amidase U11

The amidase U11 crystallizes in two different conditions and forms a total of three different crystal systems. In the first structure (R32), the solved asymmetric unit of u11 consists of four polypeptide chains (**Fig. S14a**). In the case of the second (P 61 2 2) and third (P 62 2 2) unit cells, we obtain crystals under the same conditions, but due to unresolved indexing ambiguity, some of the crystals are indexed in a large unit cell with a long c-axis (Fig. S1b), and others are indexed in a small unit cell in which the c-axis is approximately half of the first unit cell (**Fig. S14c**). Despite this, amidase U11 packing is the same in both unit cells. In the large unit cell, amidase U11 forms a dimer (**Fig. S14e**), which exactly superimposes with the dimer of the tetramer from the first unit cell (i.e., R32). In the case of the small unit cell, the u11 asymmetric unit consists of a monomer (**Fig. S14f**). This unusual phenomenon is not completely resolved despite extensive analysis for possible pseudosymmetry, twinning, NCS, or any other crystal pathology by using phenix.xtriage (Adams *et al*., 2010) (**Fig. S15bcd.)**. Data reduction by XDS (Kabsch, 2010) and space group determination by POINTLESS (Evans, 2011) do not indicate any problem with indexing and merging statistics. Molecular replacement by PHASER (McCoy et al., 2007) finds a single solution with a very high LLG (28509.388) and TFZ score (140.9), which indicates most likely the correct single solution; yet final R-free values after several rounds of refinement remain too high (Fig. S12c) for the given resolutions. Nevertheless, no problem is observed with the modelled electron density map (Fig. S1a). Nevertheless, we locate several large positive peaks in the middle of the lattice, which cannot be accounted for by any buffer components (**Fig. S15a**). We model these difference map peaks as a degradation product of amidase, which becomes a part of the crystal lattice (**Fig. S16b**), as we observe lower bands in SDS-PAGE (**Fig. S1b**). Modelling of the degradation product results in a significantly better R-free factor; however, the final difference map still indicates problems associated with the degradation product, which is very hard to identify with the correct composition.

We then tried to reprocess the data to C222(1) space group which is subgroup of P622. Unit cell dimensions in both space groups have same a-and c-axis whereas b-axis of centric orthorhombic space group is equal to √3 multiplied to a-axis of hexagonal space group (**Fig. S17**) (Yeates & McPherson,2019). Reprocessing to C222(1) space group give reasonable statistics (**Table S6**). MR solution from phaser find 8 molecules of amidase U11 and moreover xtriage analysis indicates twin law 1/2*h+1/2*k,3/2*h-1/2*k,-l with almost perfect twin fraction (0.53) which is an example of pseudo-merohedral twining that cannot be detected by intensity statistics analysis. By using the twin law in the refinement with phenix.refine, we were able to obtain better R-free values compared to the R-free values obtained from the equivalent dataset processed in P622. Close analysis of the crystal lattice in C222(1) indicates tighter packing and reduced difference map peaks in the solvent channel (**Fig. S18b**). However, even after several rounds of refinement with water molecules, significant

fractions of amidase U11 molecules contain several difference map peaks associated with the main chain (**Fig. S19b**), and there is even one molecule with almost no clear density for the main chain (**Fig. S19a**). Further analysis for omit map for the fragment (VT00232, PDB: 30QO), indicate lower map quality compared to P622 equivalent (**Fig. S19ef**). Although C2221 explains crystal symmetry and packing better than P622, as shown by lower R-free values, the resulting difference map peaks in solvent channels, and the final electron density maps are worse, assesed from visual inspection (**Fig. S19d**). To decide whether local electron density maps in P622 can still be used to validate ligand binding, we calculated an unbiased omit map by removing the fragment and associated binding environment atoms (e.g., water molecules and surrounding residues) and refined against P622 data without occupancy refinement. As a result, we show clear unbiased omit maps for all omitted atoms, which can still be used for fragment binding analysis even though global R-free values are low (**Fig. S20ab**). This unique phenomenon of global disagreement in crystal packing, whereas local agreement in electron density maps, does not hinder our analysis of fragment binding, as we have several unbiased pieces of evidence from omit maps and real-space metrics (e.g., RSR and RSCC) in PDB validation reports. Therefore, we decided to continue with the first model without any degradation product, as we do not encounter any problems in analysing local electron densities to identify fragment binding. Future fragment-screening studies may still be useful, even with global crystal pathology (in our case pseudo-merohedral twinning), if unbiased fragment omit maps can be obtained from high-symmetry equivalents of perfectly twinned crystals.We also shared the diffraction patterns of the amidase U11 in zenodo(https://doi.org/10.5281/zenodo.22855430) for the data set that is used in here.

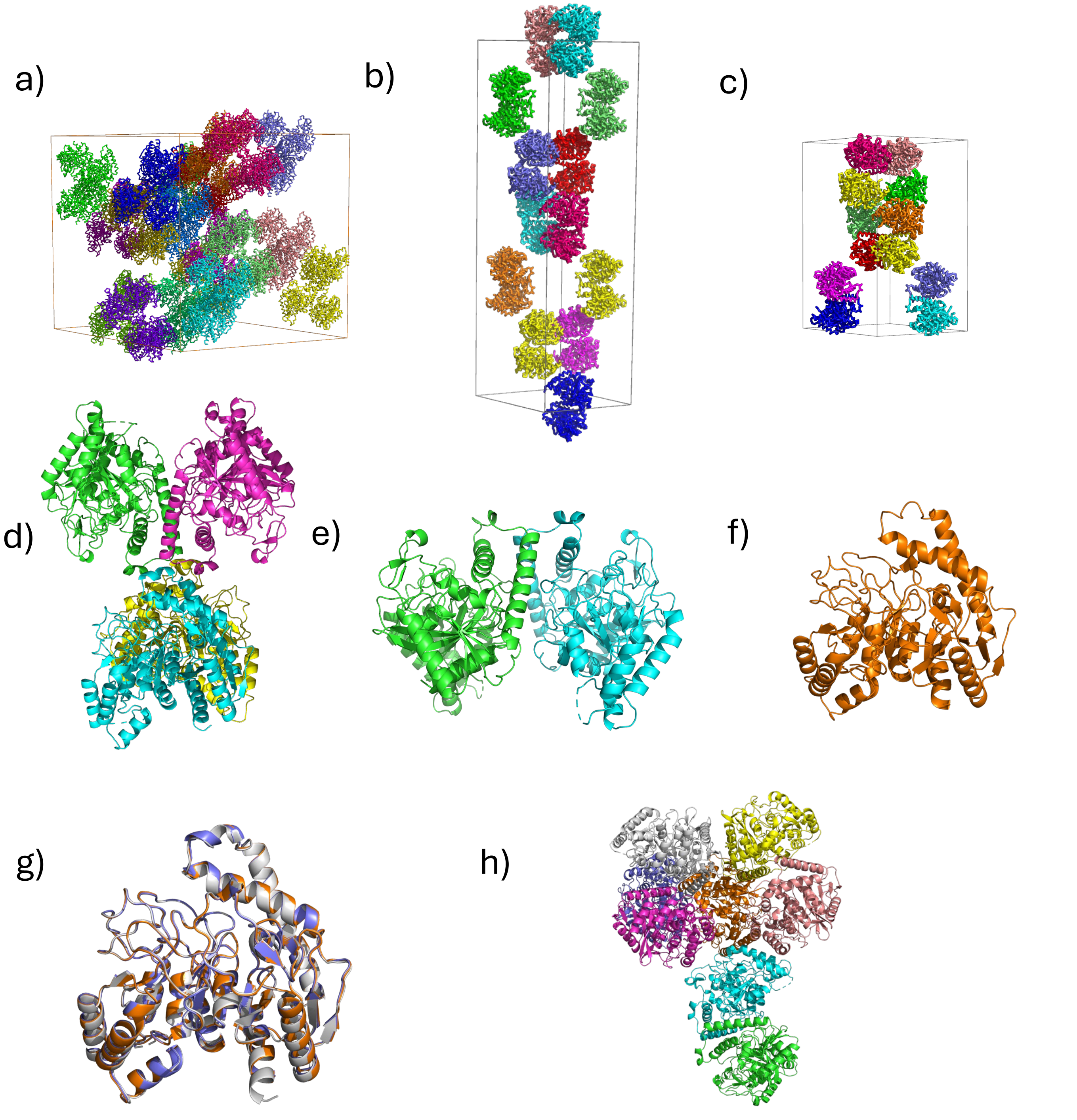

Figure S14 different unit cell and space groups for amidase U11. a) R32 b) P6122 and c) P6222 and corresponding asymmetric units for d) R32, e) P6122 and f) P6222. The dimer interface is the same in all crystal systems. g) Structural superimposition of single u11 polypeptide chain indicates no significant conformational change (C_{α} rmsd < 0.3 A). h) Cartoon representation of eight molecules of amidase U11 in asymmetric unit of pseudo-merohedral twinning C2221 space group.

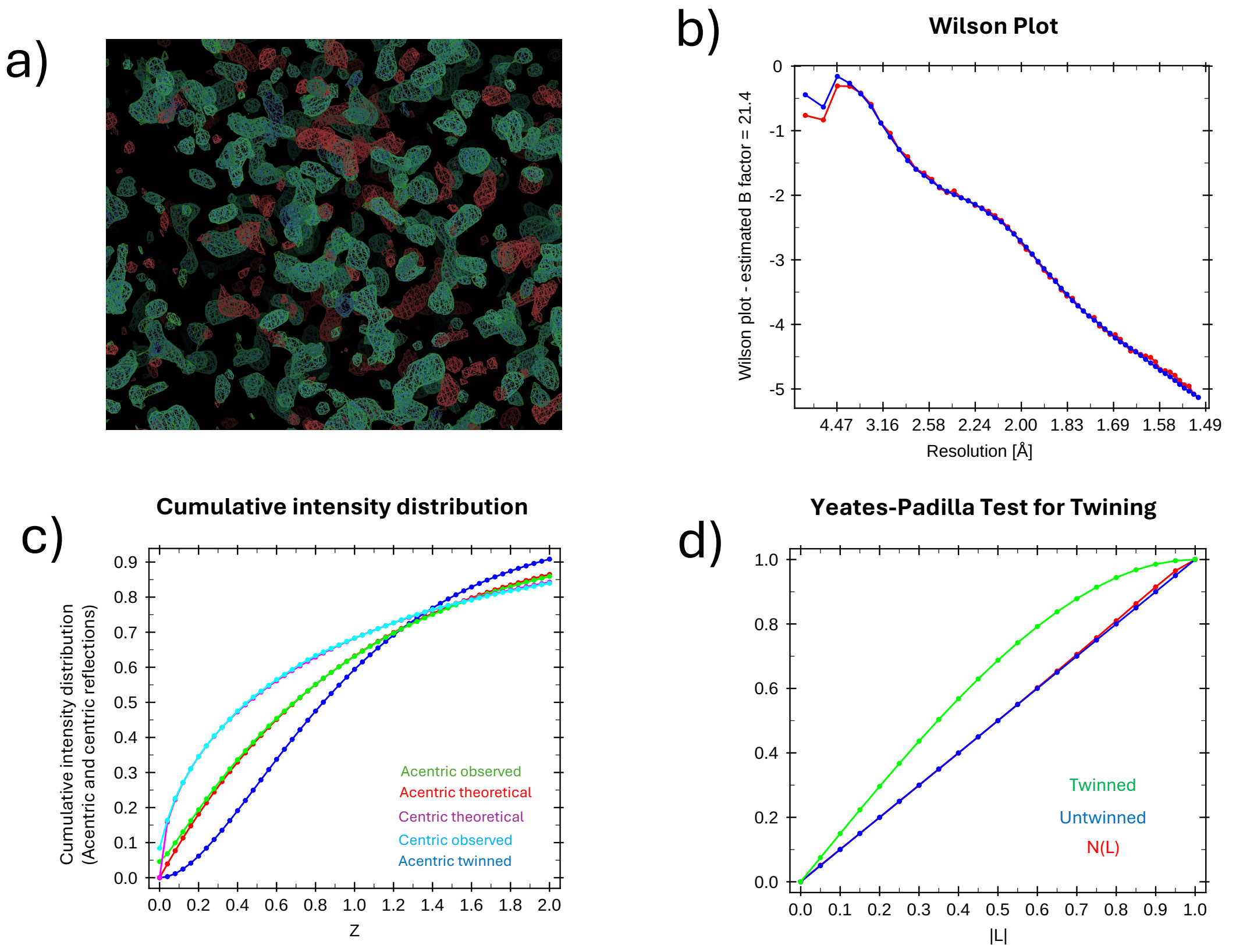

Figure S15 Analysis of cumulative intensity distribution statistics for twinning and pseudo translational symmetry do not indicate any apparent problem. a) unmodelled density in the intermolecular solvent regions. b) Wilson plot c) Cumulative intensity distribution for acentric and centric reflection and d) Yeates-Padilla test for twinning and pseudo translational symmetry.

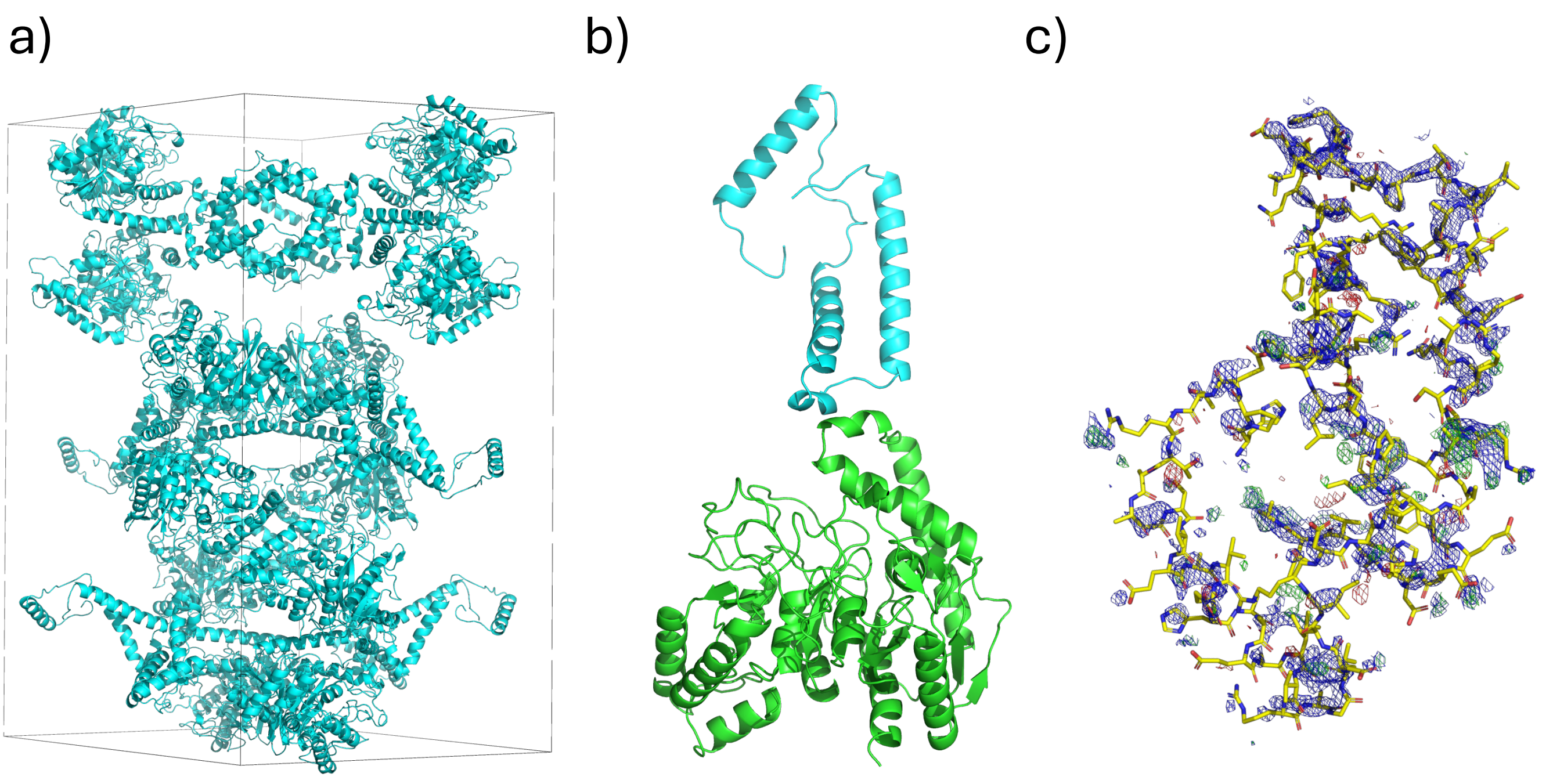

Figure S16 Modelling of unexplained blobs as a degradation product of u11. a) shows unit cell packing in P6222 space group with degradation product located. b) asymmetric unit of u11(green) with degradation product(cyan) and c) corresponding electron density map for modelled degradation product. 2mFo-DFc map at 1.0σ and mFo-DFc map at $\pm$ 3.0σ contour levels after refinement.

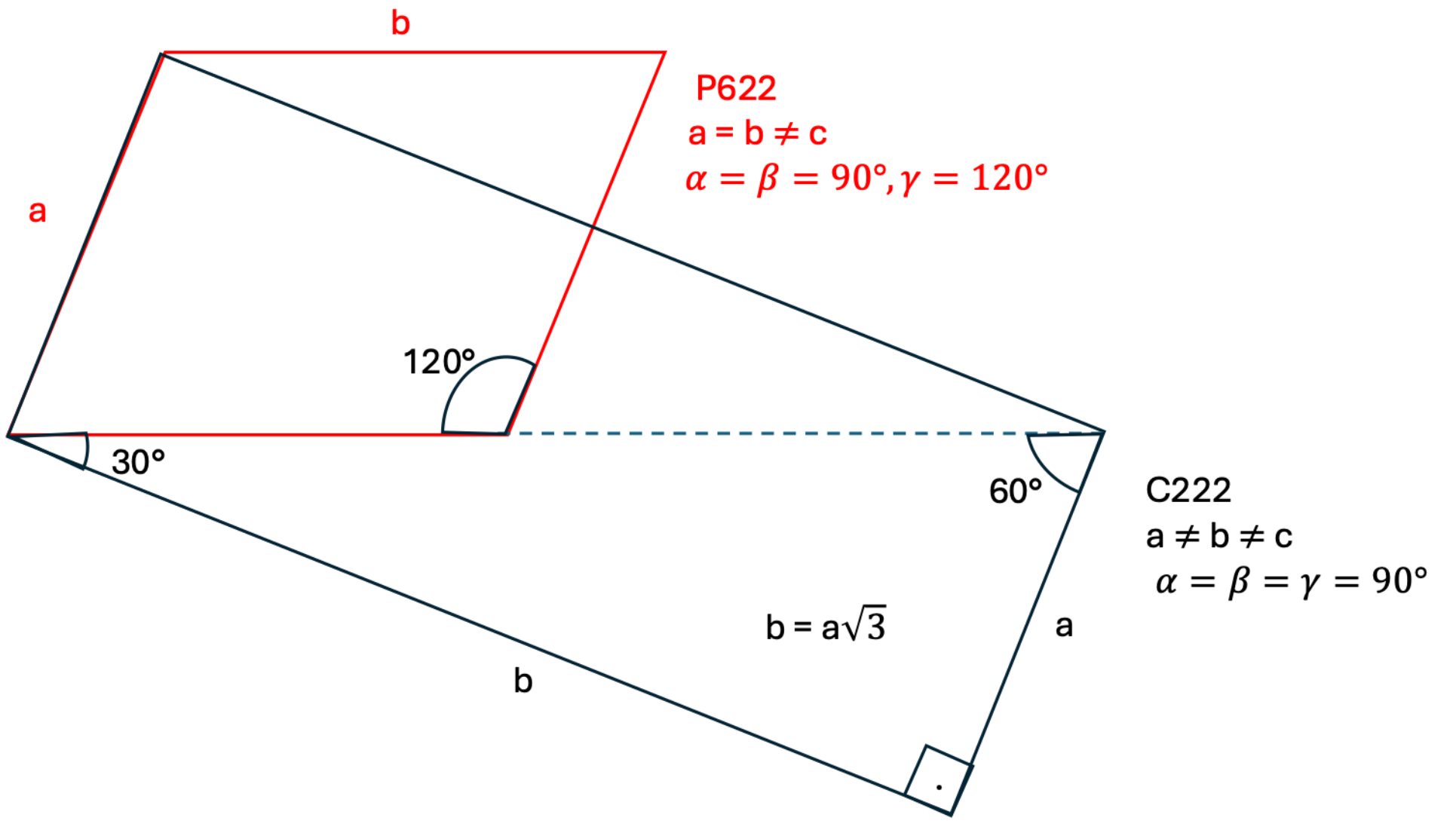

Figure S17 unit cell axis transformation in P622 and C221 space group

Table S6 x-ray diffraction data processing and refinement statistics for pseudo-merohedral twinned data set of amidase U11 (VT00232). Parenthesis are for high resolution shell.

PDB code	34FS
Resolution	49.63-1.697
Space Group	$C222_1$
a, b, c (Å)	111.43,193.25,436.77
α, β, γ (°)	90,90,90
Number of total reflections	7021017(970460)
Number of unique reflections	509143(78140)
Completeness(%)	99.1(94.5)
Redundancy	13.7(12.4)
Rmeas (%)	7.7(88.3)
$\langle I/\sigma(I) \rangle$	21.92(2.81)
$CC_{1/2}$	100(79.2)
Twin Law	1/2*h+1/2*k,3/2*h-1/2*k,-l
Twin fraction	0.53
Rw/Rf	22.47/24.97

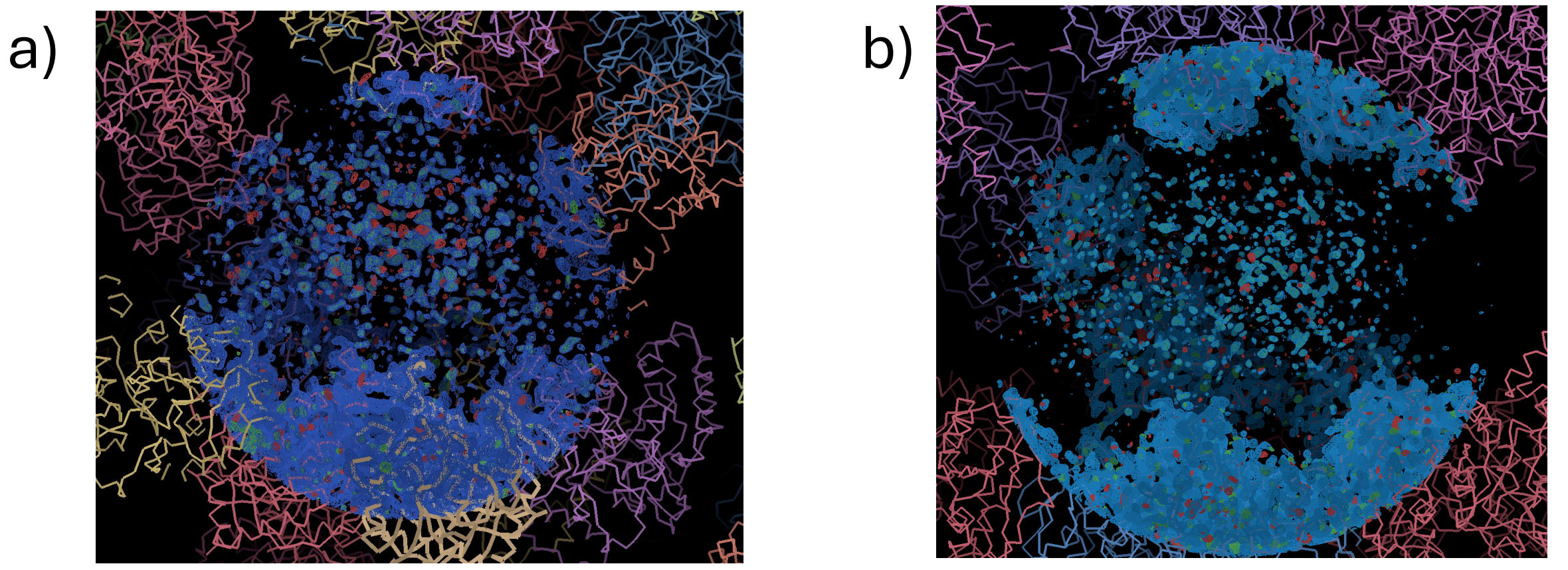

Figure S18 Comparison of crystal packing and solvent channel difference map peaks in P622 and C2221 space group. a) P622 and b) C2221

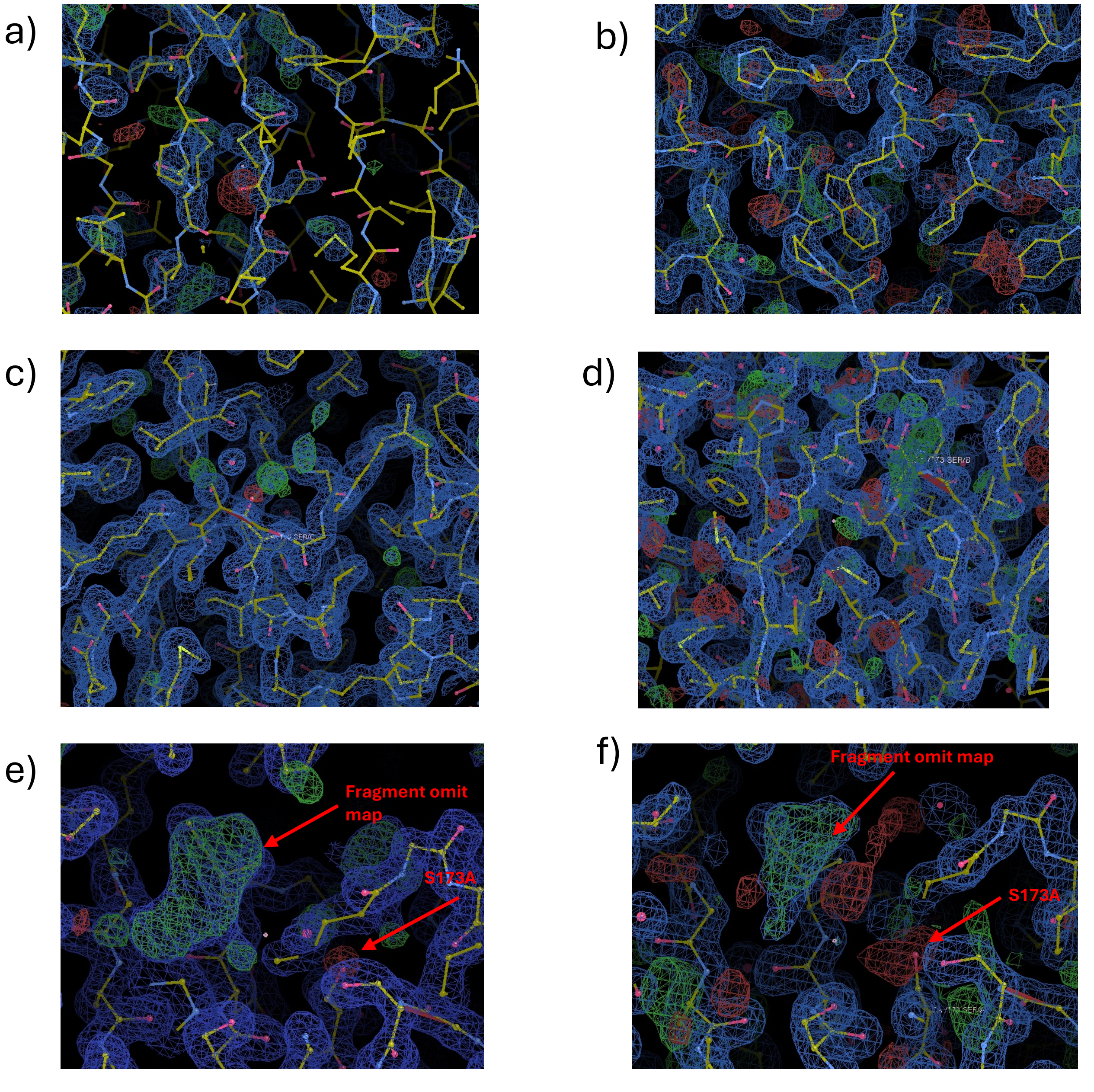

Figure S19 Comparison of electron density maps for VT00232 (PDB: 30QO) for P622 and C2221 space groups. a) vague electron density map for one of the eight molecules of amidase U11 b) difference map peaks associated with main chain atoms of amidase U11 molecules. c) overall electron density maps in P622 and d) C2221 with negative difference map peak on S173A mutation. e) omit map for VT00232 in P622 and f) C2221. All 2mFo-DFc maps are depicted at 1.5σ and mFo-DFc maps at $\pm$ 3.0σ contour levels after refinement.

a)

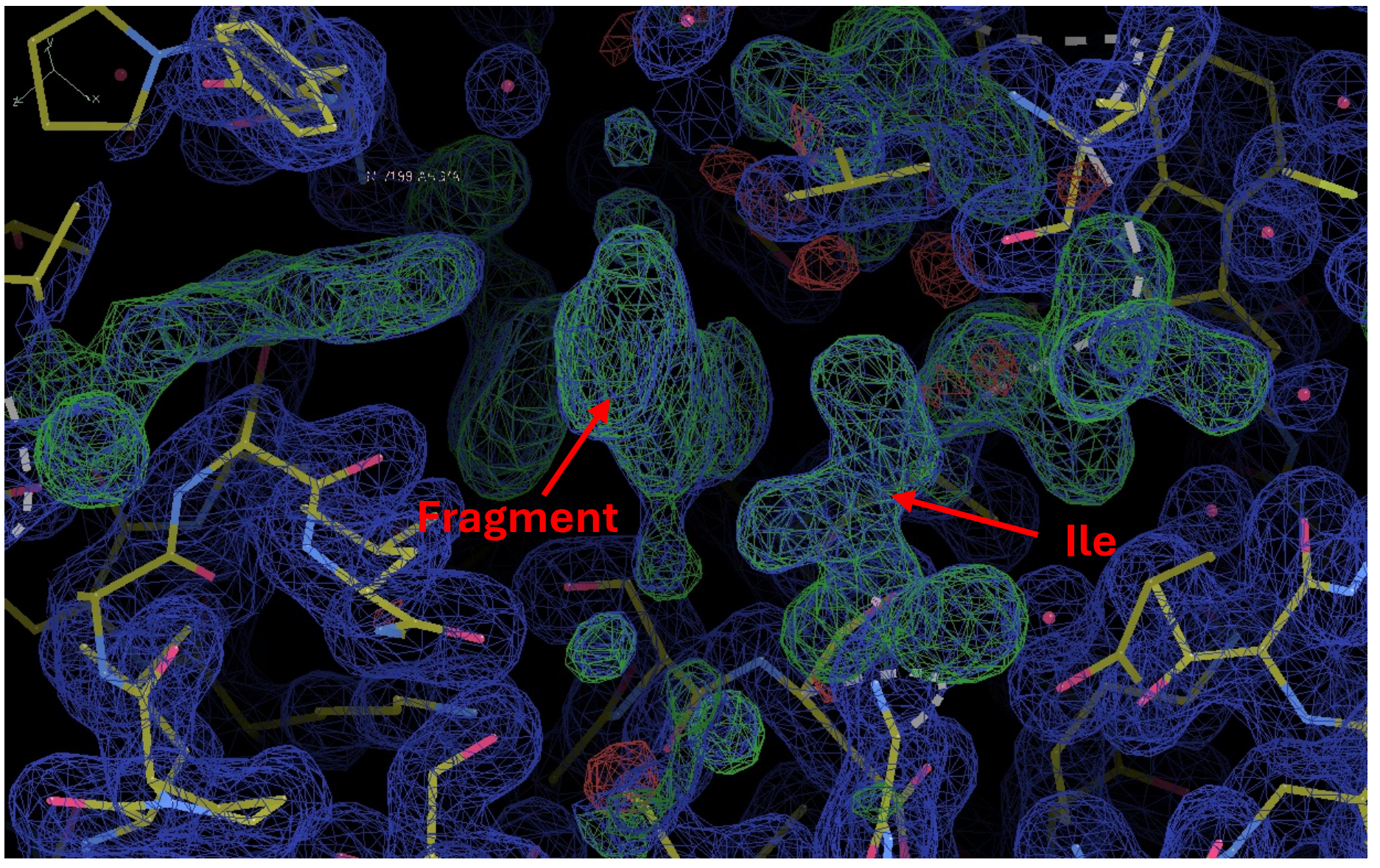

b)

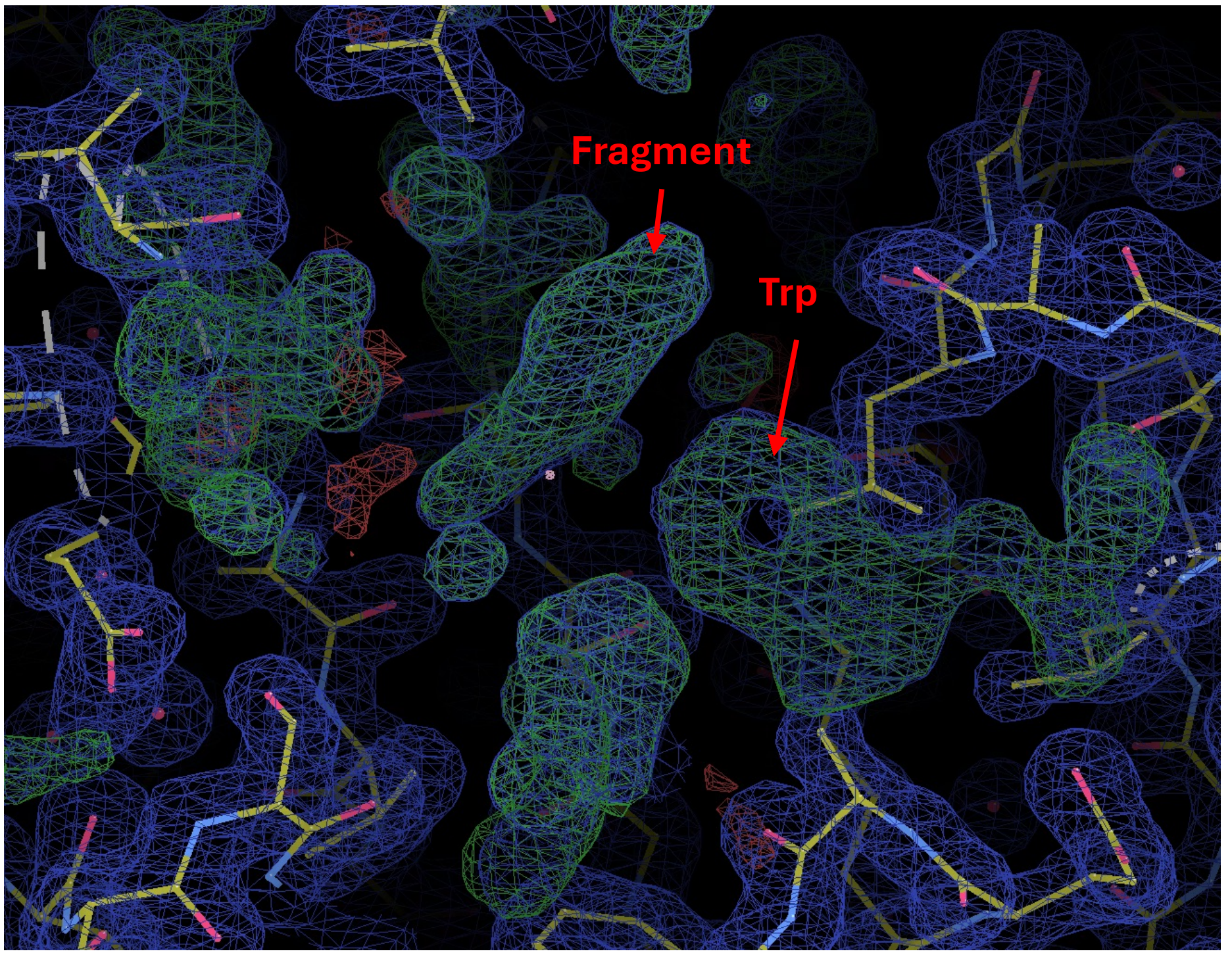

Figure S20 Unbiased omit maps for ligand binding environment in P622. a) clear omit map for fragment and binding environment. Ile residue omit map is highlighted for reference. b) different view of the same binding environment. Trp residue omit map is highlighted. All 2mFo-DFc maps are depicted at 1.5σ and mFo-DFc maps at $\pm$ 3.0σ contour levels after refinement.

References:

Yeates, T. O., & McPherson, A. (2019). The structure of bovine β-lactoglobulin in crystals grown at pH 3.8 exhibiting novel threefold twinning. *Structural Biology and Crystallization Communications*, *75*(10), 640-645.

Supplementary information for PU fragment synthesis

1- Polyurethane Fragment Synthesis

4,4-MDA, 2,4-TDA and 2,4-TDI are bought from Sigma Aldrich and directly used for soaking experiment by solubilizing in DMSO. DUE-MDA, DUE-TDA, MUE-TDA and MUE-MDA are synthesized as described previously (Bendsten et al., 2026)

2- Transition State Analogues Synthesis

MDA-TS analog

2-(2-(2-methoxyethoxy)ethoxy)ethyl (4-nitrophenyl) (4-benzylphenyl)phosphoramidate

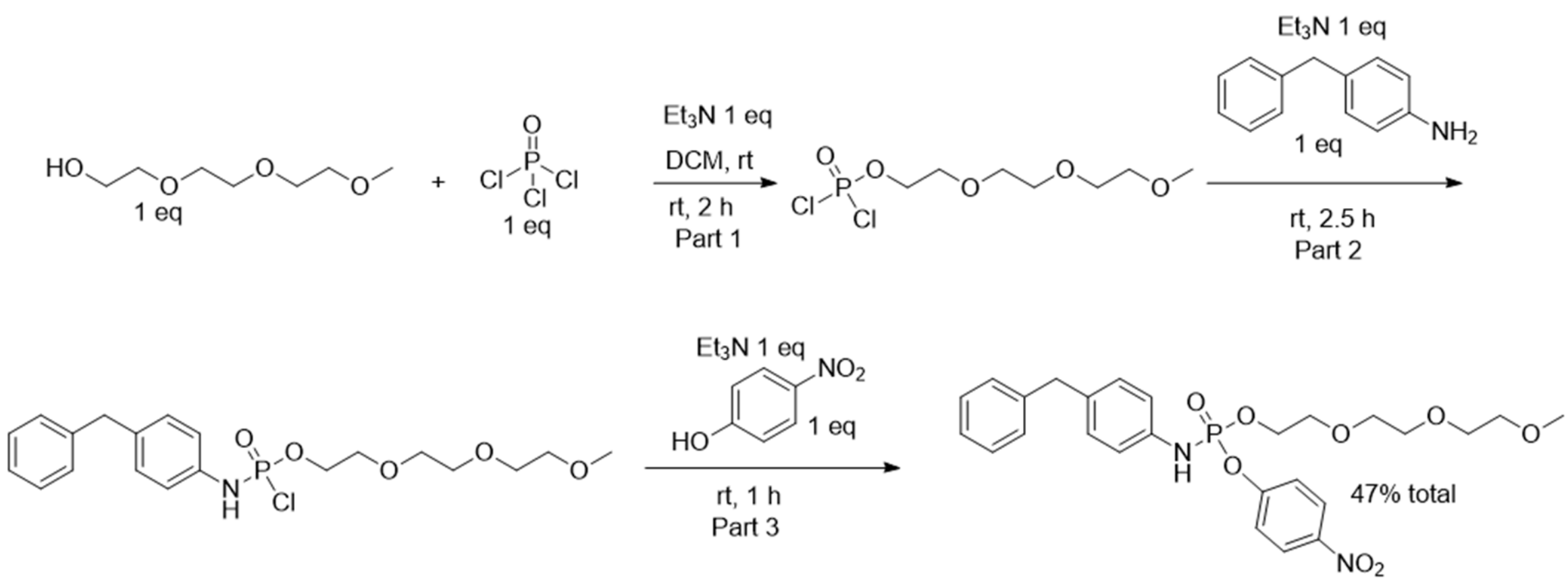

Figure S21. Reaction scheme of MDA-TS analog synthesis

Part 1

$POCl_3$ (2.0 mL, 1 equiv.) was added to CH_2Cl_2 (50 mL) in a 100 mL flask under a nitrogen atmosphere. Triethylene glycol methyl ether (3,44 mL, 1 equiv.) was added to the reaction by syringe followed by slow addition of Et_3N (3,0 mL, 1 eq) by syringe. Heat and bubbles were observed. The reaction is followed by P-NMR with decoupling. 0.5 mL was taken directly from the reaction into a NMR tube then analyzed. The $POCl_3$ peak disappeared and a single new peak was formed within 30 min. After 2 hours, continued to part 2.

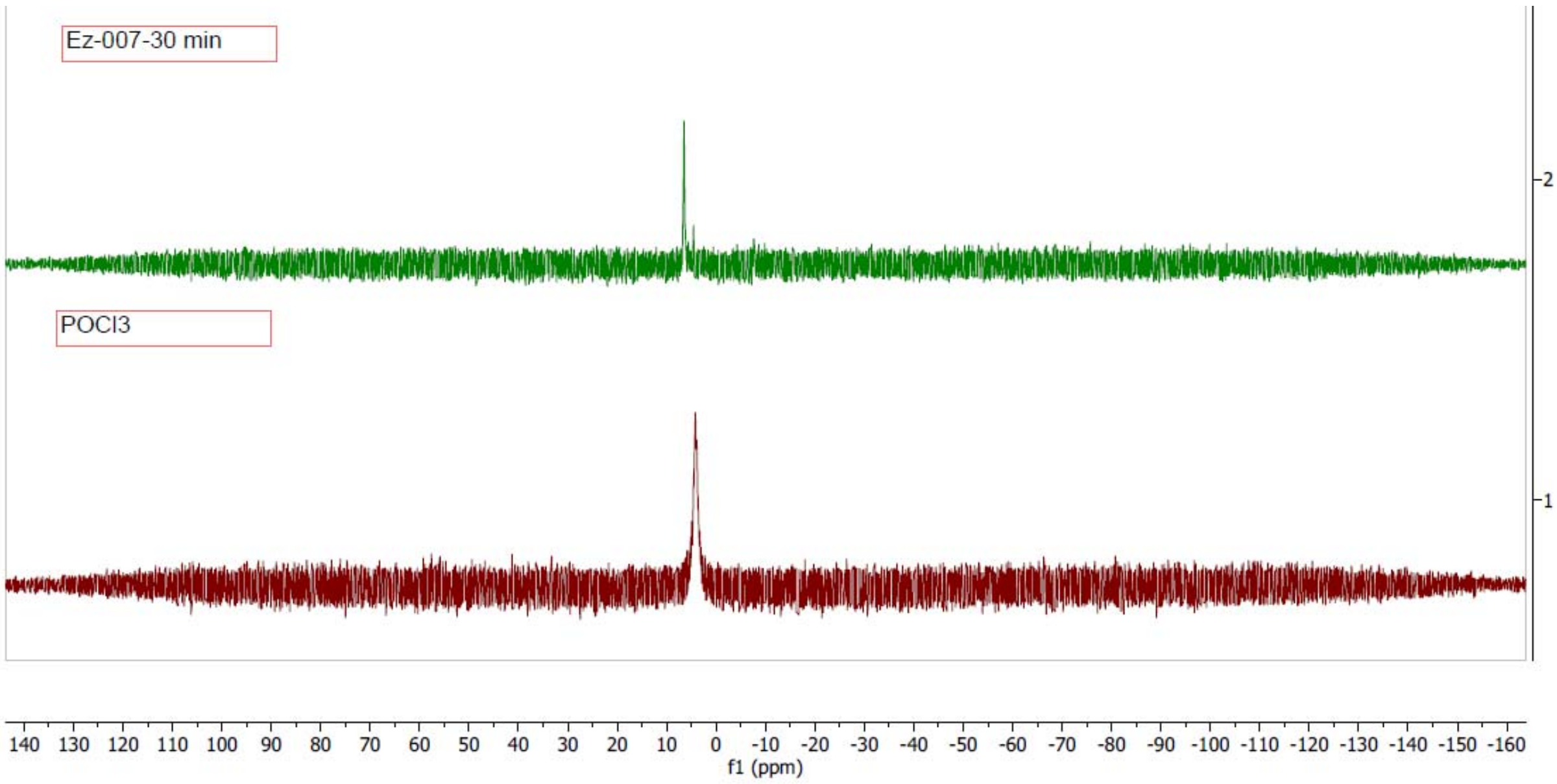

Figure S22. P-NMR spectrum for reaction monitoring of part 1.

Part 2

4-benzylaniline (3,93 g, 1 equiv.) was dissolved in CH_2Cl_2 (10 ml) and added to the reaction. Triethylamine (3 mL, 1 equiv) was then added. The reaction mixture was allowed to stir at rt. Reaction monitoring performed by P-NMR.After 2 hours, full conversion was seen to mainly one new product. Proceed to part 3.

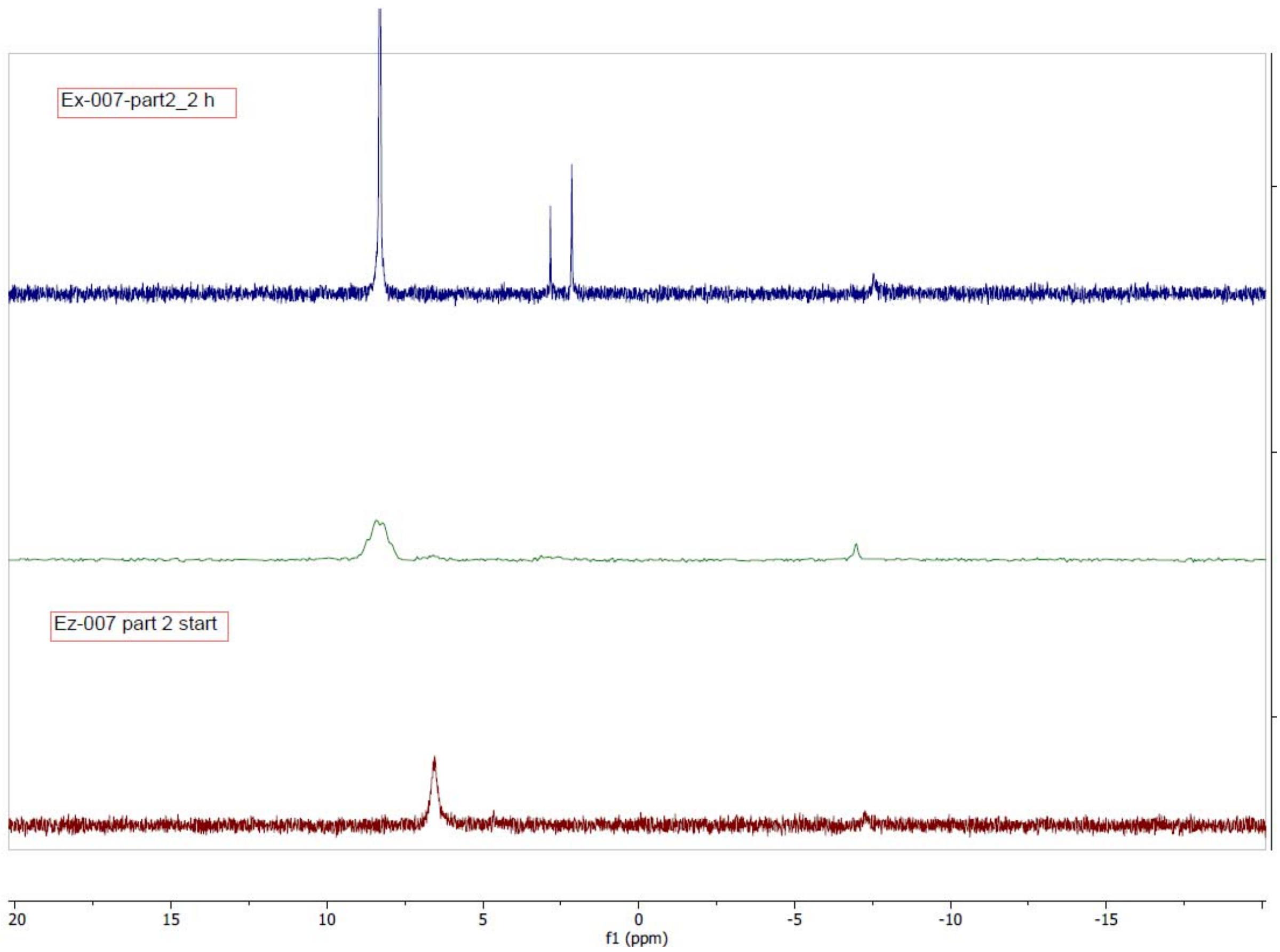

Figure S23. P-NMR spectrum for reaction monitoring of part 2.

Part 3

Triethylamine (3.0 mL, 1 equiv) was added followed by 4-nitrophenol (3,0 g, 1 equiv). The reaction mixture goes yellow upon addition. A white solid is seen in the reaction (likely, Et_3N-HCl salt). After 1 hour, P-NMR show new peaks, the peak at –1.81 ppm is the major product. The reaction was filtered to remove the salt and concentrated onto celite.

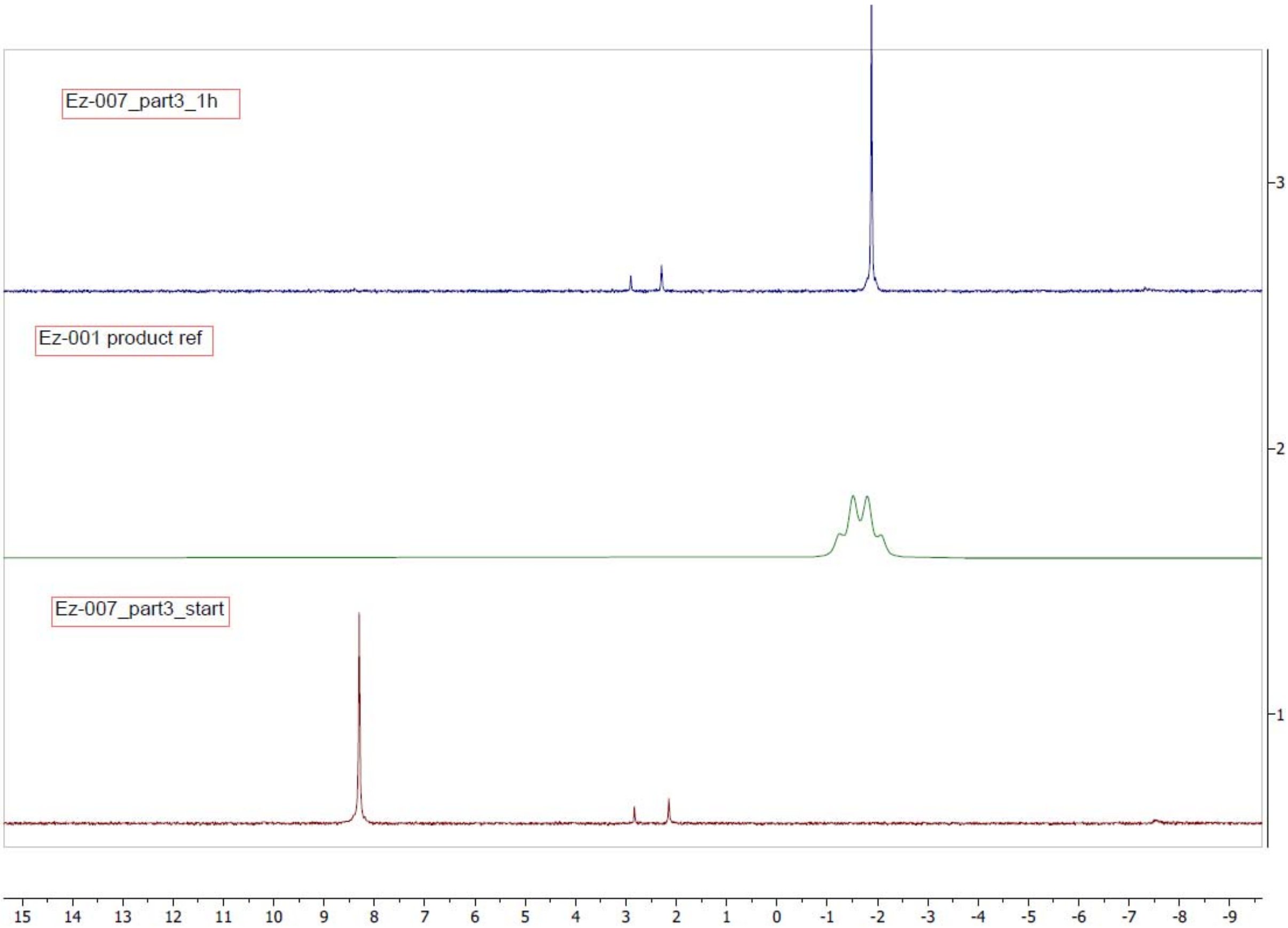

Figure S24. P-NMR spectrum for reaction monitoring of part 3.

Purification:

Pure C-850 FlashPrep (80 g column, heptane:EtOAc 40:60 to 0:100. The fractions containing product were concentrated to afford an oil. The oil was dried overnight under vacuum to yield the product (5,22 g, 47%) as a thick yellow oil. The product does not show any degradation by P-NMR over 7 days in MeOH or water/DMSO 1:4 solution at ambient temperature.

^{1}H NMR (400 MHz, DMSO) δ 8.60 (d, J = *10.3* Hz, 1H), 8.31 – 8.23 (m, 2H), 7.48 – 7.40 (m, 2H), 7.29 – 7.22 (m, 2H), 7.22 – 7.13 (m, 3H), 7.13 – 7.06 (m, 2H), 7.06 – 6.96 (m, 2H), 4.32 – 4.14 (m, 2H), 3.84 (s, 2H), 3.63 (td, J = *4.6, 1.7* Hz, 2H), 3.52 – 3.42 (m, 6H), 3.42 – 3.32 (m, 2H), 3.21 (s, 3H).

^{13}C NMR (101 MHz, DMSO) δ 155.9 (d, *J* = *5.9* Hz), 144.5, 142.0, 138.1, 134.9, 129.9, 129.0, 128.8, 126.3, 126.3, 121.4 (d, *J* = *5.2* Hz), 118.4 (d, *J* = *7.8* Hz), 71.7, 70.2, 70,2, 70.1, 69.6 (d, *J* = *7.2* Hz), 66.7 (d, *J* = *5.4* Hz), 58.5, 40.8.

^{31}P NMR (162 MHz, DMSO) δ -1.83 (q, *J* = *8.6* Hz)

HRMS (ESI+): m/z calcd. for $C_{26}H_{32}N_2O_8P$ $[M+H]^+$: 531.1891, found 531.1905.

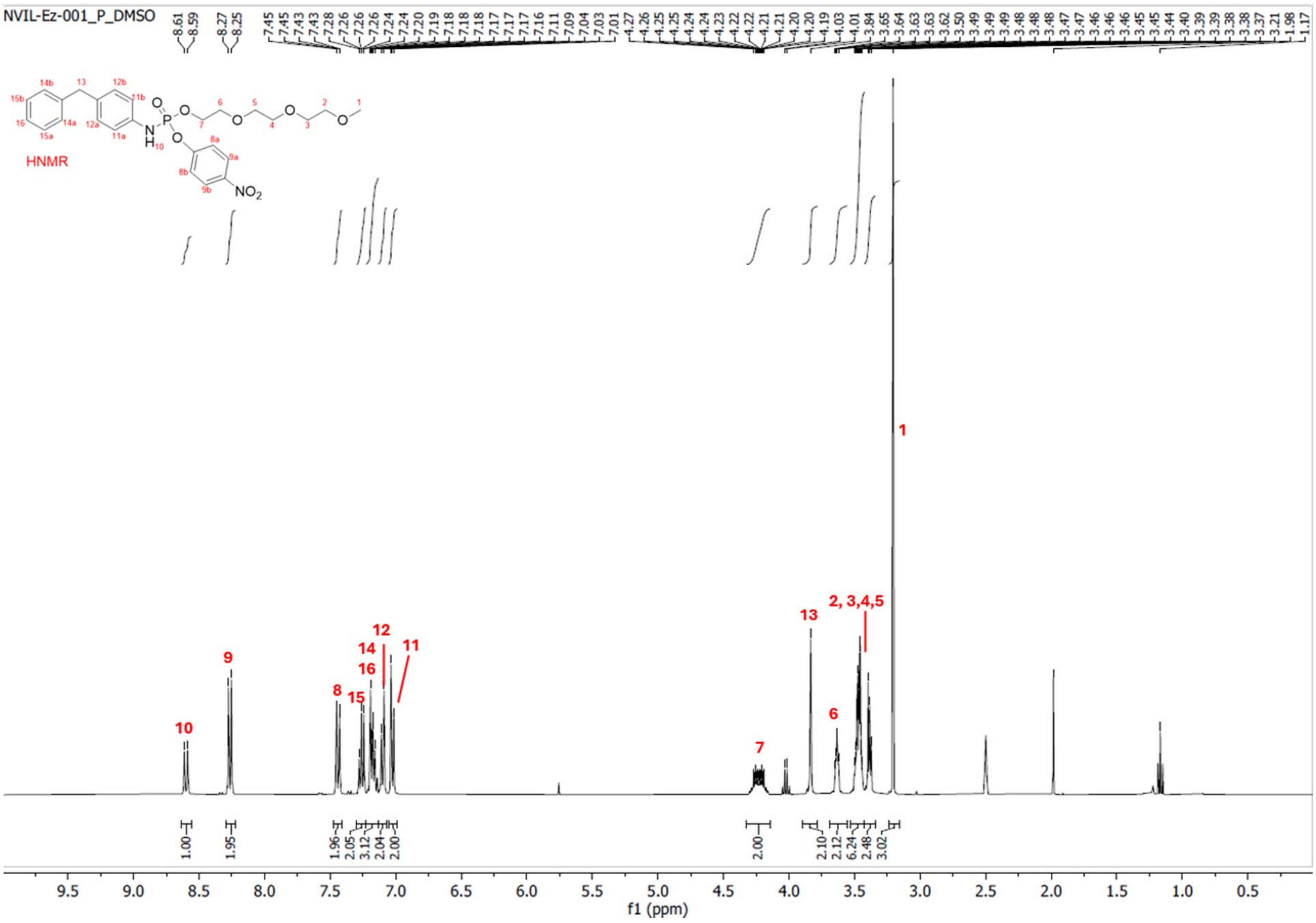

Figure S25. H-NMR spectrum for MDA-TS analog.

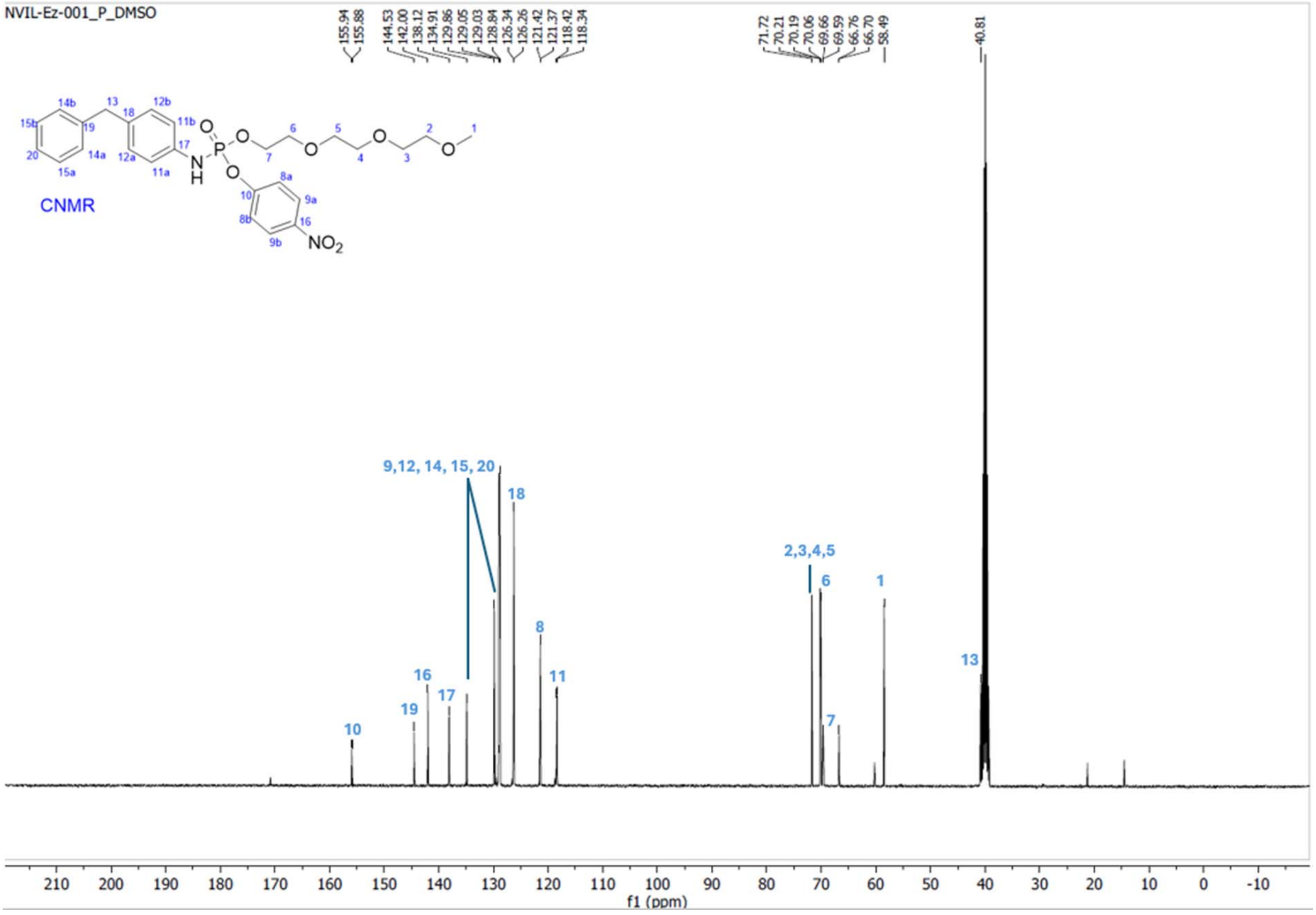

Figure S26. C-NMR spectrum for MDA-TS analog.

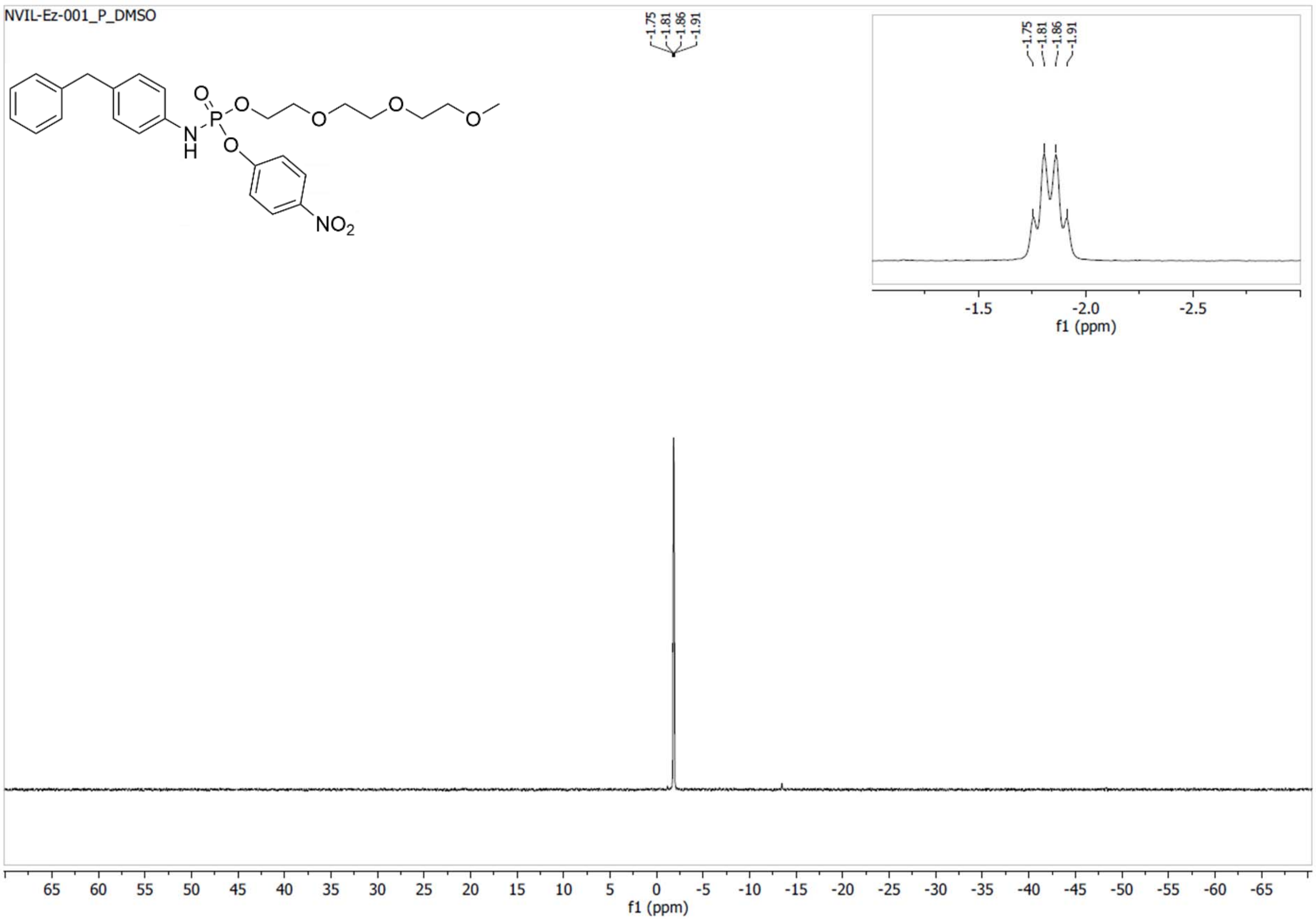

Figure S27. P-NMR spectrum for MDA-TS analog.

2-(2-(2-methoxyethoxy)ethoxy)ethyl (4-nitrophenyl) (5-chloro-2-methylphenyl)phosphoramidate TDA-TS analog

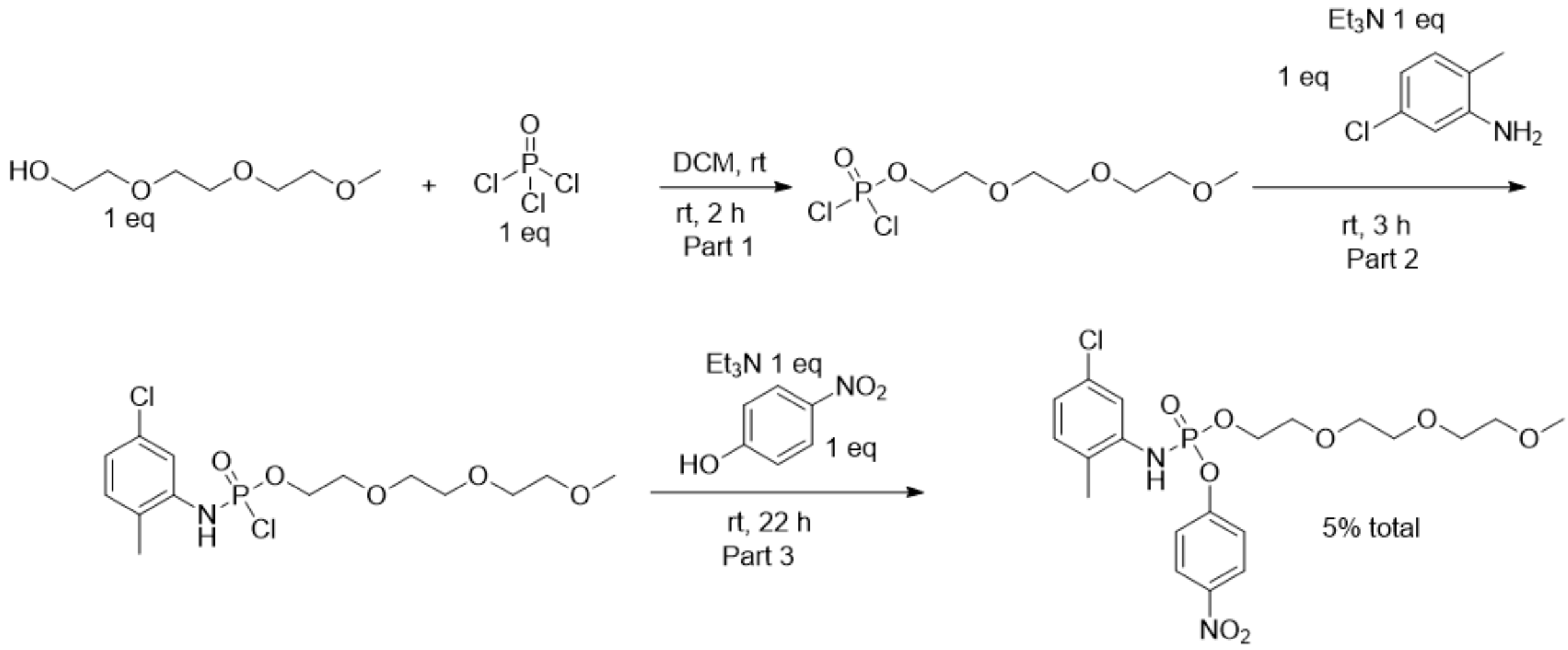

Figure S28. Reaction scheme of TDA-TS analog synthesis

Part 1:

$POCl_3$ (2.0 mL, 1 equiv.) was added to CH_2Cl_2 (50 mL) in a 100 mL flask under a nitrogen atmosphere. Triethylene glycol methyl ether (3,44 mL, 1 equiv.) was added to the reaction by syringe followed by slow addition of Et_3N (3,0 mL, 1 eq) by syringe. Heat and bubbles were observed. The reaction is followed by P-NMR with decoupling. 0.5 mL was taken directly from the reaction into a NMR tube then analyzed. The $POCl_3$ peak disappeared and a single new peak was formed within 30 min. After 2 hours, continued to part 2.

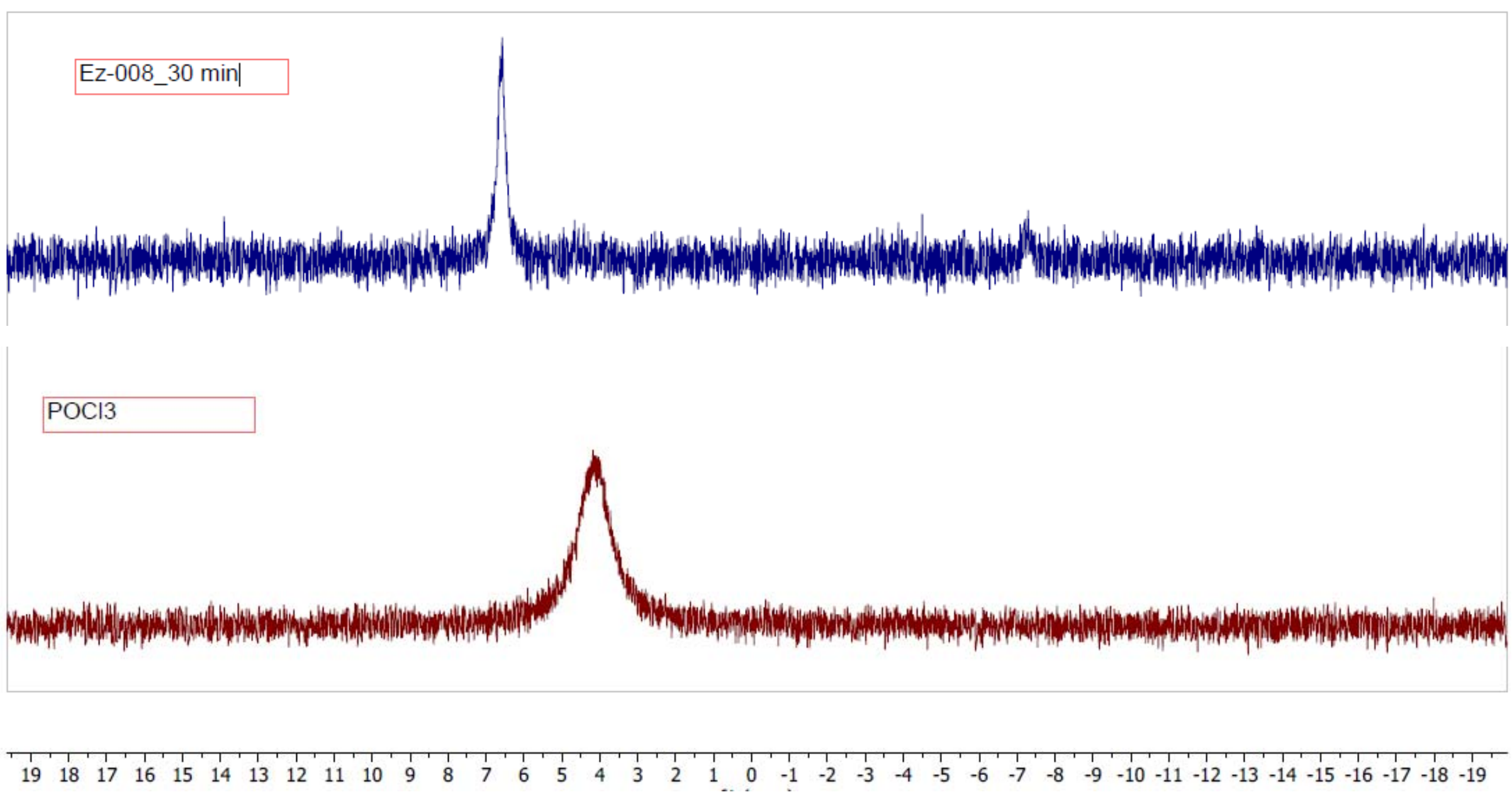

Figure S29. P-NMR spectrum for reaction monitoring of part 1.

Part 2

4-chloro-2-methylaniline (3,04 g, 1 equiv.) was dissolved in CH_2Cl_2 (10 ml) and added to the reaction. Triethylamine (3 mL, 1 equiv.) was then added. The reaction mixture was allowed to stir at rt. Reaction monitoring performed by P-NMR. After 3 hours, almost full conversion of starting peak, but several new peaks were observed. Continued to part 3.

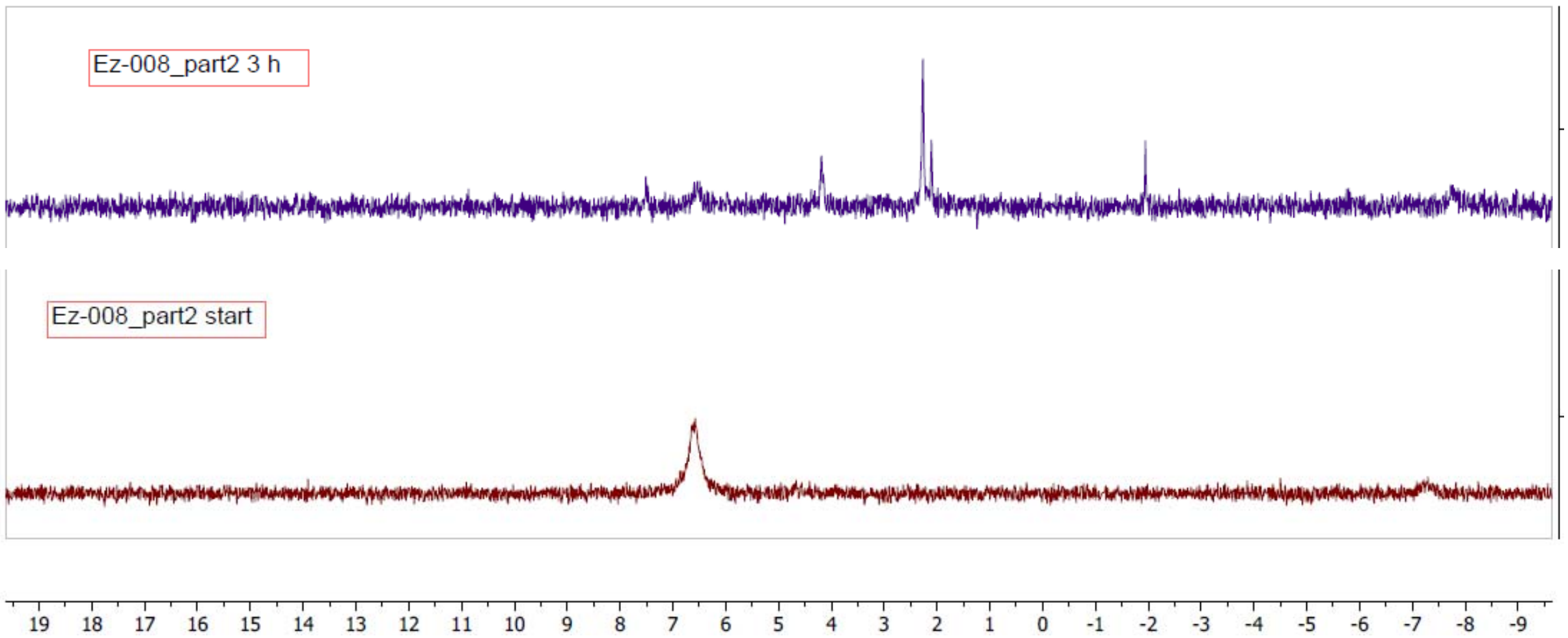

Figure S30. P-NMR spectrum for reaction monitoring of part 2.

Part 3

Triethylamine (3.0 mL, 1 equiv.) was added followed by 4-nitrophenol (3,0 g, 1 equiv.). The reaction mixture goes yellow upon addition. A white solid is seen in the reaction (likely, Et_3N-HCl salt). After 22 h, P-NMR does not show full conversion of main peak. Additional 0.33 eq Et_3N and 4-nitrophenol was added. After 40 h, still not full conversion.

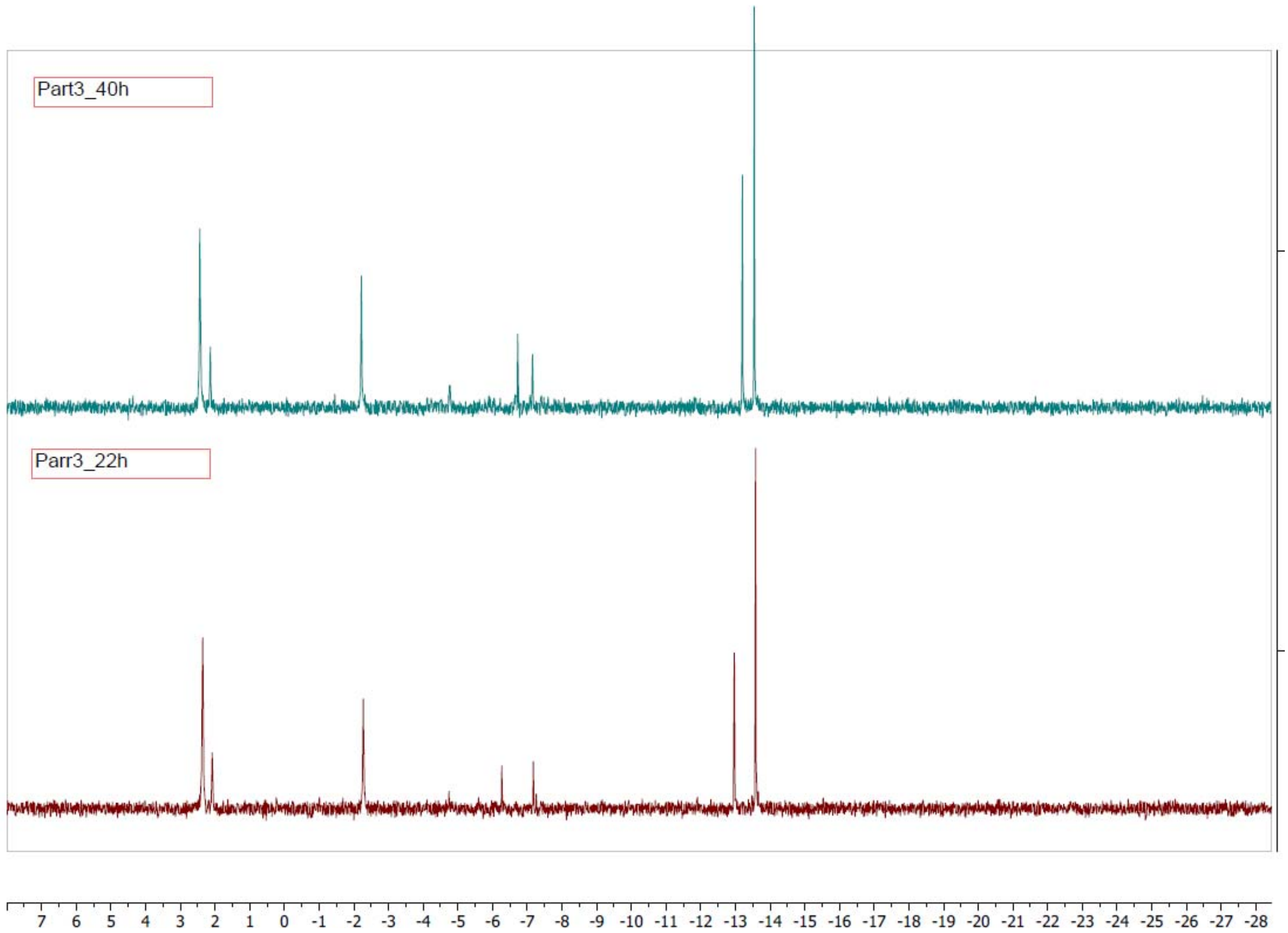

Figure S31. P-NMR spectrum for reaction monitoring of part 3.

Purification:

Pure C-850 FlashPrep (80 g column, heptane:EtOAc 50:50 to 0:100. The fractions containing product were concentrated to afford an oil. The oil was dried overnight under vacuum to yield the product (0.55 g, 5%).

^{1}H NMR (400 MHz, DMSO) δ 8.34 – 8.25 (m, 2H), 7.90 (d, *J* = *8.7* Hz, 1H), 7.45 (dt, *J* = *8.9, 2.0 Hz*, 2H), 7.27 (d, *J* = *2.2 Hz*, 1H), 7.16 (d, J = *8.2 Hz*, 1H), 7.02 (dd, *J* = *8.1, 2.3 Hz*, 1H), 4.28 (pq, *J* = *8.3, 5.0 Hz*, 2H), 3.72 – 3.64 (m, 2H), 3.59 – 3.43 (m, 6H), 3.39 (dd, *J* = *5.9, 3.6 Hz*, 2H), 3.21 (s, 3H), 2.14 (s, 3H).

^{31}P NMR (DMSO) δ -2.4 (q, *J* = *8.6* Hz)

HRMS (ESI+): m/z calcd. for $C_{20}H_{27}ClNO_8P$ $[M+H]^+$: 489.1188, found: 489.1201.

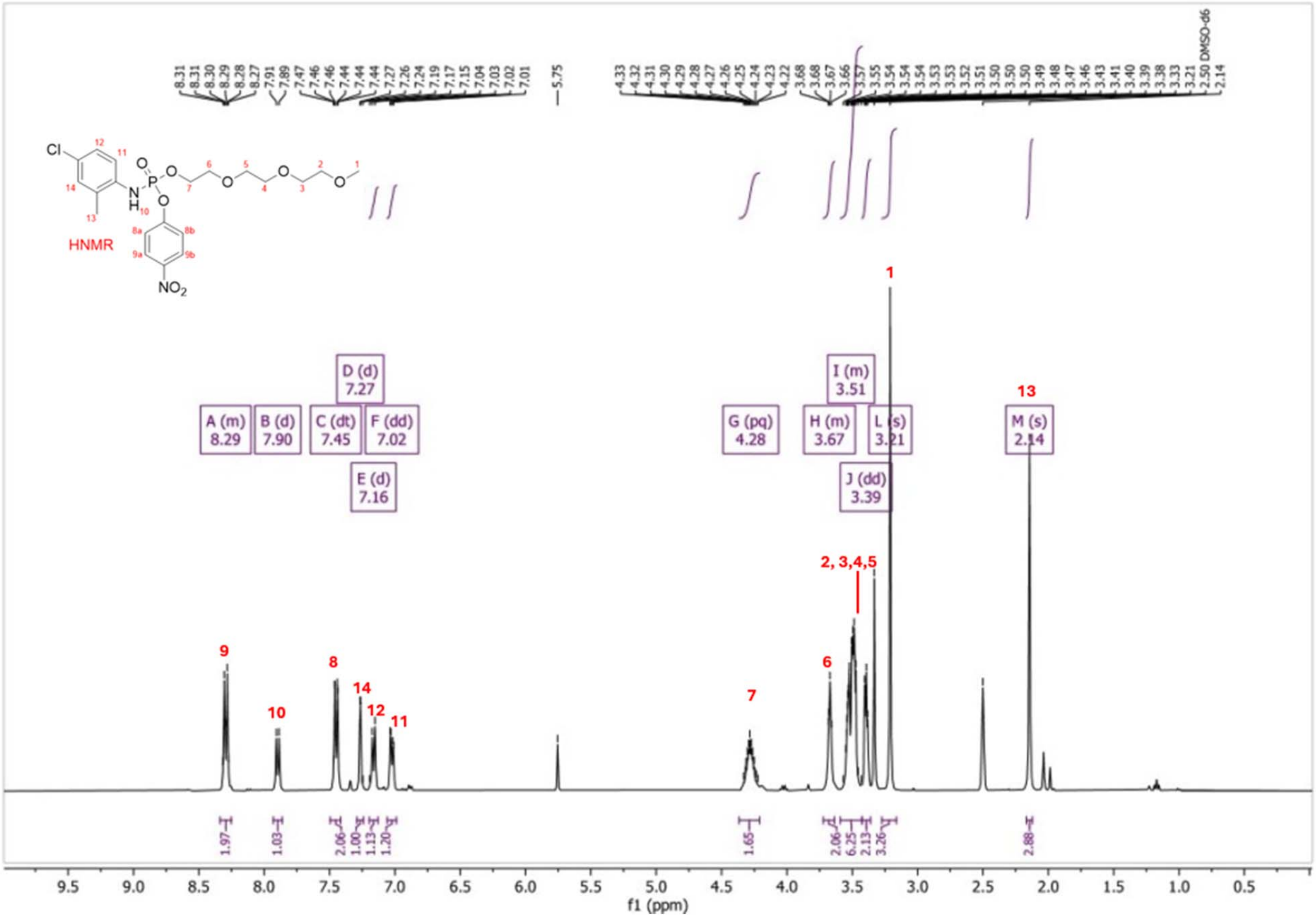

Figure S32. H-NMR spectrum for TDA-TS analog.

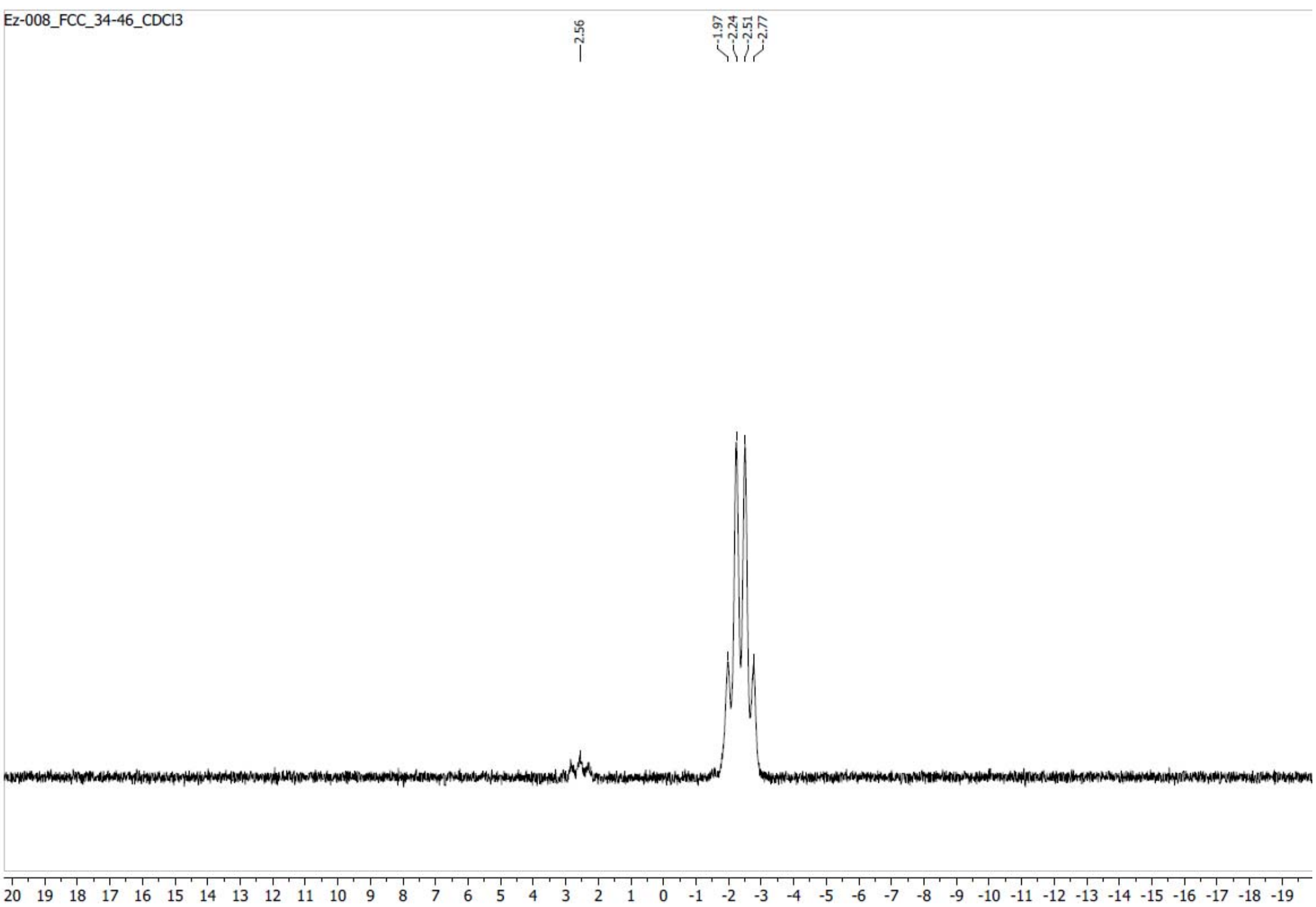

Figure S33. P-NMR spectrum for TDA-TS analog.

3- 2,6-TDA Fragment Synthesis

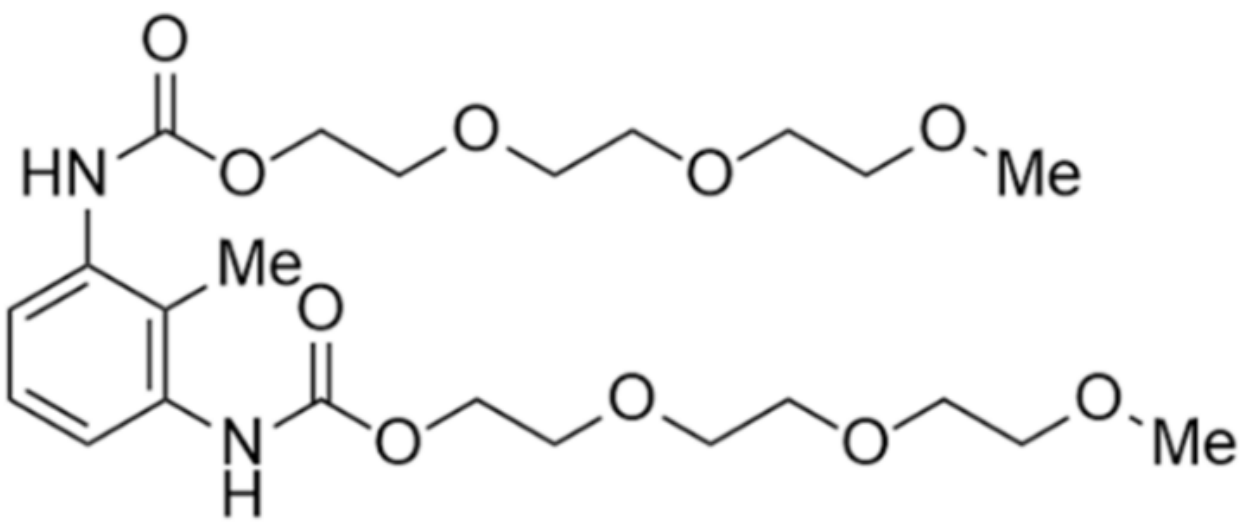

Figure S34. Skeleton structure of DUE-2,6-TDA.

Tolylene-2,6-diisocyanate (DUE-2,6-TDA) (1 mL, 7,00 mmol, 1.00 eq.) was dissolved in triethylene glycol monomethyl ether (10 mL) in a 25 mL round-bottomed flask charged with oval stir bar under a nitrogen atmosphere. The reaction was initiated by addition of 20 µL Dibutyltin dilaurate. The mixture was heated up to 60 °C and stirred continuously for 3 hours, the temperature was then increased to 80 °C and the mixture was stirred overnight. The crude mixture was diluted with 25 mL methylene chloride. The organic phase was washed 5 times using 50 mL demineralized water, and once using Brine. The organic phase was concentrated onto celite under reduced pressure, and purified on a Büchi Pure C-850 FlashPrep (25 g column, heptane:ethyl acetate gradient heptane:ethyl acetate 100:0 to 60:40 in 15 minutes, held at 60:40 for 3 minutes, then gradient from 60:40 to 0:100 in 15 minutes). All relevant fractions were gathered and reduced under reduced pressure to afford the desired product as a yellow oil. (0,71 g, 21 %). ^{1}H NMR (80 MHz, CDCl3): δ 7.45 (m, 2H), 7.30 (m, 1H), 6.64 (br s, 2H), 4,37 (m, 4H), 3,71 (m, 20 H), 3.56 (br s, 6H), 2.17 (s, 3H). ^{13}C NMR (20 MHz, CDCl3): δ 154.2, 136.3, 126.7, 119.9, 72.0, 70.7, 69.6, 64.5, 59.2.
HRMS (ESI+): m/z calcd. for $C_{23}H_{38}N_2O_{10}$ $[M+H]^+$: 503.2599, found 503.2600.

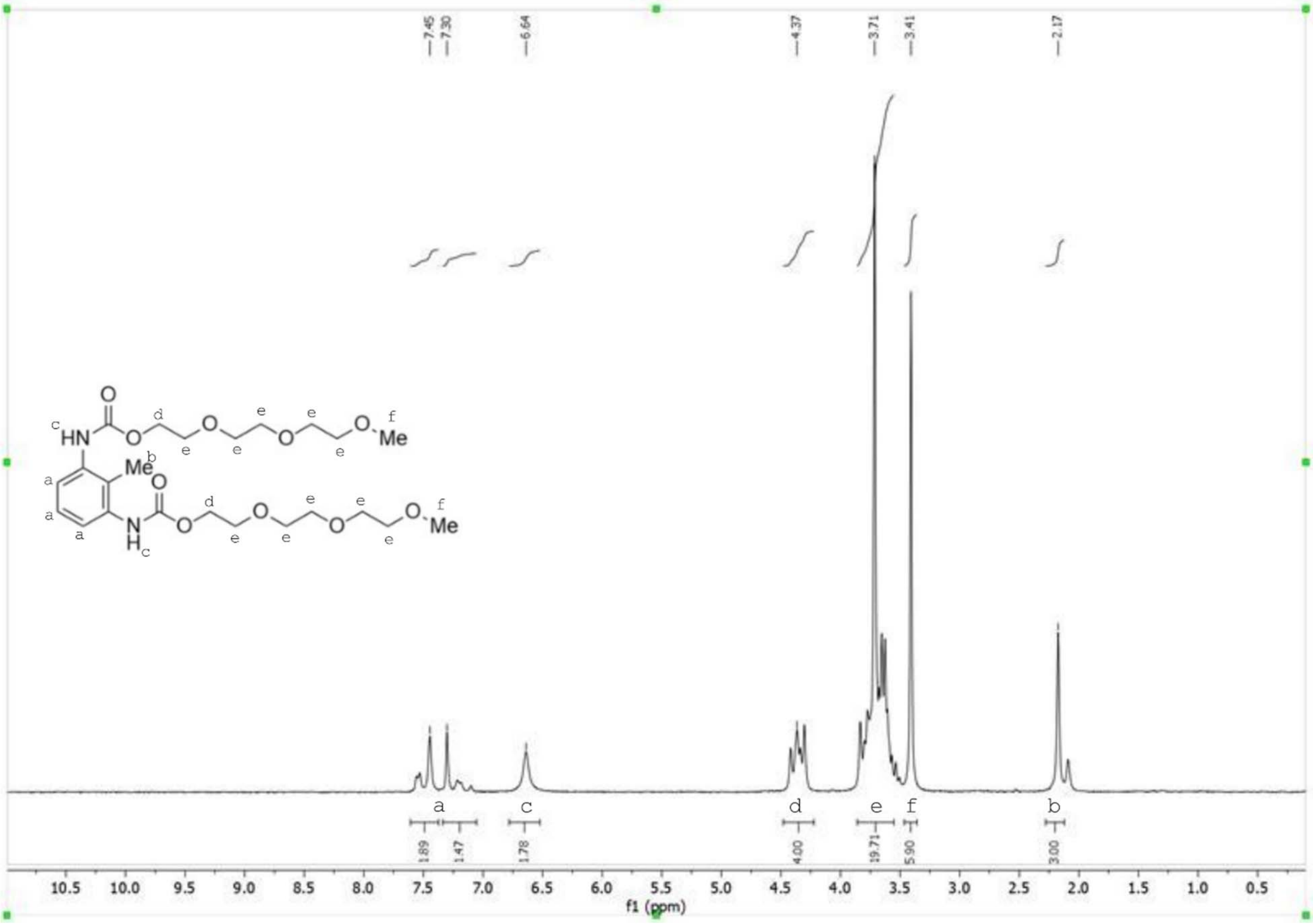

Figure S35. H-NMR spectrum for DUE-2,6-TDA.

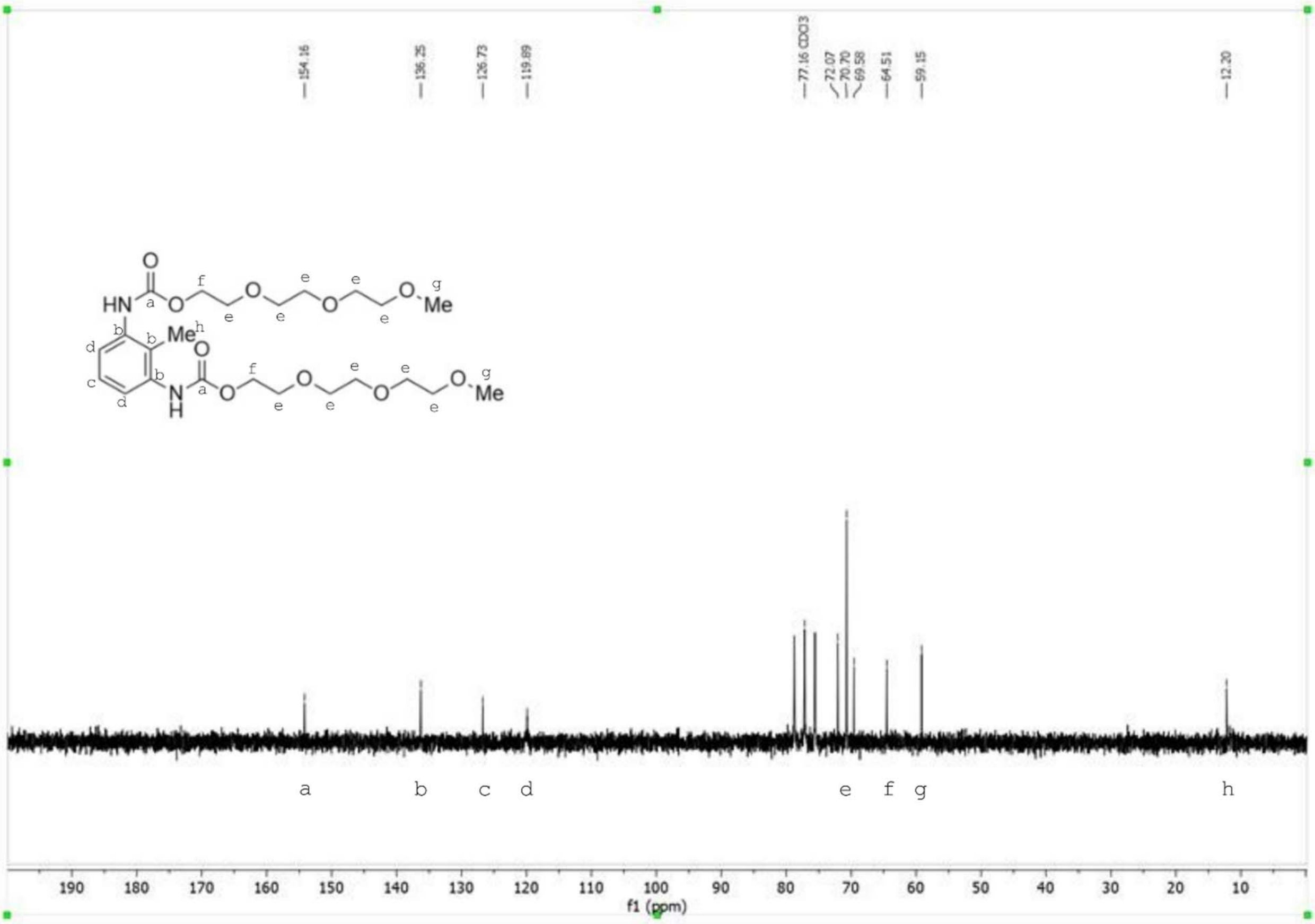

Figure S36. C-NMR spectrum for DUE-2,6-TDA.

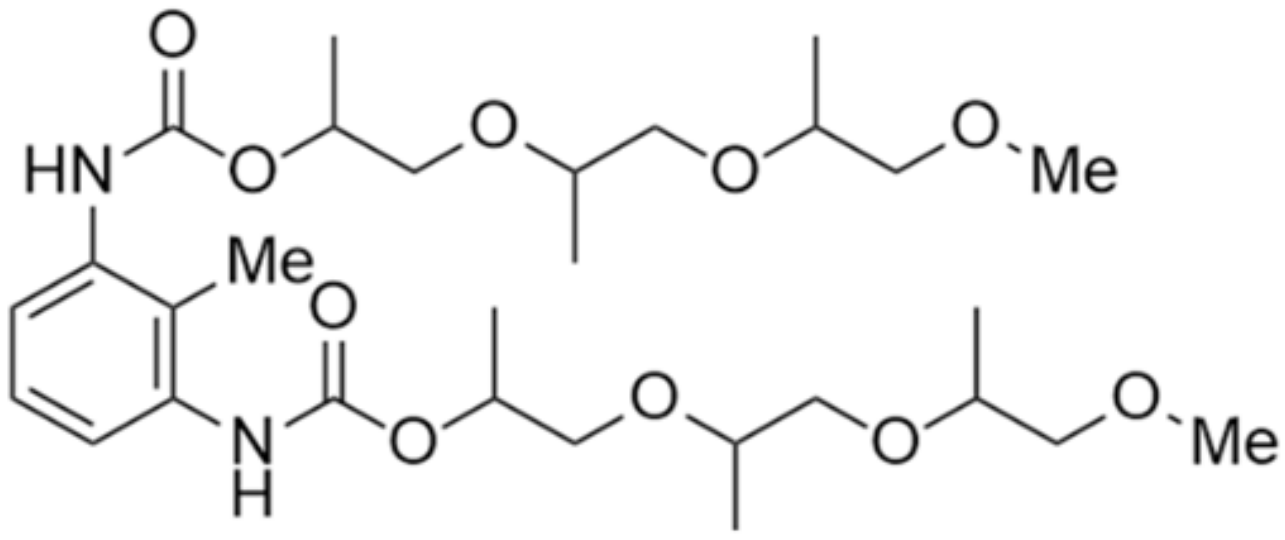

Figure S37. Skeleton structure of DUP-2,6-TDA.

Tolylene-2,6-diisocyanate (DUP-2,6-TDA) (1 mL, 7,00 mmol, 1.00 eq.) was dissolved in tripropylene glycol monomethyl ether (mixture of isomers, 10 mL) in a 25 mL round-bottomed flask charged with oval stir bar under a nitrogen atmosphere. The reaction was initiated by addition of 20 µL Dibutyltin dilaurate. The mixture was heated up to 60 °C and stirred continuously for 3 hours, the temperature was then increased to 80 °C and the mixture was stirred overnight. The crude mixture was diluted with 25 mL methylene chloride. The organic phase was washed 5 times using 50 mL demineralized water, and once using Brine. The organic phase was concentrated onto celite under reduced pressure, and purified on a Büchi Pure C-850 FlashPrep (25 g column, heptane:ethyl acetate gradient heptane:ethyl acetate 100:0 to 60:40 in 15 minutes, held at 60:40 for 3 minutes, then gradient from 60:40 to 0:100 in 15 minutes). All relevant fractions were gathered and reduced under reduced pressure to afford the desired product as a yellow oil. (1.02 g, 25 %). ^{1}H NMR (80 MHz, CDCl3): δ 7.43 (m, 2H), 7.27 (m, 1H), 6.51 (br s, 2H), 5,07 (m, ~2H), 3,71 (m, 22 H), 2.14 (s, 3H), 1.19 (m, 18H). ^{13}C NMR (20 MHz, CDCl3): δ 155.6, 138.0, 128.3, 128.1, 124.1, 121.5, 80.5, 78.9, 78.3, 77.7, 77.3, 76.9, 76.7, 75.0, 74.7, 73.5, 72.7, 72.5, 60.8, 58.4, 18.9, 18.8, 18.7, 18.6, 18.1, 13.8.
HRMS (ESI+): m/z calcd. for $C_{29}H_{50}N_2O_{10}$ $[M+H]^+$: 587.3538, found 587.3562.

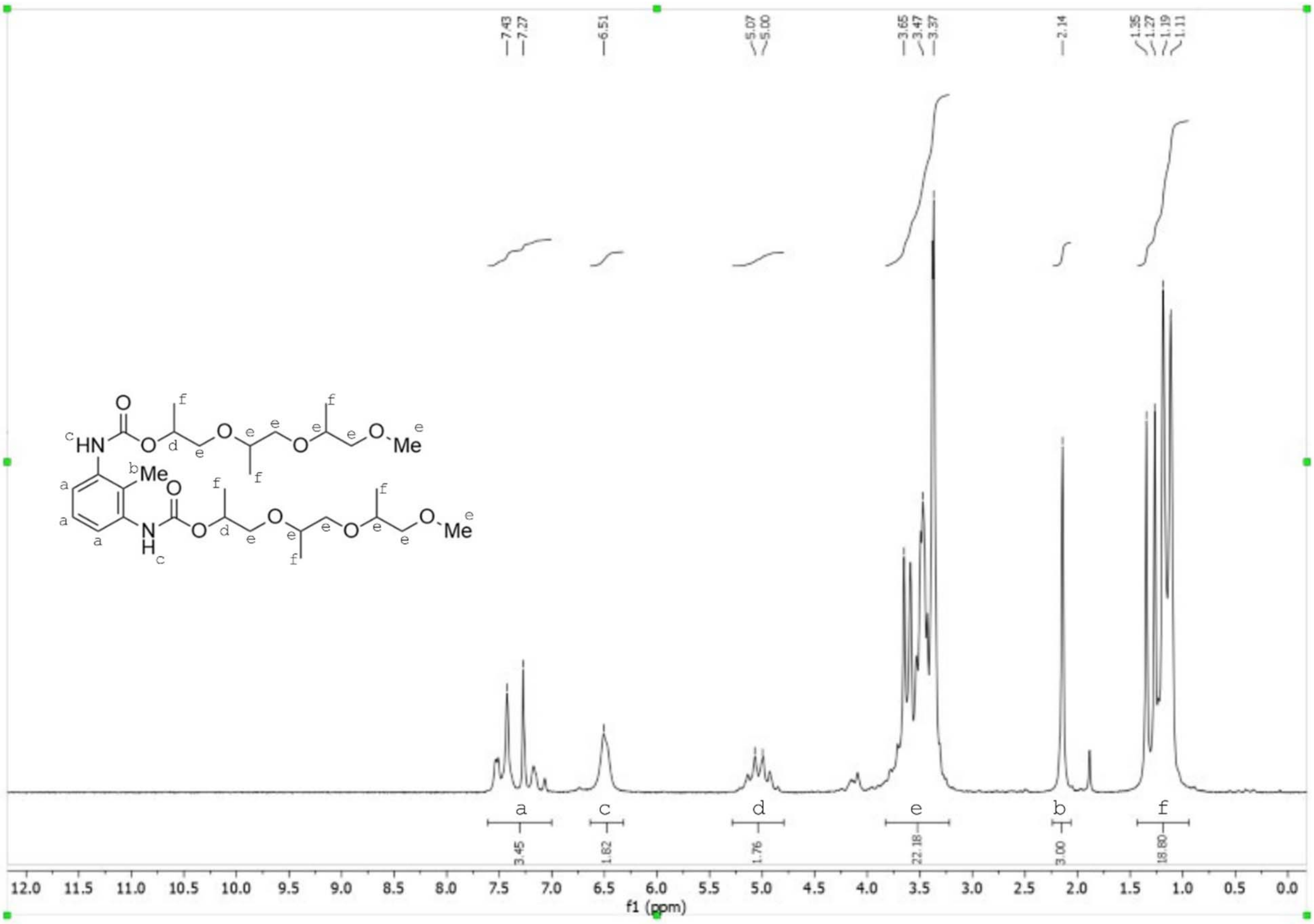

Figure S38. H-NMR spectrum for DUP-2,6-TDA.

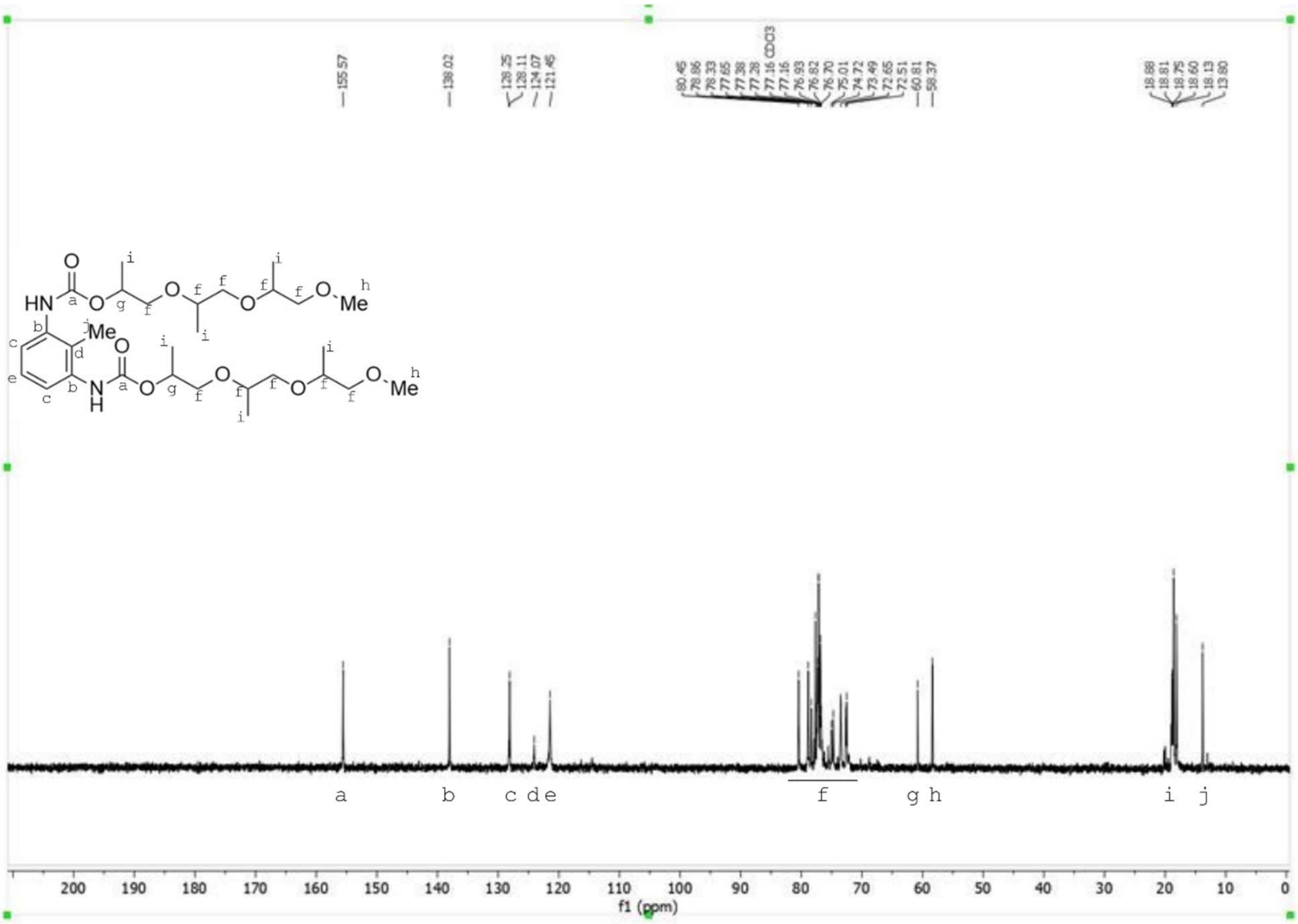

Figure S39. C-NMR spectrum for DUP-2,6-TDA.
